# The Phylogeny of Osteopontin—Analysis of the Protein Sequence

**DOI:** 10.3390/ijms19092557

**Published:** 2018-08-28

**Authors:** Georg F. Weber

**Affiliations:** College of Pharmacy, University of Cincinnati Academic Health Center, Cincinnati, OH 45267-0004, USA; georg.weber@uc.edu; Tel.: +151-3558-0947

**Keywords:** osteopontin, protein, sequence, evolution, taxonomy

## Abstract

Osteopontin (OPN) is important for tissue remodeling, cellular immune responses, and calcium homeostasis in milk and urine. In pathophysiology, the biomolecule contributes to the progression of multiple cancers. Phylogenetic analysis of 202 osteopontin protein sequences identifies a core block of integrin-binding sites in the center of the protein, which is well conserved. Remarkably, the length of this block varies among species, resulting in differing distances between motifs within. The amino acid sequence SSEE is a candidate phosphorylation site. Two copies of it reside in the far N-terminus and are variably affected by alternative splicing in humans. Between those motifs, birds and reptiles have a histidine-rich domain, which is absent from other species. Just downstream from the thrombin cleavage site, the common motif (Q/I)(Y/S/V)(P/H/Y)D(A/V)(T/S)EED(L/E)(-/S)T has been hitherto unrecognized. While well preserved, it is yet without assigned function. The far C-terminus, although very different between Reptilia/Aves on the one hand and Mammals on the other, is highly conserved within each group of species, suggesting important functional roles that remain to be mapped. Taxonomic variations in the osteopontin sequence include a lack of about 20 amino acids in the downstream portion, a small unique sequence stretch C-terminally, a lack of six amino acids just upstream of the RGD motifs, and variable length insertions far C-terminally.

## 1. Introduction

Osteopontin (OPN) is a protein with fundamental functions in biology. While the name of the molecule is a misnomer—it does not critically contribute to the structural integrity of connective tissue or the skeleton [1,2]—it is broadly important for tissue remodeling [1,3], acts as a Th1 inducer cytokine [4], and regulates calcium homeostasis in milk and urine [5,6]. In pathophysiology, osteopontin contributes to the progression of multiple cancers [7,8].

Osteopontin is very versatile. The cytokine is secreted and exerts differential effects on target cells when presented either in solution or after immobilization [9]. Cross-linking to the matrix can occur through transglutamination [10,11]. A variant gene product, generated by translation from an alternative start site eliminates the signal sequence and generates an intracellular form of osteopontin [12]. Further, reuptake after secretion is likely, and the splice variant osteopontin-c accumulates in the nucleus of cancer cells [13].

Despite a large and growing literature on osteopontin in health and disease (well exceeding 9000 publications in PubMed), multiple domains on the protein remain without identified functions. In humans, the far N-terminus contains splice sites that can generate the short forms osteopontin-b and -c [14]. While their patho-biological effects in cancer have recently been elucidated, their direct binding partners remain unknown. The central portion through the thrombin cleavage site harbors several integrin interaction domains, and it is the best understood substructure. The C-terminal portion of osteopontin contains heparin-binding sites and interacts with a variant form of CD44 [15], but large sections are yet unmapped.

To shed more light on the structure-function relationships of osteopontin, we conducted a taxonomic analysis of its protein sequences over a wide spectrum of species (Table S1). From the evolutionary context we inferred conserved and variable domains, some of which have enabled hypotheses regarding their importance in biology.

## 2. Results

### 2.1. Common Structure

**Figure 1:**
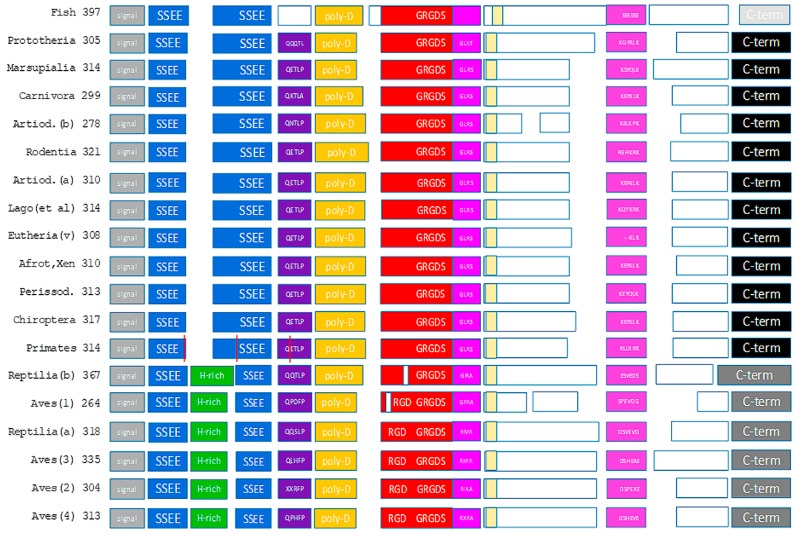
The domain structure of osteopontin. Known subunits are displayed as colored blocks. The vertical red lines in the primates reflect the splice sites in human osteopontin. The unmarked yellow boxes show the newly identified conserved domain without a known function. The numbers indicate the number of amino acids in the canonical sequence. While an effort was made to accurately reflect the size differences across taxonomic groups within each domain, the model is not precisely drawn to scale. Artiod. = Artiodactyla, Lago (et al.) = Lagomorpha and similar species, (v) = (various), Afrot, Xen = Afroteria and Xenarthra, Perissod. = Perissodactyla.

The osteopontin domain structure across species was developed from shared sequence patterns (Figure 1 and Figure S1). Underlying were alignments of the canonical sequences for each taxonomic group under study as well as alignments of sequences for all individual species covered (Table S2).

### 2.2. SSEE, Transglutamination Sites and Poly-Histidine

There are two motifs around the core sequence SSEE in the osteopontin N-terminus. The domain (D/N/S/I)S(G/E/A)SSEE(K/R/L/V)(Y/V/Q/R)(L/R/D) is highly conserved across all species analyzed. Slightly downstream, the second motif, (P/Q)Q(X)(X)(V/Y)SSEE(S/T)(V/A/N/D)D, also has a high level of conservation. In mammals, the second SSEE motif extends upstream to PDAV(A/S)TWLKPDSQKQ(T/N)(L/F)LA and contains a sequence (human WLNPDP) previously reported as critically involved in lymphocyte adhesion, migration and survival [16]. The shorter motif SSEESVD is also present (across multiple species) in ankyrin-2, which is required for the formation or stability of a subset of microtubules in skeletal muscle.

In the stretch SQKQNLLAPQNAVSSEETNDFKQET (*Homo sapiens*), the underlined glutamine residues are candidate substrate sites for the enzyme transglutaminase. Possible target glutamines align in most species. In the human splice variant osteopontin-b, the loss of exon 5 eliminates the second SSEE motif (while preserving DSGSSEEKQLYNKYPDAVATWLNPDPSQKQNLLA) (Table 1A). In the splice variant osteopontin-c, two transglutamination sites are lost, and both SSEE motifs are brought into close proximity in the sequence DSGSSEEKQ-NAVSSEETND around the splice junction (marked by a hyphen). Even though only the splice variant osteopontin-c is abundant in the nucleus [13], there is no apparent nuclear localization sequence, K(K/R)X(K/R), in closeness to the splice junction, and the loss of exon 4 does not generate a candidate bipartite nuclear localization motif. However, homologies resulting from a BLASTP search and a motif search in ELM identify similarities of the perijunctional sequence to nuclear proteins (Table 1B), corroborating that osteopontin-c may have a function in the cell nucleus.

Selectively in Aves and Reptilia, the conserved sequence upstream of the second SSEE motif is replaced by a variable-length histidine-rich domain. It is conceivable that, in evolution, the histidine-rich sequence was acquired from a microbe, such as the avian malaria parasite *Plasmodium lophurae* [17]. Histidine-rich malaria proteins may activate the inflammasome, resulting in decreased integrity of tight junctions and increased endothelial permeability [18], functions that hypothetically could have been acquired by avian osteopontin. (It is notable that, across all species analyzed, a distinct, rather histidine-rich region is located just upstream of the C-terminus, reflected in the primate sequence HEFHSHEFHSH.)

### 2.3. Integrin-Binding Block

**Figure 2:**
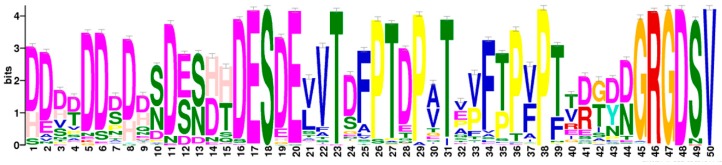
Integrin-binding block. The highest scoring motif (score 23718.7) in gapped local alignment with glam2scan (Meme Suite) covers the downstream portion of the poly-aspartate sequence through GRGDSV. Further, the sequence alignment by glam2scan confirms the varying block sizes from Figure 3.

**Figure 3:**
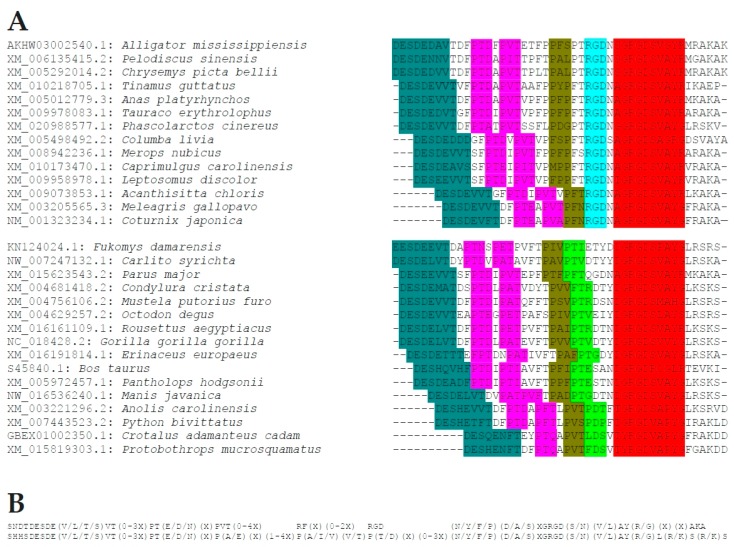
Sequences proximal to the RGD motif. (**A**) Select characteristic sequences for two manifestations of the motif spanning upstream of the canonical RGD domain. The upper block represents Aves and the subgroup of Reptilia that harbor two adjacent RGD sequences. The lower block represents all others. Preserved stretches of amino acids are highlighted with colored background, such that matching motifs are shaded by identical background color. (**B**) The common sequence motifs derived from the two groups of patterns are shown.

While the canonical integrin-binding GRGD(S/N) sequence is 100% conserved in all osteopontins, a common pattern extends substantially upstream (including in humans the integrin α4β1 binding site ELVTDFPTDLPAT [19]), and a short stretch downstream (including in humans the integrin α9β1 binding site SVVYGLR [20]). This proximity places all known integrin binding sites on a highly preserved region of amino acids in the central portion of the protein (Figure 2). Within this stretch, there are common subsequences, but their distances from each other vary among species (Figure 3), generating shorter or longer versions of the integrin-binding block in osteopontin. Whereas higher order species have one RGD motif, two consecutive RGD sequences are present in close proximity to each other within birds, and among the Crocodilia and Testudines (but not the Squamata) orders within the class of Reptilia. Birds also have an incompletely preserved RGD motif about 120 amino acids downstream of the central GRGDSV.

### 2.4. Heparin-Binding Motifs

Following the integrin-binding block is the thrombin cleavage site GLRSKS (thrombin cleaves between R and S). This is also a putative heparin-binding site with the sequence YGLRSKSKKF. A second heparin-binding motif in Primates is marked by RL(Y/H)KRK. Just upstream of it, the sequence QXDD(R/H/Y)S(L/M)ENDSXXXX is quite conserved. In the far C-terminus, the mammalian sequence contains a third putative heparin-binding site with the sequence DKHLKFRI. All putative heparin-interaction motifs of osteopontin are located on the C-terminal domain. While these amino acid stretches display some conservation among Mammals, their aligned sequences in lower species are divergent, lack lysines, and are not likely heparin-binding.

### 2.5. Novel (T/S)EED Domain

**Figure 4:**
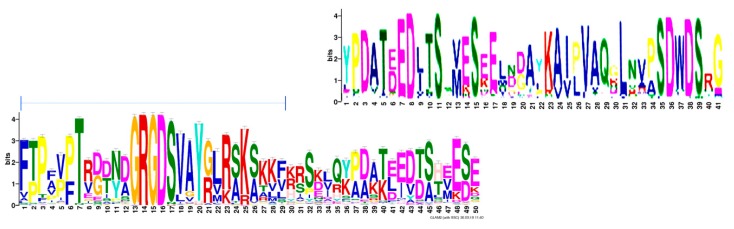
(T/S)EED motif. The overlap between motifs identified in Meme Suite by a Multiple Em for Motif Elicitation (MEME) search (top) or a GLAM2 search (bottom) concurs with the sequence characterized here as well conserved. The horizontal bar over the bottom sequence marks a portion of the integrin-binding block through the thrombin-cleavage/heparin-binding site, which belongs to a separate motif.

Just downstream from the thrombin cleavage site, the common motif (Q/I)(Y/S/V)(P/H/Y)D(A/V)(T/S)EED(L/E)(-/S)T has been hitherto unrecognized. It is obviated by a section of amino acid commonality in sequence alignment (see Table S2.1), and the region is recognized as a motif in distinct searches in Meme Suite (Figure 4). While well preserved across species, it is yet without assigned function (Table 2). Hints to its potential evolutionary origin come from a MOTIF search, according to which the sequence ISYDA(S/T)EEDL (most similar to the domain in group 4 of Aves) is present in the microbial reference gene ID numbers RG001:002553064, RG001:008233923, RG001:010468220. It could have been picked up from microbes by birds, and may have evolved from there.

### 2.6. C-Terminus

The far C-terminus, although very different between Reptilia/Aves, SNQTLESAEDXQD(R/H)HSIEXNEVT(R/L/I), on the one hand and Mammals, D(P/H/R)KS(K/E/V)EEDK(H/Y)LKFR(I/V)SHEL(D/E)SASSEVN, on the other, is very preserved within each class of species. Candidate phosphorylation sites stand out in each and may have functional importance (Table 3). The mammalian sequence contains the putative heparin-binding site DKHLKFRI. The high conservation suggests physiological roles that are distinct between Reptilia/Aves and Mammals but have only been partially mapped. Even though the main integrin-binding site resides in the GRGD(S/N) motif, modification of the extreme C-terminus plays an important regulatory role for the interaction with the integrin αVβ3 [21].

### 2.7. Taxonomy

**Figure 5:**
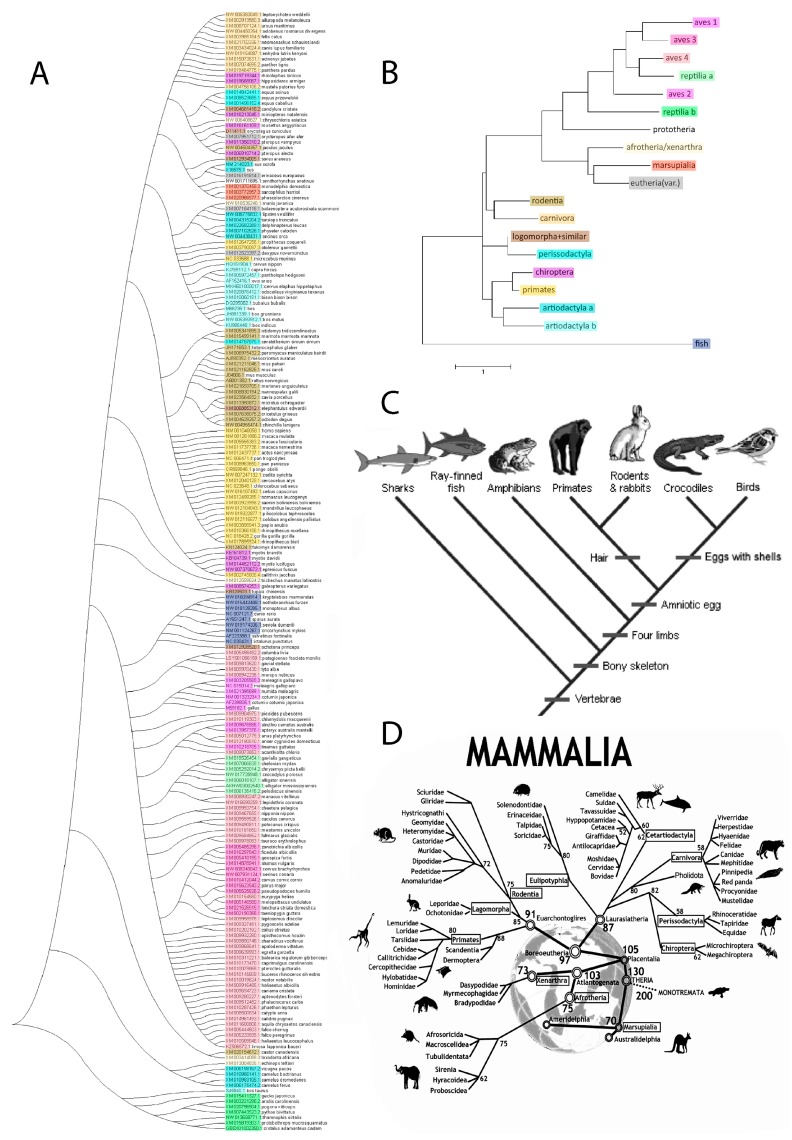
Phylogenetic tree analysis of osteopontin. (**A**) Individual osteopontin sequences. The color coding reflects the taxonomic affiliation as displayed in (B). The tree with the highest log likelihood (−1039.72) is shown. The tree is drawn to scale, with branch lengths measured in the number of substitutions per site. The analysis involved 202 amino acid sequences. All positions containing gaps and missing data were eliminated. There were a total of 17 positions in the final dataset. (**B**) Canonical osteopontin sequences. The tree with the highest log likelihood (−7192.43) is shown. The analysis involved 19 amino acid sequences. All positions containing gaps and missing data were eliminated. There were a total of 156 positions in the final dataset. (reptilia A = Crocodilia, Testudines; reptilia B = Squamata; artiodactyla a = Camelidae, Suidae, Celaceae; artiodactyla b = Cervidae, Bovidae). (**C**) Evolutionary relationships of major vertebrate groups as a reference point. Adopted from the University of California Museum of Paleontology’s Understanding Evolution (https://evolution.berkeley.edu/evolibrary/search/imagedetail.php?id=251&topic_id=&keywords=phylogeny). (**D**) An evolutionary tree of Mammals as a reference point. The tree depicts historical divergence relationships among the living orders of Mammals. The phylogenetic hierarchy is a consensus view of several decades of molecular genetic, morphological and fossil inference. Double rings indicate mammalian supertaxa, numbers indicate the possible time of divergences [23]. This file has been reproduced from https://commons.wikimedia.org/wiki/File:An_evolutionary_tree_of_mammals.jpeg under the Creative Commons Attribution 2.0 Generic license.

A tree diagram corroborates the distribution by phylogenetic relatedness and roughly aligns with established trees of life. The broad pattern of the sequence alignment (all 202 sequences) groups Eutheria together, while the lower species comprising Reptilia and Aves cluster in a slightly different pattern. Deviating from both are Fish, which have their unique osteopontin forms. Substantial differences in osteopontin between the two subgroups of Reptilia are evident, as well as their proximity to the four subgropus of Aves. Surprisingly, the analysis of individual sequences as well as canonical sequences places the osteopontins by a subset of Chiroptera closely to the primates (Figure 5). The canonical sequence for Chiroptera (317 amino acids) shares 244 identities with the canonical Primate sequence (314 amino acids). By contrast, the evolutionarily much closer Carnivora (299 amino acids) share only 200 identities. It is important to note, however, that various algorithms produce somewhat differing results (Figure S2).

**Figure 6:**
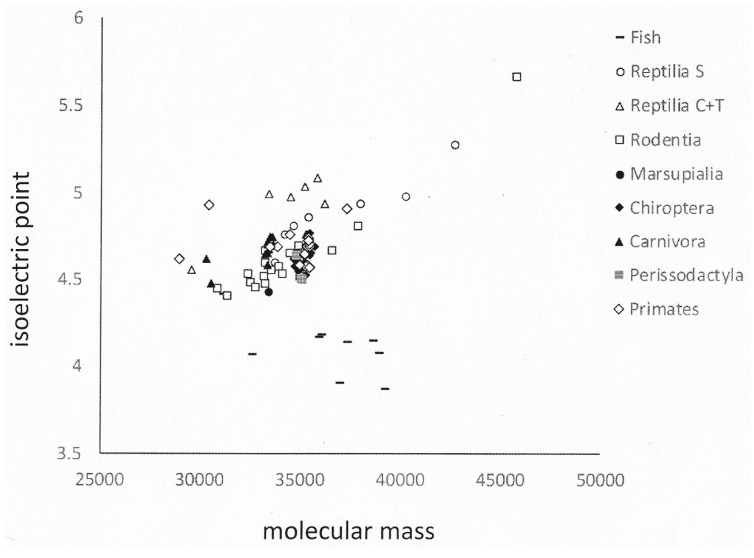
Physico-chemical properties of osteopontin in various orders of species. The graph shows isoelectric point versus molecular mass for each available member of nine orders of species. Fish osteopontin is very different from others, which is reflected in its separation. In general, higher order organisms (including Carnivora, Primates) cluster more tightly than lower orders (such as Rodentia).

Whereas the lesser evolved species (Rodentia, Reptilia/Squamata, Fish) show a non-trivial extent of variation in their osteopontin sequences, among the higher organisms osteopontin is well conserved within taxonomic groups. This is evidenced in their increasing percent homology (Table 4) as well as in their physico-chemical characteristics of molecular weight, isoelectric point and amino acid composition, where higher organisms cluster more tightly than the lower level orders (Figure 6 and Figure S3). Once a mutation has taken place in evolution, its penetration of the population is subjected to the rule of selection. However, complex systems can exhibit powerful self-organization, and the effects of mutation and selection are diminished when operating on organisms that have their own rich and robust self-ordered properties. Spontaneous order is well maintained [22]. The available osteopontin sequences seem to corroborate this principle.

We sorted the 202 osteopontin sequences by taxonomic affiliation. The small groups of Afrotheria and Xenarthra are within close proximity on the phylogenetic tree of mammals and were analyzed together. Among the Artiodactyla, the sequences of the Cervidae and Bovidae differ substantially from the Camelidae, Suidae and Celacea. These subgroups were evaluated separately. Among the Reptilia, the Cocodilia and Testudines group together tightly, whereas the Squamata are very different and internally less conserved. The large group of Aves (63 available sequences) contains 4 distinguishable subgroups (see Table S1).

Deviations from the bulk of available sequences were found in select groups. A subset of Artiodactyla, comprising Cervidae and Bovidae, lack a sequence of about 20 amino acids in the downstream portion of osteopontin. A subgroup of Birds (*Gallus*, *Meleagris*, *Coturnix*, *Numida*) have a small unique sequence stretch in their C-terminus, and they lack 6 amino acids just upstream of the two consecutive RGD motifs. Another subgroup (*Lonchura*, *Taeniopygia*, *Corvus*, *Parus*, *Pseudopodoces*, *Sturnus*, *Ficedula*, *Serinus*, *Geospiza*, *Zonotrichia*) has variable length insertions far C-terminally. There is a far N-terminal histidine-rich insert in the Squamata, extending a histidine-rich region that is present only in Reptilia and Aves (see Table S2).

(E/Q)TLP(S/D) marks the start of exon 6 and constitutes the beginning of the central osteopontin portion, which extends to the thrombin cleavage site. This portion is present in all spliced forms, -a, -b and -c. The highest variation among representatives within a taxonomic group (as evaluated in Table S1) is always in the poly-aspartate region directly downstream of (E/Q)TLP(S/D). In repetitive stretches of DNA, DNA polymerases are subject to slippage, which may increase the mutation rate during reduplication. It appears that the length of the poly-aspartate domain has increased throughout evolution (see Table S2).

### 2.8. Osteopontin Variants

This study has examined the canonical full-length protein sequences of osteopontin (for a structural analysis and graphic depiction of variant osteopontin forms in humans, see [24]). Numerous osteopontin transcript variants are listed for many species in NCBI nucleotide. They are almost invariably predicted by computer algorithms, not observed by wet-lab analysis. Experimentally, splicing has been confirmed only in humans [14,25], and there it has not yet been shown conclusively to occur in healthy tissues (splicing is associated with cancer progression). The existence of osteopontin splice variants remains to be demonstrated in other species.

In mice, an alternative start site was described, translation from which eliminates the signal sequence and generates an intracellular form of osteopontin [12]. The report also identified potential additional alternative start sites further downstream. In addition, the human variant form osteopontin-5 has been described, which retains an extra exon, located between the canonical exons 3 and 4, and gives to the isoform an alternative translation start, thus yielding a larger protein [24]. Similar mechanisms may also be utilized by other species, and several sequences in NCBI nucleotide seem to reflect such variants. They include replacements of the signal sequence (*Cervus elaphus hippelaphus*, *Dasypus novemcinctus*), replacements of the N-terminal sequence until the start of exon 4 (*Colobus angolensis palliates*, *Motis lucifugus*, *Chinchilla lanigera*), sequence start at exon 4 (*Odocoileus virginianus texanus*), and a unique N-terminal stretch that extends downstream to the second SSEE motif (*Limosa lapponica baueri*) (see alignments in Table S1). However, here experimental validation is required as well.

Most bird osteopontins are in the database with a short and a long sequence (Table S3). It is likely that the short sequence represents a flaw in commonly used prediction algorithms that artifactually terminate the protein prematurely. Notably the curated sequences are all consistent with full-length osteopontin.

Genbank contains several entries of “osteopontin-like” proteins. Further, we have found sequences listed under the designation “osteopontin” to lack sufficient homology for being compatible with actual osteopontin. Not included in the present analysis are MCFN01000076.1: *Callipepla squamata* (Bird), AWGT02000006.1: *Colinus virginianus* (Bird), KX833902.1: *Polypterus senegalus* (Fish), XM_006007328.1: *Latimeria chalumnae* (Fish), LZPO01073101.1: *Neotoma lepida* (Rodent), NW_017857686.1: *Aedes albopictus* (Insect), NW_019106283.1: *Nilaparvata lugens* (Insect), NW_017955625.1: *Aegilops tauschii* (Plant), NW_017617580.1: *Ipomoea nil* (Plant), NW_019683307.1: *Lactuca sativa* (Plant), KB317696.1: *Rhizoctonia solani* (Fungus), ASPP01001874.1: *Reticulomyxa filose* (Fungus), XM_003074107.2: *Ostreococcus tauri* (Bacterium).

## 3. Discussion

In this study, we have characterized the phylogeny of osteopontin, based on the hypothesis that domains with a high level of conservation among species will be reflective of important biological functions being fulfilled by these regions. This analysis has helped to refine the understanding of known domains, and to identify one previously uncharacterized domain.

Although osteopontin is deemed to be a largely unstructured protein, considerable portions of the molecule represent well characterized functional entities (integrin- and heparin-binding sites). Major additional portions are much preserved across species (SSEE phosphorylation sites, (T/S)EED domain, thrombin cleavage site, C-terminus) (see Figure 1), implying important shared functions. The conservation of such features suggests that osteopontin in situ may assume very refined conformations, likely facilitated through its interaction partners. Only the C-terminal thrombin cleavage fragment of the molecule has extended sequences that are unmapped (white boxes in Figure 1). Any release from conformational constraints is most likely to reside in those domains.

Molecular evolution has been facilitated by genomic plasticity, including the likelihood that organisms can incorporate foreign DNA [26,27]. In Aves, two osteopontin sequence stretches imply such a possibility, entailing the histidine-rich insertion between the SSEE motifs as well as the newly identified domain. The histidine-rich sequence could have been acquired from a microbe, such as the avian parasite *Plasmodium lophurae* [17], the genome of which encodes a histidine-rich malaria protein. Because the highly repetitive stretch of consecutive histidines does not allow a meaningful analysis by sequence alignment, the proposition that the Reptilian and Avian histidine-rich domain may have microbial origin remains a hypothesis. On the other hand, a motif search pointed to similarity between microbial genes and a stretch in the newly identified motif, especially for a subgroup of Aves. It suggests an origin by horizontal gene transfer.

Osteopontin is subject to substantial posttranslational modifications, such as glycosylation, phosphorylation, and calcium binding. It may be cleaved by various proteases, including thrombin, MMP-3, MMP-7, cathepsin-D and plasmin. All of these functions are tied to specific sites. While an examination of the evolutionary conservation in their facilitating motifs may yield important information on osteopontin biology or patho-biology, it is beyond the scope of the present report.

Few and succinct analyses of phylogenetic trees for osteopontin are in the literature. In a sequence comparison of osteopontin in GenBank between yak and cattle, buffalo, sheep, goat, pig, human, and rabbit, the yak sequence had identity of 52–99% and similarity of 65–99% in deduced amino acids [28]. Yak osteopontin had higher homology in both nucleotide and amino acid sequences with cattle than with the other species analyzed. Another study compared the amino acid sequences of osteopontin derived from human, mouse, rat, rabbit, water buffalo and cattle. The protein was divided into 9 regions, of which only five had known functions. These were poly-aspartate (binds calcium), RGD (engages integrins), GLRS (is a thrombin cleavage site), and calcium and heparin binding sites in the distal domains. The human and rabbit sequences had 64% similarity whereas human and chicken only had a 21% similarity score in multiple sequence alignment. Distinct differences were found between human and chicken, which could reflect functional and developmental differences between avian and mammalian osteopontin [29]. In a study of wild boar osteopontin, amino acid similarities were evaluated with human, chimpanzee, rhesus monkey, cattle, water buffalo, sheep, domestic pig, Norway rat, house mouse, chicken and zebrafish. Common motifs included the signature sequence (including SSEEK), substrates for transglutaminase (glutamines), the poly-aspartate domain, GRGDS (sequence for cell attachment), a site of thrombin cleavage, and potential sites for phosphorylation by acidotrophic casein kinases I and II. Highly conserved sequences entailed 7 or 10 residues in the poly-aspartate region, an SSEEK motif, a GRGDS motif (and some 50 amino acids bracketing the RGD sequence), a RS or KS in most species except chicken and zebrafish, and the NH_2_- and COOH-terminal regions. Zebrafish and chicken were located at the bottom of the tree, chimpanzee and human existed in the treetop. The wild boar was located more closely to cattle and sheep. Consistent with their evolutionary distance, zebrafish (the lowest vertebrate in this analysis) was far from the others [30]. A *Sparus aurata* gene encodes a 374 amino-acid protein, which contains domains that are characteristic of osteopontin. They include an integrin-binding RGD motif, a negatively charged domain, and sites for post-translational modifications. The common origin of Mammalian osteopontin and Fish osteopontin-like proteins was inferred from an in-silico analysis of available sequences. It revealed similar gene and protein structures and was corroborated by their specific expression in mineralized tissues and cell cultures [31].

## 4. Materials and Methods

### 4.1. Source of Sequences

A search in NCBI nucleotide for the keyword osteopontin yielded 2522 hits. All were screened for actual osteopontin sequences. Duplicate entries were eliminated, and only the longest of multiple potential transcripts were included in the comparisons. The resultant 202 sequences were grouped by shared taxonomy (Table S1). In order to be able to evaluate functional motifs, the analysis utilized protein sequences, not DNA.

### 4.2. Alignment

The sequences were aligned using Clustal Omega by EMBL-EBI (https://www.ebi.ac.uk/Tools/msa/clustalo/) at the default settings. For the alignment of the canonical sequences, manual adjustments were made.

The quantitative assessment of homologies was accomplished with the TreeTop function in Gene Bee (http://www.genebee.msu.ru/services/phtree_full.html). The analysis applied cluster and topological algorithms; it considered columns with unknown amino acids. The cluster algorithm for unrooted tree with scaled branches had the max/min factor set to 8 (the default value).

### 4.3. Phylogenetic Tree

Phylogenetic relatedness evaluation and presentation in tree diagrams was done using MEGA7 (https://www.megasoftware.net/). The MEGA (Molecular Evolutionary Genetics Analysis) software contains methods and tools for phylogenomic analysis.

The evolutionary history was inferred by using the Maximum Likelihood method based on the JTT matrix-based model [32]. The tree with the highest log likelihood was selected. Initial tree(s) for the heuristic search were obtained automatically by applying Neighbor-Joining and BioNJ algorithms to a matrix of pairwise distances estimated using a JTT model, and then selecting the topology with superior log likelihood value. Evolutionary analyses were conducted in MEGA7 [33].

### 4.4. Motif Search

Motifs were identified by scrutiny of the aligned sequences, with a focus on the conserved regions and on the basis of existing knowledge about osteopontin structure and function.

Homologies were assessed with BLASTP 2.8.0 (https://blast.ncbi.nlm.nih.gov/Blast.cgi?PROGRAM=blastp&PAGE_TYPE=BlastSearch&LINK_LOC=blasthome), utilizing the blastp (protein-protein BLAST) algorithm and searching non-redundant protein sequences. This query reviews all non-redundant GenBank coding sequence (CDS) translations plus PDB plus SwissProt plus PIR plus PRF, while excluding environmental samples from whole genome sequencing (WGS) projects.

In addition, linear motifs were mapped in the ELM (Eukaryotic Linear Motif Resource) database (http://elm.eu.org/). The prediction tool scans submitted protein sequences for matches to the regular expressions defined in ELM. Distinction is made between matches that correspond to experimentally validated motif instances already curated in the ELM database and matches that correspond to putative motifs based on the sequence.

Further motif mapping was done in The Meme Suite (http://meme-suite.org/), using the MEME or GLAM2 functions for motif discovery. In MEME (Multiple Em for Motif Elicitation), searches were performed by varying the number of expected motifs. The GLAM2 (Gapped Local Alignment of Motifs, version 2) function searches for gapped motifs in DNA or protein datasets [34] and was used in the default settings.

MOTIF (http://www.genome.jp/tools/motif/) is a GenomeNet database resource that searches with a profile or a protein sequence pattern against protein sequence databases. GenomeNet is a Japanese network of database and computational services for genome research and related research areas in biomedical sciences, operated by the Kyoto University Bioinformatics Center.

## 5. Conclusions

Osteopontin is important for tissue remodeling, cellular immune responses, and calcium homeostasis. In pathophysiology, the biomolecule contributes to the progression of multiple cancers. Despite a rapidly growing literature on the subject, the multiple functions of osteopontin have been incompletely elucidated. Here, we have taken a taxonomic approach to the analysis of the protein structure. We have found numerous highly conserved features, and one previously overlooked domain. These insights will aid in focusing future structure-activity analysis.

## Figures and Tables

**Table 1:** Homologies at the osteopontin-c splice junction.

**A**					
**Splice Variant**		**Junction**			
OPNa		SGSSEEKQ LYNKYPDAVATWLNPDPSQKQNLLAPQ NAVSSEETNDFKQ ETLPSKS			
OPNb		SGSSEEKQ LYNKYPDAVATWLNPDPSQKQNLLAPQ ETLPSKS			
OPNc		SGSSEEKQ NAVSSEETNDFKQ ETLPSKS			
**B**					
		***Homo sapiens***	**Osteopontin-c**	DSGSSEEKQNAVSSEETND	**Nuclear**
BLASTP	EJP73436.1	*SAR86 cluster bacterium*	ribosomal protein L25	DEGSSEEQQN---SEETSE	
	XP_016425206.1	*Sinocyclocheilus rhinocerous*	G2 and S phase-expressed protein 1	ESESQEEKQTSVSNEET	
	XP_016313011.1	*Sinocyclocheilus anshuiensis*	G2 and S phase-expressed protein 1-like	ESESQEEKQTSVSNEET	
	WP_035346390.1	*Bacillus hemicellulosilyticus*	carbohydrate ABC transporter substrate-binding protein	DS-SNEPSENDVSSEETND	
	WP_092544273.1	*Zunongwangia mangrovi*	DUF1343 domain-containing protein	SSEKKQDSVNSEET	
	XP_016350400.1	*Sinocyclocheilus anshuiensis*	protein P200-like	SGSPAERQNPVASEET	
	PNY04530.1	*Trifolium pratense*	GDSL esterase/lipase	GNSEEKENFVSSSET	
	EJP72445.1	*SAR86 cluster bacterium*	dihydrolipoyllysine-residue acetyltransferase	EEK-N-VSSEETND	
	XP_018304503.1	*Trachymyrmex zeteki*	putative inhibitor of apoptosis	SGSS---QNSISSEITND	
	XP_010124132.1	*Chlamydotis macqueenii*	MAX gene-associated protein	GSSEEKEDSVSSE	+
	XP_010079896.1	*Pterocles gutturalis*	MAX gene-associated protein	GSSEEKENSVSS	+
ELM	DOC_USP7_MATH_1		USP7 MATH domain binding motif variant (MDM2 and p53 interactions)	AVSSE	+
	MOD_CK2_1		CK2 phosphorylation site	NAVSSEE	(+)
	MOD_GSK3_1		GSK3 phosphorylation recognition site	NAVSSEET	(+)
	MOD_CK1_1		CK1 phosphorylation site	SGSSEEK	(+)
	MOD_CK2_1		CK2 phosphorylation site	DSGSSEE	(+)
	LIG_TRAF2_1		Major TRAF2-binding consensus motif	SSEE	
	DEG_Nend_UBRbox_2		N-terminal motif that initiates protein degradation	DS	
	MOD_GlcNHglycan		Glycosaminoglycan attachment site	DSGS	

(**A**) Perijunctional sequences for osteopontin-a compared to the splice variants-b and -c. (**B**) Homologies to the splice junction of osteopontin-c according to BLASTP and to ELM. The top row shows the search sequence. In the last column, + indicates nuclear localization, (+) indicates that the location of the match may be nuclear or cytosolic.

**Table 2:** Homologies at the (T/S)EED domain of osteopontin.

**A**				
**Motif**	**Order**			
QYPDATDEDI-T	Primate			
QSPDATEEDF-T	Artiodactyla b			
QYPDSTEEDF-T	Carnivora			
QYPDATEEDL-T	Chiroptera			
QVHDVTEEDL-T	Marsupialia			
QGHDAS-DDF-T	Prototheria			
IVHDATEEDDST	Reptilia a			
DSHDVSDEFDST	Reptilia b			
IEDDATAEVG--	Aves 1			
IXYDATEEDESA	Aves 4			
VHSDLLEEDTST	Fish			
**B**				
			**motif**	
			**(Q/I)(Y/S/V)(P/H/Y)D(A/V)(T/S)EED(L/E)(-/S)T**	
**redox reactions**				
WP_010917268.1	Thermoplasma	radical SAM protein	YPDETDEDI	generates radicals by close proximity of a 4Fe-4S cluster and S-adenosylmethionine
WP_077813583.1	Acetobacter	(2Fe-2S)-binding protein	YPDPTDEDI	Ferredoxins are iron-sulphur proteins that mediate electron transfer
WP_101432357.1	Bifidobacterium	NAD-dependent succinate-semialdehyde dehydrogenase	YPDATDED	oxidoreductase acting on donor aldehyde or oxo group with NAD+ or NADP+ as acceptor
WP_089241263.1	Belliella	OsmC family peroxiredoxin	SPDATEEEF	osmotically induced, preferentially metabolizes organic over inorganic hydrogen peroxide
APR84224.1	Minicystis	Oxidoreductase, short chain dehydrogenase/reductase	PDATEEDF	NAD(P)(H)-dependent oxidoreductase
ODM17387.1	Aspergillus	Delta-1-pyrroline-5-carboxylate dehydrogenase	PDATEEDF	oxidoreductase, acting on the CH-NH group of donors with NAD+ or NADP+ as acceptor
**nuclear structure/function**				
WP_069109061.1	Jiangella	helix-turn-helix domain-containing protein	SPDSTEEDF	helix-turn-helix motif, contained in DNA binding proteins that regulate gene expression
KYN05282.1	Cyphomyrmex	X-ray repair cross-complementing protein 5	PDATDDDIT	single-stranded DNA-dependent ATP-dependent helicase
XP_017256983.1	Daucus	mitotic spindle checkpoint protein BUBR1	SPKATEEDFT	control of cell division
**others**				
WP_100323843.1	Xanthomonadaceae	3-oxoacyl-ACP synthase III	SPEATEEDF	acyl-transferase that participates in fatty acid biosynthesis
WP_019509474.1	Pleurocapsa	1-acyl-sn-glycerol-3-phosphate acyltransferase	QYPDATDDQI	converts lysophosphatidic acid into phosphatidic acid by incorporating an acyl moiety
WP_056956354.1	Lactobacillus	aryl-phospho-beta-d-glucosidase	PDATEEDF	catalyzes the hydrolysis of aryl-phospho-beta-d-glucosides
WP_101098638.1	Stenotrophomonas	VacJ family lipoprotein	PDATEDDFT	contributes to virulence, affects outer membrane and contributes to serum resistance
WP_018890097.1	Streptomyces	ABC transporter ATP-binding protein	PDATDEEIT	ATPase activity, coupled to transmembrane movement of substances
XP_011594169.1	Aquila	unconventional myosin-IXb	QSPDATEEE	intracellular movements, binds actin, inhibited by calcium, GTPase activator for RHOA
WP_056534355.1	Bacillus	DUF1836 domain-containing protein	HDVTEEDLT	domain of unknown function
WP_072744531.1	Sporanaerobacter	Stk1 family PASTA domain-containing Ser/Thr kinase	QVHNVTEENL	StkP activation and substrate recognition depend on the PASTA domain
XP_011141917.1	Harpegnathos	UDP-glucuronosyltransferase 2B15	VHDVTEEKLT	glucuronidation of various xenobiotics and endogenous estrogens and androgens
XP_019462595.1	Lupinus	ubiquitin carboxyl-terminal hydrolase 27	QVHDVSEED	Deubiquitinase, reduces BCL2L11/BIM ubiquitination and stabilize BCL2L11
XP_020978497.1	Arachis	glutamate receptor 3.6	PDATDEEIT	cell surface receptor

(**A**) Canonical sequences for the taxonomic subgroups analyzed. (**B**) Homologies in BLASTP searches. The upper row shows the consensus motif.

**Table 3:** Homologies at C-terminus of osteopontin.

**A**				
**Class**		**Motif**		
Reptilia/aves		SNQTLESAEDXQD(R/H)HSIEXNEVT(R/L/I)		
mammals		D(P/H/R)KS(K/E/V)EEDK(H/Y)LKFR(I/V)SHEL(D/E)SASSEVN		
				
**B**				
		**Aves**	**Osteopontin**	SNQTLE--SAEDAEDRHSIENNEVTR
BLASTP	OBT50472.1	*Pseudogymnoascus* sp. *24MN13*	membrane proton-efflux P-type ATPase	LEVGNAE-AEDRRSIANNE
	WP_083469460.1	*Methylobacterium variabile*	right-handed parallel beta-helix repeat-containing protein	AESAENR--IENNDVT
	WP_037618789.1	*Streptomyces aureus*	ribonuclease E/G	LE--SAEDAED--AVEGDE
	XP_011461236.1	*Fragaria vesca* subsp. *vesca*	inner membrane protein PPF-1, chloroplastic	QTLA--SASDSEDRSDDENND
	XP_018138390.1	*Pochonia chlamydosporia* 170	response regulator	AADTEHRHSIDTNMVTR
ELM	LIG_BIR_II_1		abrogation of caspase inhibition by IAPs in apoptotic cells	SNQT
	MOD_CK1_1		CK1 phosphorylation site	SNQTLE--S
	MOD_GlcNHglycan		Glycosaminoglycan attachment site	E--SAE
	MOD_N-GLC_1		Generic motif for N-glycosylation	SNQTLE
	MOD_Plk_1		Ser/Thr residue phosphorylated by the Plk1 kinase	SNQTLE--S
	MOD_PKA_2		Secondary preference for PKA-type AGC kinase phosphorylation	DRHSIEN
	MOD_Plk_2-3		Ser/Thr residue phosphorylated by Plk2 and Plk3	DRHSIEN
**C**				
		**Primates**	**Osteopontin**	DPKSKEEDKHL-KFRISHELDSASSEVN
BLASTP	WP_034747182.1	*Chryseobacterium vrystaatense*	AraC family transcriptional regulator	KEEDKNL-SFRI-FDLDS
	KUG41143.1	*Pseudomonas savastanoi pv. Fraxini*	Methyl-accepting chemotaxis protein	KHLKH SIS-ELDAAGSELN
	AAP88241.1	*Human beta herpesvirus* 5	UL74 protein	KAKEEERQL-KLRILQELAS
	WP_077317536.1	*Virgibacillus proomii*	acetate-CoA ligase	EDKYINYR---QEMEAASSE
	WP_039995451.1	*Paraglaciecola agarilytica*	ATP-dependent protease	DRRSVLEEQYLPNILVSHELES
	WP_094346596.1	*Peltigera membranacea cyanobiont*	ATP-binding protein	VENDNYL-KFSASNQLE
	WP_104809105.1	*Polaribacter filamentus*	DUF2867 domain-containing protein	EDDKHL-NFRIS
ELM	CLV_PCSK_SKI1_1		Subtilisin/kexin isozyme-1 (SKI1) cleavage site	KHL-KF
	DOC_CYCLIN_1		interacts with cyclin, increases phosphorylation by cyclin/cdk complexes	KHL-KF
	DOC_PP1_RVXF_1		Protein phosphatase 1 catalytic subunit (PP1c) interacting motif	EDKHL-KFR
	DOC_USP7_UBL2_3		USP7 CTD domain binding motif variant	KEEDK
	LIG_TRAF2_1		Major TRAF2-binding consensus motif	SKEE
	MOD_SUMO_rev_2		Inverted version of SUMOylation motif recognized for modification by SUMO-1	DPKSKEEDKHL
	MOD_CK2_1		CK2 phosphorylation site	DPKSKEE
	MOD_CK1_1		CK1 phosphorylation site	SASSEVN
	MOD_PKA_2		Secondary preference for PKA-type AGC kinase phosphorylation.	FRISHEL
	MOD_GlcNHglycan		Glycosaminoglycan attachment site	DSAS

(**A**) Canonical sequences for Aves/Reptilia and for Mammals. (**B**) Homologies to the avian C-terminus of osteopontin according to BLASTP and to ELM. The top row shows the search sequence. (**C**) Homologies to the primate C-terminus of osteopontin according to BLASTP and to ELM. The top row shows the search sequence.

**Table 4:** Extent of osteopontin sequence homologies across species.

			*N*	Refined Alignment		Draft Source Alignment		Phylogenetic Tree
				Power	Homology (%)	Power	Homology (%)	Cluster Algorithm
	Fish		9	154.94	42.30	51.78	14.80	0.999999
Aves and Reptilia			79	1252.13	38.60	784.90	24.80	0.999999
	Aves		64	1348.34	51.00	815.27	31.50	0.744865
		Aves 1	4	109.38	92.70	109.30	92.60	0.000001
		Aves 2	3	62.42	69.50	61.93	68.70	0.000001
		Aves 3	11	296.96	68.80	292.26	67.10	0.000001
		Aves 4	46	1239.51	72.50	825.87	49.80	0.508657
	Reptilia		15	205.90	35.30	110.73	19.50	0.999999
		Reptilia C + T	8	166.91	66.40	159.88	63.20	0.212424
		Reptilia S	7	147.30	54.20	96.46	35.40	0.931465
Mammalia								
	Rodentia		20	374.84	44.30	278.71	33.90	0.999999
	Chiroptera		11	320.96	80.90	315.53	79.60	0.000001
	Marsupialia		3	72.70	79.70	69.58	75.70	0.000001
	Perissodactyla		4	118.78	92.40	118.78	92.40	0.000001
	Artiodactyla		24	404.76	42.30	389.37	40.90	0.997109
		Artiodactyla CCS	11	282.10	66.50	270.16	63.60	0.000001
		Artiodactyla CB	13	390.29	88.90	383.18	87.30	0.000001
	Afroteria/Xenarthra		5	97.01	57.90	85.01	51.20	0.833609
	Carnivora		13	353.49	84.70	324.64	73.10	0.000001
	Primates		25	785.85	84.20	659.82	71.00	0.101152
(all)	(canonical)		19	263.52	32.70	175.98	22.80	0.999999

The data were generated in GeneBee. Two algorithms for alignment (refined alignment, draft source alignment) were applied. The cluster algorithm for unrooted tree with scaled branches had the max/min factor set to 8. The three columns on the left show the taxonomic groups analyzed in hierarchical order. N = number of sequences within the group.

## Supplementary Materials

The following are available online at http://www.mdpi.com/1422-0067/19/9/2557/s1.
