# Supplementary material for: The Phylogeny of Osteopontin—Analysis of the Protein Sequence
Source: Int J Mol Sci. 2018 Aug 28;19(9):2557. doi: 10.3390/ijms19092557 (PMC6164354; doi:10.3390/ijms19092557)
Supplement: Supplementary file 1 [file ijms-19-02557-s001.pdf]

SUPPLEMENT

Table S1: Alignments by taxa. Retrieved sequences were grouped by taxonomic order and aligned with Clustal Omega. The canonical sequence is highlighted in yellow. Common variants are on green, turquoise or blue background. Rare deviations from the consensus sequence have no background color. The putative signal sequence is boxed. 1.1. Primates 1.2. Carnivora 1.3. Artiodactyla (Cervidae and Bovidae) 1.4. Artiodactyla (Camelidae, Suidae and Celacea) 1.5. Perissodactyla 1.6. Afrotheria and Xenarthra 1.7. Marsupialia 1.8. Chiroptera 1.9. various Eutheria 1.10. Lagomorpha and similar orders 1.11. Rodentia 1.12. Reptilia (Crocodilia and Testudines) 1.13 Reptilia (Squamata) 1.14. Aves 1.15. Fish.

```
XM_003790097.3:Otolemur garnettii                -------------MRLAVICFCLLGIAYTLPVKHVDSGSSEEKQFYNKYPDAVATWLKPD
NC_033688.1:Microcebus murinus                   -------------MGIAMICFCLFGIAYALPVKPANSGSSEEKQLHNKYPDAVATWLKPD
XM_012647256.1:Propithecus coquereli             -------------MRIAVICFCLFGIAYALPVKPADSGSSEEKQLYNKYPDAVATWLKPD
NW_007247132.1:Carlito syrichta                  -------------MRIAVICFCLLSLTYALPVKLADSGSSEEKQLYNKYPEAVATWLKPD
XM_002745608.4:Callithrix jacchus                -------------MRIAVICFCLLGIAYALPVKRADSGSSEEKQLYNKYPDAVATWLKPD
XM_003923998.2:Saimiri boliviensis boliviensis   -------------MRIAVICFCLLGIAYALPVKQADSGSSEEKQLYNKYPDAVATWLKPD
XM_012437737.1:Aotus nancymaae                   -------------MRIAVICFCLLGIAYALPVKQTDSGSSEEKQLYNKYPDAVATWLKPD
NW_016107492.1:Cebus capucinus                   -------------MRLAVICFCLLGIAYALPVKQADSGSSEEKQLYNKYPDAVATWLKPD
NW_012116577.1:Colobus angolensis palliatus      MGIVPRSLDKKAHRVQFQLNRIKAKIELPWGSLQLDFLMKTLKELYNKYPDAVATWLKPD
NW_019322877.1:Piliocolobus tephrosceles         -------------MRIAVICFCLLGIAYALPVKQADSGSSEEKQLYNKYPDAVATWLKPD
XM_010366156.1:Rhinopithecus roxellana          -------------MRIAVICFCLLGIAYALPVKQADSGSSEEKQLYNKYPDAVATWLKPD
XM_017895924.1:Rhinopithecus bieti              -------------MRIAVICFCLLGIAYALPVKQADSGSSEEKQLYNKYPDAVATWLKPD
NW_012104043.1:Mandrillus leucophaeus           -------------MRIAVICFCLLGIAYALPVKQADSGSSEEKQLYNKYPDAVATWLKPD
XM_003898941.3:Papio anubis                      -------------MRIAVICFCLLGIAYALPVKQADSGSSEEKQLYNKYPDAVATWLKPD
NC_023648.1:Chlorocebus sabaeus                  -------------MRIAVISFCLLGIAYALPVKQADSGSSEEKQLYNKYPDAVATWLKPD
XM_012040129.1:Cercocebus atys                   -------------MRIAVISFCLLGIAYALPVKQADSGSSEEKQLYNKYPDAVATWLKPD
NM_001261806.2:Macaca mulatta                    -------------MRIAVISFCLLGIAYALPVKQADSGSSEEKQLYNKYPDAVATWLKPD
XM_011737736.1:Macaca nemestrina                 -------------MRIAVISFCLLGIAYALPVKQADSGSSEEKQLYNKYPDAVATWLKPD
XM_005555383.2:Macaca fascicularis               -------------MRIAVISFCLLGIAYALPVKHADSGSSEEKQLYNKYPDAVATWLKPD
XM_012499285.1:Nomascus leucogenys               -------------MRIAVICFCLLGITYALPVKQAGSGSSEEKQLYNKYPDAVATWLKPD
CR859846.1:Pongo abelii                          -------------MRIAVICFCLLGITYALPVKQADSGSSEEKQLYNKYPDAVATWLKPD
NM_001040058.1:Homo sapiens                      -------------MRIAVICFCLLGITCAIPVKQADSGSSEEKQLYNKYPDAVATWLNPD
NC_018428.2:Gorilla gorilla gorilla              -------------MRIAVICFCLLGITYALPVKQADSGSSEEKQLYNKYPDAVATWLNPD
NC_006471.4:Pan troglodytes                      -------------MRIAVICFCLLGITCALPVKQADSGSSEEKQLYNKYPDAVATWLNPD
XM_008963650.1:Pan paniscus                      -------------MRIAVICFCLLGITCALPVKQADSGSSEEKQLYNKYPDAVATWLNPD
                                                                  : * *  :           .   .  *:

XM_003790097.3:Otolemur garnettii                PSQKQNLLAPQ---------------TLPSTSNESHDHMDDEDDD---DHVDSH---ESD
NC_033688.1:Microcebus murinus                   PSQKKNLLAPQNAVSSEETVKFKQQSSFLSKSNEGHDHMEDEGDDDDANHVDSHESVDSD
XM_012647256.1:Propithecus coquereli             PSQKQNLLAPQNAVSSEETDNFKQE-TLPSKSNESHDHVDDVDDEDDEDDGD---HADSH
NW_007247132.1:Carlito syrichta                  PSQKQNLLAPQNAVSSEETDDFKQE-TLPSKSNESHDHMDDVDDD-DGDHVDSQDSIDSD
XM_002745608.4:Callithrix jacchus                PSQKQNLLAPQNAVSSEETDDFKQE-TLPSKSNESHDDMDDVDDDDDDHVDSQD---SN
XM_003923998.2:Saimiri boliviensis boliviensis   PSQKQNLLAPQNAVSSEETDDFKQE-TLPSKSNESHDDMDDVDDEDDDDHVDSQDSVDSK
XM_012437737.1:Aotus nancymaae                   PSQKQNLLAPQNAVSSEETDDFKQE-TLPSKSNESHDDMDDVDDEDDDDHVDSQDSVDSN
NW_016107492.1:Cebus capucinus                   PSQKQNLLAPQNAVSSEETDDFKQE-TLPSKSNESHDDMDDVDDDDDDDHVDSQDSVDSK
NW_012116577.1:Colobus angolensis palliatus      PSQKQNLLAPQNAVSSEETNDFKQE-TLPSKSNESHDHMDDVDDEDDDDHVDSQDSINSN
NW_019322877.1:Piliocolobus tephrosceles         PSQKQNLLAPQNAVSSEETNDFKQE-TLPSKSNESHDHMDDVDDEDDDDHVDSQDSIDSN
XM_010366156.1:Rhinopithecus roxellana          PSQKQNLLAPQNAVSSEETNDFKQE-TLPSKSNESHDHMDDVDDEDDDDHVDSQDSIDSN
XM_017895924.1:Rhinopithecus bieti              PSQKQNLLAPQNAVSSEETNDFKQE-TLPSKSNESHDHMDDVDDEDDDDHVDSQDSIDSN
NW_012104043.1:Mandrillus leucophaeus           PSQKQNLLAPQNAVSSEETNDFKQE-TLPSKSNESHDHMDDVDDEDDDDHVDSQDSIDSN
XM_003898941.3:Papio anubis                      PSQKQNLLAPQNAVSSEETNDFKQE-TLPSKSNESHDHMDDVDDEDDDDHVDSQDSIDSN
NC_023648.1:Chlorocebus sabaeus                  PSQKQNLLAPQNAVSSEETNDFKQE-TLPSKSNESHDHMDDVDDEDDDDHVDSQDSIDSN
XM_012040129.1:Cercocebus atys                   PSQKQNLLAPQNAVSSEETNDFKQE-TLPSKSNESHDHMDDVDDEDDDDHVDSQDSIDSN
NM_001261806.2:Macaca mulatta                    PSQKQNLLAPQNAVSSEETNDFKQE-TLPSKSNESHDHMDDVDDEDDDDHVDSQDSIDSN
XM_011737736.1:Macaca nemestrina                 PSQKQNLLAPQNAVSSEETNDFKQE-TLPSKSNESHDHMDDVDDEDDDDHVDSQDSIDSN
XM_005555383.2:Macaca fascicularis               PSQKQNLLAPQNAVSSEETNDFKQE-TLPSKSNESHDHMDDVDDEDDDDHVDSQDSIDSN
XM_012499285.1:Nomascus leucogenys               PSQKQNLLAPQNAVSSEETNDFKQE-TLPSKSNESHDHMDDVDDEDDDDHVDSQDSIDSN
CR859846.1:Pongo abelii                          PSQKQNLLAPQ---------------TLPSKSNESHDHMDDVDDEDDDDHVDSQDSIDSN
NM_001040058.1:Homo sapiens                      PSQKQNLLAPQNAVSSEETNDFKQE-TLPSKSNESHDHMDDMDDEDDDDHVDSQDSIDSN
NC_018428.2:Gorilla gorilla gorilla              PSQKQNLLAPQNAVSSEETNDFKQE-TLPSKSNESHDHMDDVDDEDDDDHVDSQDSIDSN
NC_006471.4:Pan troglodytes                      PSQKQNLLAPQNAVSSEETNDFKQE-TLPSKSNESHDHMDDVDDEDDDDHVDSQDSIDSN
XM_008963650.1:Pan paniscus                      PSQKQNLLAPQNAVSSEETNDFKQE-TLPSKSNESHDHMDDVDDEDDDDHVDSQDSIDSN
                                                                           :: *.***.**.::* .*:   :. *      *.
```

```
XM_003790097.3:Otolemur garnettii                 DSDDTDNPDDSHHSDESHHSDESDEMVTANFPTDVP-----TPTVPTENI-YDGRGDSIP
NC_033688.1:Microcebus murinus                    DSDEA---------D-SHHSDESEEVAT-QVPPDTPATSVLTPPVPTVDT-HHGRGDSVV
XM_012647256.1:Propithecus coquereli              DSDDVDHPDDSHHSDESHHSDESDEVVT-DFPTDTPITSVFTPPVPTEDT-YDGRGDSVA
NW_007247132.1:Carlito syrichta                   DSDDDDHPDDSHHSDESHHSDESDELVT-DYPTDVPATAVFTPAVPTVDTYYDGRGDSVA
XM_002745608.4:Callithrix jacchus                 DSDEVDHTDDSHQSDESHHSDESDELVT-DFPTDLPATEVFTPPVPTIDT-YDGRGDSVG
XM_003923998.2:Saimiri boliviensis boliviensis    DSDEVDHTDDSHQSDESHHSDESDELVT-DFPTDLPATEVFTPAVPTVDT-YDGRGDSVG
XM_012437737.1:Aotus nancymaae                    DSDEVDHTDDSHQSDESHHSDESDELVT-DFPTDLPATEVFTPAVPTVDT-YDGRGDSVD
NW_016107492.1:Cebus capucinus                    DSDEVDHTDDSHQSDESHHSDESDELVT-DFPTDLPATEVFTPAVPTVDT-YDGRGDSVG
NW_012116577.1:Colobus angolensis palliatus       DSDEVDDTDDSHQSDESHHSDESDELVT-DFPTDLPATEVFTPVVPTVDI-YDGRGDSVA
NW_019322877.1:Piliocolobus tephrosceles          DSDEVDDTDDSHQSDESHHSDESDELVT-DFPTDLPATEVFTPVVPTVDT-YDGRGDSVA
XM_010366156.1:Rhinopithecus roxellana            DSDEVDDTDDSHQSDESHHSDESDELVT-DFPTDLPATEVFTPVVPTVDI-YDGRGDSVA
XM_017895924.1:Rhinopithecus bieti                DSDEVDDTDDSHQSDESHHSDESDELVT-DFPTDLPATEVFTPVVPTVDI-YDGRGDSVA
NW_012104043.1:Mandrillus leucophaeus             DSDEVDDTDDSHQSDESHHSDESDELVT-DFPTDLPATEVFTPVVPTVDI-YDGRGDSVA
XM_003898941.3:Papio anubis                       DSDEVDDTDDSHQSDESHHSDESDELVT-DFPTDLPATEVFTPVVPTVDI-YDGRGDSVA
NC_023648.1:Chlorocebus sabaeus                   DSDEVDDTDDSHQSDESHQSDESDELVT-DFPTDLPATEVFTPVVPTVDI-YDGRGDSVA
XM_012040129.1:Cercocebus atys                    DSDEVDDTDDSHQSDESHHSDESDELVT-DFPTDLPATEVFTPVVPTVDI-YDGRGDSVA
NM_001261806.2:Macaca mulatta                     DSDEVDDTDDSHQSDESHHSDESDELVT-DFPTDLPATEVFTPVVPTVDI-YDGRGDSVA
XM_011737736.1:Macaca nemestrina                  DSDEVDDTDDSHQSDESHHSDESDELVT-DFPTDLPATEVFTPVVPTVDI-YDGRGDSVA
XM_005555383.2:Macaca fascicularis                DSDEVDDTDDSHQSDESHHSDESDELVT-DFPTDLPATEVFTPVVPTVDI-YDGRGDSVA
XM_012499285.1:Nomascus leucogenys                DSDDVDDTDDSHQSDESHHSDESDELVT-DFPTDLPATEVFTPVVPTVDT-YDGRGDSVV
CR859846.1:Pongo abelii                           DSDDVDDTDDSHQSDESHHSDESDELVT-DFPTDLPATEVFTPVVPTVDT-YDGRGDSVV
NM_001040058.1:Homo sapiens                       DSDDVDDTDDSHQSDESHHSDESDELVT-DFPTDLPATEVFTPVVPTVDT-YDGRGDSVV
NC_018428.2:Gorilla gorilla gorilla               DSDDVDDTDDSHQSDESHHSDESDELVT-DFPTDLPATEVFTPVVPTVDT-YDGRGDSVV
NC_006471.4:Pan troglodytes                       DSDDVDDTDDSHQSDESHHSDESDELVT-DFPTDLPATEVFTPVVPTVDT-YDGRGDSVV
XM_008963650.1:Pan paniscus                       DSDDVDDTDDSHQSDESHHSDESDELVT-DFPTDLPATEVFTPVVPTVDT-YDGRGDSVV
                                                  ***:           :**:****:*:.* : * * *     ** *** :  :.*****:

XM_003790097.3:Otolemur garnettii                 YGLRSKSKKFRRLDIQIPDETQEDFTSYVESEELGHGPKVIAFNQGLKVPSDLDSHETSH
NC_033688.1:Microcebus murinus                    RGLKSKSKKFPSHDIQFPDSVEEDLASHMESEDLDGPHRAVPIAQRLNVLFAQENLGKDS
XM_012647256.1:Propithecus coquereli              YGLRSKSKKFRRHDVQFPDSTEEDPTSRMESEDSDDARKAVPIAHRLNVPFAWESLGKDS
NW_007247132.1:Carlito syrichta                   YGLRSKAKMLHRPNIQYPDVTDEDLTSHMESEELNGAFKAIPVAQGLNVPSDWDSHGKDS
XM_002745608.4:Callithrix jacchus                 YGLRSKSKKFRRSDIQYPDATEEDITSHVESEELNGAYKAIPVAQSLNVPSDWDSHGKDS
XM_003923998.2:Saimiri boliviensis boliviensis    YGLRSKSKKFRRSDIQYPDAT-EDITSHMESEELKDAYKAVPVAQSLNVPSDWDSHGKDS
XM_012437737.1:Aotus nancymaae                    YGLRSKSKKFRRSDIQYPDATQEDITSHMESEELNGAYKAIPVAQSLNVPSDWDSHGKDS
NW_016107492.1:Cebus capucinus                    YGLRSKSKKFRRSDIQYPDATEEDITSHVESEELNGAYKAVPVAQSLNVPSDWDSHGKDS
NW_012116577.1:Colobus angolensis palliatus       YGLRSKSKKFRRPDIQYPDDTDEDITSHMESKELNGAYKAIPVAQGLNAPFDWDSRGKDS
NW_019322877.1:Piliocolobus tephrosceles          YGLRSKSKKFRRPDIQYPDATDEDITSHMESKELNGAYKAIPVAQGLNAPSDWDSRGKDS
XM_010366156.1:Rhinopithecus roxellana            YGLRSKSKKFRRPDFQYPDATDEDITSHMESKELNGAYKAIPVAQGLNAPSDWDSRGKDS
XM_017895924.1:Rhinopithecus bieti                YGLRSKSKKFRRPDFQYPDATDEDITSHMESKELNGAYKAIPVAQGLNAPSDWDSRGKDS
NW_012104043.1:Mandrillus leucophaeus             YGLRSKSKKFRRPDIQYPDATDEDITSYVESEELNGAYKAIPVVQGLNVPSDWDSRGKDS
XM_003898941.3:Papio anubis                       YGLRSKSKKFRRPDIQYPDATDEDITSHVESEELNGAYKAIPVVQGLNVPSDWDSRGKDS
NC_023648.1:Chlorocebus sabaeus                   YGLRSKSKKFRRPDIQYPDATDEDITSHVESEELNGAYKAIPVAQGLNVPSDWDSRGKDS
XM_012040129.1:Cercocebus atys                    YGLRSKSKKFRRPDIQYPDATDEDITSHVESEELNGAYKAIPVVQGLNVPSDWDSRGKDS
NM_001261806.2:Macaca mulatta                     YGLRSKSKKFRRPDIQYPDATDEDITSHVESEELNGAYKAIPVVQGLNVPSDWDSRGKDS
XM_011737736.1:Macaca nemestrina                  YGLRSKSKKFRRPDIQYPDATDEDITSHVESEELNGAYKAIPVVQGLNVPSDWDSRGKDS
XM_005555383.2:Macaca fascicularis                YGLRSKSKKFRRPDIQYPDATDEDITSHVESEELNGAYKAIPVVQGLNVPSDWDSRGKDS
XM_012499285.1:Nomascus leucogenys                YGLRSKSKKFRRPDIQYPDATDEDITSHMESEELNGAYKAIPVAQDLNAPSDWDSRGKDN
CR859846.1:Pongo abelii                           YGLRSKSKKFRRPDIQYPDATDEDITSHMESEELNGAYKAIPVAQDLNTPSDWDSHGKDS
NM_001040058.1:Homo sapiens                       YGLRSKSKKFRRPDIQYPDATDEDITSHMESEELNGAYKAIPVAQDLNAPSDWDSRGKDS
NC_018428.2:Gorilla gorilla gorilla               YGLRSKSKKFRRPDIQYPDATDEDITSHMESEELNGAYKAIPVAQDLNAPSDWDSRGKDS
NC_006471.4:Pan troglodytes                       YGLRSKSKKFRRPDIQYPDAIDEDITSHMESEELNGAYKAIPVAQDLNAPSDWDSRGKDS
XM_008963650.1:Pan paniscus                       YGLRSKSKKFRRPDIQYPDAIDEDITSHMESEELNGAYKAIPVAQDLNAPSDWDSRGKDS
                                                   **:**:* :   :.* **   ** :* :**::     :.: . : *:.    :.  ..
```

```
XM_003790097.3:Otolemur garnettii                   -------DLSTE---------------THSKEPSDMIVSQEHSHVSLEVTSHE-------
NC_033688.1:Microcebus murinus                      RETSQMDDPSVEAHSHEQSRLRKRKADASSNEHSDVIDSQGHS------HSHEFLSQEDR
XM_012647256.1:Propithecus coquereli                HETSQMDDQSVETHSHEQSRLHKRKADDASNEHSDMIDSQERS------HSREFHSQEDR
NW_007247132.1:Carlito syrichta                     HETSQQDDQSAETHSHEQSRLYKRRASDESNEHSDMINSQETSKVSHEIHSHEVHSHEDL
XM_002745608.4:Callithrix jacchus                   HEMSQLDDQSAETHSHEQSRLHKRKASDESNEHSDVIDSQEVSKVSREFHSHEFHSQEDL
XM_003923998.2:Saimiri boliviensis boliviensis      HEMSQLDDQSAETHSHEKSRLHKRKASDESNEHSEVVDSQEVSKVSREFHSHEFHSQEDM
XM_012437737.1:Aotus nancymaae                      HEMSQLDDQSAETHSHEQSRLHKRKASEESNEHSDVIDSQEVSKVSREFHSHEFHSQEDM
NW_016107492.1:Cebus capucinus                      HEMSQLDDQSAETYSHEQSRLHKRKASDESNEHSDVVDSQEVSKVSREFHSHEFHSQEDM
NW_012116577.1:Colobus angolensis palliatus         HETSQLDDHSAETHSHKQSRLYKRKASDDSNEHSDVIDSQERSKVSREFHSHEFHSHEDM
NW_019322877.1:Piliocolobus tephrosceles            HETSQLDDHSAETHSHKQSRLYKRKASDDSNEHSDVIDSQERSKVSREFHSHEFHSHEDM
XM_010366156.1:Rhinopithecus roxellana              HETSQLDDHSAETHSHKQSRLYKRKASDDSNEHSDVIDSQERSKVSREFHSHEFHSHEDM
XM_017895924.1:Rhinopithecus bieti                  HETSQLDDHSAETHSHKQSRLYKRKASDDSNEHSDVIDSQERSKVSREFHSHEFHSHEDM
NW_012104043.1:Mandrillus leucophaeus               HETSQLDDHSAETHSHKHSRLYKRKASDDSNEHSDVIDSQERSKISREFHSHEFHSHEDM
XM_003898941.3:Papio anubis                         HEMSQLDDHSAETHSHKHSRLYKRKASDDSNEHSDVIDSQERSKISHEFHSHEFHSHEDM
NC_023648.1:Chlorocebus sabaeus                     HETSQLDDHSAETHSHKHSRLYKRKASDDSNEHSDVIDSQERSKISREFHSHEFHSHEDM
XM_012040129.1:Cercocebus atys                      HETSQLDDHSAETHSHKHSRLYKRKASDDSNEHSDVIDSQERSKISREFHSHEFHSHEDM
NM_001261806.2:Macaca mulatta                       HETSQLDDHSAETHSHKHSRLYKRKASDDSNEHSDVIDSQERSKISHEFHSHEFHSHEDM
XM_011737736.1:Macaca nemestrina                    HETSQLDDHSAETHSHKHSRLYKRKASDDSNEHSDVIDSQERSKISHEFHSHEFHSHEDM
XM_005555383.2:Macaca fascicularis                  HETSQLDDHSAETHSHKHSRLYKRKASDDSNEHSDVIDSQERSKISHEFHSHEFHSHEDM
XM_012499285.1:Nomascus leucogenys                  HETSQLDDQSAETHSHKQSRLYKRKP-----------------QVSHEFHSHEFHSHEDT
CR859846.1:Pongo abelii                             HEMSQLDDQSAETHSHKKSRLYKRKASDESNEHSDVIDSQELSKVSHEFHSHEFHSHEDM
NM_001040058.1:Homo sapiens                         YETSQLDDQSAETHSHKQSRLYKRANDESNEHSDVIDSQELSKVSREFHSHEFHSHEDM
NC_018428.2:Gorilla gorilla gorilla                 YETSQLDDQSAETHSHKQSRLYKRKASDESNEHSDVIDSQELSKVSREFHSHEFHSHEDM
NC_006471.4:Pan troglodytes                         YETSQLDDQSAETHSHKQSRLYKRKASDESNEHSDVIDSKELSKVSREFHSHEFHSQGDM
XM_008963650.1:Pan paniscus                         YEMSQLDDQSAETHSHKQSRLYKRKASDESNEHSDVIDSKELSKVSREFHSHEFHSQGDM
                                                           * *.*                                      *:*

XM_003790097.3:Otolemur garnettii                   ---DLESKEEDKLLKFRVSHELDSASSEVN
NC_033688.1:Microcebus murinus                      LVPDPQE-----------------------
XM_012647256.1:Propithecus coquereli                LVPDPKSKEEDKHLKFRISHELDSASSEVN
NW_007247132.1:Carlito syrichta                     LVLDPKSKEEDTHLKFRISHELDSTSSEVN
XM_002745608.4:Callithrix jacchus                   LVVDPKSTEEDKHLKFRISHELDSASSEVN
XM_003923998.2:Saimiri boliviensis boliviensis      LVVDPKSKEEDKHLKFRISHELDSASSEVN
XM_012437737.1:Aotus nancymaae                      LVVDPKSKEEDKHLKFRISHELDSASSEVN
NW_016107492.1:Cebus capucinus                      LVVDPKSKEEDKHLKFRISHELDSASSEVN
NW_012116577.1:Colobus angolensis palliatus         LIVDPKSKEEDKHLKFRISHELDSASSEVN
NW_019322877.1:Piliocolobus tephrosceles            LIVEPKSKEEDKHLKFRISHELDSASSEVN
XM_010366156.1:Rhinopithecus roxellana              LIVDPKSKEEDKHLKFRISHELDSASSEVN
XM_017895924.1:Rhinopithecus bieti                  LIVDPKSKEEDKHLKFRISHELDSASSEVN
NW_012104043.1:Mandrillus leucophaeus               LVVDSKSKEEDKHLKFRISHELDSASSEVN
XM_003898941.3:Papio anubis                         LVVDPKSKEEDKHLKFRISHELDSASSEVN
NC_023648.1:Chlorocebus sabaeus                     LVVDPKSKEEDKHLKFRISHELDSASSEVN
XM_012040129.1:Cercocebus atys                      LVVDPKSKEEDKHLKFRISHELDSASSEVN
NM_001261806.2:Macaca mulatta                       LVVDPKSKEEDKHLKFRISHELDSASSEVN
XM_011737736.1:Macaca nemestrina                    LVVDPKSKEEDKHLKFRISHELDSASSEVN
XM_005555383.2:Macaca fascicularis                  LVVDPKSKEEDKHLKFRISHELDSASSEVN
XM_012499285.1:Nomascus leucogenys                  LVVDPKSKEEDKHLKFRISHELDSASSEVN
CR859846.1:Pongo abelii                             LVVDPKSKEEDKHLKFRISHELDSASSEVN
NM_001040058.1:Homo sapiens                         LVVDPKSKEEDKHLKFRISHELDSASSEVN
NC_018428.2:Gorilla gorilla gorilla                 LVVDPKSKEEDKHLKFRISHELDSASSEVN
NC_006471.4:Pan troglodytes                         LVVDPKSKEEDKHLKFRISHELDSASSEVN
XM_008963650.1:Pan paniscus                         LVVDPKSKEEDKHLKFRISHELDSASSEVN
                                                       : :.
```

Table S1.1: Osteopontin in Primates. As alternative splicing of exons 4 or 5 has been shown to occur in humans, the respective splice junctions are marked by vertical lines. Of note, full-length sequences for *Otolemur garnettii* or *Pongo abelii* were not found; there is no experimental evidence that osteopontin is actually spliced in these animals. The available *Colobus angolensis palliates* sequence likely reflects transcription or translation from an alternative start site.

```
XM_003985184.5:Felis catus                  MRIAVICFCLLGIAYAIPIKQTDSGSSEEKQLYNKYPVAVATWPKPDPSQKQTFLALQNA
XM_015073631.1:Acinonyx jubatus             MRIAVICFCLLGIAYTIPIKQTDSGSSEEKQLYNKYPGAVATWPKPDPSQKQTFLALQNA
XM_019464775.1:Panthera pardus              MRIAVICFCLLGIAYAIPIKQTDSGSSEEKQLYNKYPGAVATWPKPDPSQKQTFLALQNA
XM_007074695.2:Panther tigris               MRIAVICFCLLGIAYAIPIKQTDSGSSEEKQLYNKYPGAVATWPKPDPSQKQTFLALQNA
XM_003434024.4:Canis lupus familiaris       MRIAVICFCLLGIAYAIPIKHADSGSSEEKQLYNKYPGAVATWLKPDPSQKQTFLALQNA
XM_004756106.2:Mustela putorius furo        MKIAVICFCLLGIAYAIPIKQADSGSSEEK---------------------------QNT
XM_002913580.3:Ailuropoda melanoleuca       MRIAVICFCLLGIAFAIPIKQPDSGSSEEKQLYHKYPGAVATWLKPDPSQKQTFLALQNT
XM_008707124.1:Ursus maritimus              MRIAVICFCLLGVAFAIPIKQPDSGSSEEKQLYHKYPGAVATWLKPDPSQKQTFLALQNT
NW_019154087.1:Enhydra lutris kenyoni       MKIAVICFCLLGIAYAIPIKQADSGSSEEKQLYHKYPGAVATWLKPDPSQKQTFLALQNT
NW_004450264.1:Odobenus rosmarus divergens  MRLAVICFCLLGIAYAIPIKQADSGSSEEKQLYNKYPGAVATWLKPDPSQKQTFLALQNT
XM_021702336.1:Neomonachus schauinslandi    MRLAVICFCLLGIAYAIPIKQADSGSSEEK---------------------------QNT
NW_006383049.1:Leptonychotes weddellii      MRLAVICFCLLGIAYAIPIKQADSGSSEEKQLYNKYPGAVATWLKPDPSQKQTFLALQNT
                                                  ******:*::****: ********                           **:

XM_003985184.5:Felis catus                  VLSEETDDFKQKTLASKSNESHDVDDEDDEDDVDSQDSVDSHDTDDDSNQSDESDELVTD
XM_015073631.1:Acinonyx jubatus             VLSEETDDFKQKALASKSNESHDVDDEDDEDDVDSQDSVDSHDTDDDSNQSDESDELVTD
XM_019464775.1:Panthera pardus              VLSEETDDFKQKTLASKSNESHDVDDEDDGDDVDSQDSVDSHDTDDDSNQSDESDELVTD
XM_007074695.2:Panther tigris               VLSEETDDFKQKTLASKSNESHDVDDEDDGDDVDSQDSVDSHDTDDDSNQSDESDELVTD
XM_003434024.4:Canis lupus familiaris       VLTEETDDFKQKTFSSKSNESHDDVDEDDGDDVDSQDSVDSNDLDDDSNESDESDELVTD
XM_004756106.2:Mustela putorius furo        VLSQENDDFQQKTLSSKSDESHEDVDDDDRDDVDSQDSVDSDDVDDDSKQSDESDEVVTD
XM_002913580.3:Ailuropoda melanoleuca       VLSQETDDFRQKTLSSKSNESHDDVDEDDRDDVDSQDSDDSNDGDDDSNQSDESDELVTD
XM_008707124.1:Ursus maritimus              VLSQETDDFQQKTLSSKSDESHDDVDEDDGDDVDSQDSVDSNDVDDNSNQSDESDELVTD
NW_019154087.1:Enhydra lutris kenyoni       VLSQENDDFQQKTLSSKSDESHEDVDDDDRDDVDSQDSVDSNDVDDDSNQSDESDEVVTD
NW_004450264.1:Odobenus rosmarus divergens  VLSQENDDFQQKTLSSKSNESHEDVDEDDGDDVDSQDSIDSNDVDDDSNQSDESDELVTD
XM_021702336.1:Neomonachus schauinslandi    VLSQENDNFQQKTLSSKSNESHEDVDEDDGDDVDSQDSIDSNDVDDDSNQSDESDELVTD
NW_006383049.1:Leptonychotes weddellii      VLSQENDNFQQKTLSSKSNESHEDVDEDDGDDVDSQDSIDSNDVDDDSNQSDESDELVTD
                                            **::*.*:*:**:::***:***:  *:** ******** **.* **:*::******:***

XM_003985184.5:Felis catus                  FPTDVPATQFFTPAVPTRDSYDGRGDSVAYGLRSKSKKSHRYEDQYPDSTEEDFTSLVKS
XM_015073631.1:Acinonyx jubatus             FPTDVPATQFFTPAVPTRDSYDGRGDSVAYGLRSKSKKSHRYEDQYPDSTEEDFTSLVKS
XM_019464775.1:Panthera pardus              FPTDVPATQFFTPAVPTRDSYDGRGDSVAYGLRSKSKKSHRYEDQYPDSTEEDFTSLVKS
XM_007074695.2:Panther tigris               FPTDVPATQFFTPAVPTRDSYDGRGDSVAYGLRSKSKKSHRYEDQYPDSTEEDFTSLVKS
XM_003434024.4:Canis lupus familiaris       FPTDIPATQLFTPAVPTRGSYDGRGDSVAYGLRSKSKKSHKYEVQYPDSTEEDFTSLVKS
XM_004756106.2:Mustela putorius furo        FPTDIPATQFFTPSVPTRDSNDGRGDSMAHGLRSRSKMSHTYEVQYPDSTEEDLTSLVKS
XM_002913580.3:Ailuropoda melanoleuca       FPTDFPATQFFTPAVPTRDSNDGRGDSVAYGLRSRSKKSHRHEVQYPDSTEEDLTSLVKS
XM_008707124.1:Ursus maritimus              FPTDFPATQFFTPAVPTRDSNDGRGDSVAYGLRSRSKKSHRYEVQYPDSTEEDLTSLVKS
NW_019154087.1:Enhydra lutris kenyoni       FPTDIPATQFFTPAVPTRDSNDGRGDSVAHGLRSRSKKSHTYEVQYPDSTEEDLTSLVKS
NW_004450264.1:Odobenus rosmarus divergens  FPTDIPATQFFTPAVPTRDSNDGRGDSVAHGLRSRSKKSYRYEVQYPDSTEEDLTSLVKS
XM_021702336.1:Neomonachus schauinslandi    FPTDIPATQFFTPAVPTRDSNDGRGDSVAHGLRSRSKKSHRYEVQYPDSTEEDLTSLVKS
NW_006383049.1:Leptonychotes weddellii      FPTDIPATQFFTPAVPTRDSNNGRGDSVAHGLRSRSKKSHRYEVQYPDSTEEDLTSLVKS
                                            ****.****:***:****.* :*****:*:****:** *: :* *********:******
```

```
XM_003985184.5:Felis catus                   QSMEDDFNAVLLSHTVRRSPDRD-SHVKDSQETSQVDDHSMETKSRKHSKEYKLKASDEN
XM_015073631.1:Acinonyx jubatus              QSMEDDFNAVLLSHTVRRSPDRD-SHVKDSQETSQVDDHSMETKSRKHSKEYKLKASDEN
XM_019464775.1:Panthera pardus               QSTKDDFNAVLLSHTVRRSPDRDNSHVKDSQETSQVDDHSMETKSRKHSKEYKLKASDEN
XM_007074695.2:Panther tigris                QSTKDDFNAVLLSHTVRRSPDRD-SHVKDSQETSQVDDHSMETKSRKHSKEYKLKASDEN
XM_003434024.4:Canis lupus familiaris        ASMEDDFNAVLLSRTVRGTSDRD-SHAKDSQETSQLDDHSMETKGRKHSQEYKLRASDES
XM_004756106.2:Mustela putorius furo         ESTEEDFNADLLSQTVQGTSDRD-SHARHSQETSQMDDHSMETKDRRHTREYKLRASDES
XM_002913580.3:Ailuropoda melanoleuca        GSTEDDFNAVLLSQTVRGTSDGD-SHAKDSQETSQLDDHSMETKSRRHTREYKLRASDES
XM_008707124.1:Ursus maritimus               GSMEDDFNAVLLSQTVRGTSDGD-SHAKDSQETSQLDDHSMETKSRRHTREYKLRASDES
NW_019154087.1:Enhydra lutris kenyoni        ESTEDDFNAVLLSQTVRGTSDGD-SHARYSQEASQMDDHSMETKSRRYTREYKLRASDES
NW_004450264.1:Odobenus rosmarus divergens   ESTEDDFNAVLLSRTVRGTSDGD-SHAKDSQETSQLDDHSMETKSHKHAREYKLRASDES
XM_021702336.1:Neomonachus schauinslandi     ESMEDDFNAVLLSQTVRGTSDGD-SHAKDSQETSQPDDHSMETKSHKHAREYKLRASDES
NW_006383049.1:Leptonychotes weddellii       ESMEDDFNAVLLSQTVRGTSDGD-SHAKDSQETSQPDDHSMETKSHKHAREYKLRASDES
                                              * ::**** ***:**: : * * **.: ***:** ********.:::::****:****.

XM_003985184.5:Felis catus                   NKHSHEIGSQESSDISSELVGQTVQSNEKELVQHPESEEQDKHLKFRVSHELDSASSEVN
XM_015073631.1:Acinonyx jubatus              NKHSHEIGSQESSDVSSELVGQTVQSNEKELVQHPESEEQDKHLKFRVSHELDSASSEVN
XM_019464775.1:Panthera pardus               NKHSHEIGSQESSDVSSELVGQTVQSSEKELDQHAESEEQDKHLKFRVSHELDSASSEVN
XM_007074695.2:Panther tigris                NKHSHEIGSQESSDVSSELVGQTVQSSEKELDQHAESEEQDKHLKFRVSHELDSASSEVN
XM_003434024.4:Canis lupus familiaris        NMHSHEIGSQENSEVSSELVSQLSQSHEKELIVDSKSEEEDKHLKFHVSHELDSASSEIN
XM_004756106.2:Mustela putorius furo         SKHSQEIGSQENSEVSSELSIYIVQSHENEQVLDSGSVEEDKHLKFHISHELDNASTEVN
XM_002913580.3:Ailuropoda melanoleuca        DRHSHEIDSQENSEVSSELVSQIVQSHEKGLALDSKSEE-DKHLKFRTSHEFDSASSEVN
XM_008707124.1:Ursus maritimus               DRHSHEIDSQENSEVSSELVSQIVQSHEKGLVLDSKSEE-DKHLKFHTSHELDSASSEVN
NW_019154087.1:Enhydra lutris kenyoni        SKHSHEIGSQENSEVSSELSSQIVQSHENEQVLDSGSVEEDKHLKLRISHELDSASTEVN
NW_004450264.1:Odobenus rosmarus divergens   NKHSHEIGSQENSEVSSELVSQIVQSHEKERVLDSKSVEEDKHLKFRISHELDSASSEVN
XM_021702336.1:Neomonachus schauinslandi     NKHSHEIGSQENSEVSSELVSQIVQSHEKERVLDSKSVEEDKHLKFHISHELDSASSEVN
NW_006383049.1:Leptonychotes weddellii       NKHSHEIGSQENSEVSSELVSQIVQSHEKERVLDSKSVEEDKHLKFRISHELDSASSEVN
                                             . **:**.***.*::****     ** *:    .    *:*****:: ***:*.** *:*
```

Table S1.2: Osteopontin in Carnivora: As alternative splicing of exons 4 or 5 has only been shown to occur in humans, the sequences for *Mustela putorius furo* and *Neomonachus schauinslandi* are likely not accurate reflections of the full transcripts.

```
HQ161904.1:Cervus nippon                        MRIAVICCCFLGIASALPVQLTNSGSSEEA-AYNKYPDAVATWLKPDPSQKQTFLTPQNS
MKHE01000017.1:Cervus elaphus hippelaphus       -----MAKRLMTHLPAPRVQLTNSGSSEEKQLNNKYPDAVATWLKPDPSQKQTFLTPQNS
XM_020876412.1:Odocoileus virginianus texanus   -------------------------------LNNKYPDAVATWLKPDPSQKQTFLTPQNS
S45840.1:Bos taurus                             MRIAVICFCLLGIASALPVKPTSSGSSEEKQLNNKYPDAVATWLKPDPSQKQTFLAPQNS
DQ295062.1:Bubalus bubalis                      MRIAVICFCLLGIASALPVKPTSSGSSEEKQLNNKYPDAVATWLKPDPSQKQTFLAPQNS
M66236.1:Bos                                    MRIAVICFCLLGIASALPVKPTSSGSSEEKQLNNKYPDAVAIWLKPDPSQKQTFLTPQNS
XM_010860191.1:Bison bison bison                MRIAVICFCLLGIASALPVKPTSSGSSEEKQLNNKYPDAVATWLKPDPSQKQTFLAPQNS
JH881339.1:Bos grunniens                        MRIAVICFCLLGIASALPVKPTSSGSSEEKQLNNKYPDAVATWLKPDPSQKQTFLAPQNS
NW_005393912.1:Bos mutus                        MRIAVICFCLLGIASALPVKPTSSGSSEEKQLNNKYPDAVATWLKPDPSQKQTFLAPQNS
KU985440.1:Bos indicus                          MRIAVICFCLLGIASALPVKPTSSGSSEEKQLNNKYPDAVATWLKPDPSQKQTFLAPQNS
XM_005972457.1:Pantholops hodgsonii             MRIAVICFCLLGIASALPVKPTSSGSSEEKQLNNKYPDAVATWLKPDPSQKQTFLAPQNS
AF152416.1:Ovis aries                           MRIAVICFCLLGIASALPVKPTSSGSSEEKQLNNKYPDAVATWLKPDPSQKQTFLEPQNS
KJ789112.1:Capra hircus                         MRIAVICFCLLGIASALPVKPTSSGSSEEKLLNNKYPDAVATWLKPDPSQKQTFLEPQNS
                                                                                 ******** ************* ****

HQ161904.1:Cervus nippon                        VSSEETDDNKQNTLPSKSNESPEQTDDLDDDDDENSQDVTFNDSDDTDNTDDSDHSNESH
MKHE01000017.1:Cervus elaphus hippelaphus       VSSEETDDNKQNTLPSKSNESPEQTDDLDDDDDENSQDVTSNDSDDTDNTDDSDHSNESH
XM_020876412.1:Odocoileus virginianus texanus   VSSEETDDNKQNTLPSKSNESPEQIDDLDDDDDENSQDVTSNDSDDTDNTDDSDHSNESH
S45840.1:Bos taurus                             VSSEETDDNKQNTLPSKSNEDPEQTDDLDDDD-DNSQDVNSNDSDDAETTDDPDHSDESH
DQ295062.1:Bubalus bubalis                      VSSEETDDNKQNTLLSKSNESPEQTDDLDDDDDDNSQDVNSNDSDDADTTDDPDHSDESH
M66236.1:Bos                                    VSSEETDDNKQNTLPSKSNESPEQTDDLDDDD-DNSQDVNSNDSDDAETTDDPDHSDESH
XM_010860191.1:Bison bison bison                VSSEETDDNKQNTLPSKSNESPEQTDDLDDDD-DNSQDVNSNDSDDAETTDDPDHSDESH
JH881339.1:Bos grunniens                        VSSEETDDNKQNTLPSKSNESPEQTDDLDDDD-DNSQDVNSNDSDDAETTDDPDHSDESH
NW_005393912.1:Bos mutus                        VSSEETDDNKQNTLPSKSNESPEQTDDLDDDD-DNSQDVNSNDSDDAETTDDPDHSDESH
KU985440.1:Bos indicus                          VSSEETDDNKQNTLPSKSNESPEQTDDLDDDD-DNSQDVNSNDSDDAETTDDPDHSDESH
XM_005972457.1:Pantholops hodgsonii             VSSEETDDNKQNTLPSKSNESPEQTDDLDDDDDENSQEVNSDDSDDA-NPDDSDHSNESH
AF152416.1:Ovis aries                           VSSEETDDNKQNTLPSKSNESPEQTDDLDDDD-ENSQEVNSDDSDDAETPDDSDHSNESH
KJ789112.1:Capra hircus                         VSSEETDDNKQNTLPSKSNESPEQTDDLDDDD-ENSQEVNSDDSDDA-NPDDSDHSNESH
                                                ************** *****.*** ******   :***:*. :****   . ** ***:***

HQ161904.1:cervus_nippon                        HSDESDEVDFPTDIPTTAVSTPPIPTENTNDGRGDSVAYGLKSKSKKFRRSSVQSPDATE
MKHE01000017.1:Cervus elaphus hippelaphus       HSDESDEVDFPTDIPTTAVSTPPIPTENTNDGRGDSVAYGLKSKSKKFRRSSVQSPDATE
XM_020876412.1:Odocoileus virginianus texanus   HSDESDEADFPTDVPTTAVSTPPIPTENTNDGRGDSVAYGLKSKSKKFRRSSIQSPDATE
S45840.1:Bos taurus                             HSDESHQVHFPTDIPTIAVFTPFIPTESANDGRGDRCGLRTEVKIIEVPPINVQSPDATE
DQ295062.1:Bubalus bubalis                      HSDESDEADFPTDIPAIAVFTPFIPTESTNDGRGDSVAYGLKSRSKKFRRSNVQSPDATE
M66236.1:Bos                                    HSDESDEVDFPTDIPTIAVFTPFIPTESANDGRGDSVAYGLKSRSKKFRRSNVQSPDATE
XM_010860191.1:Bison bison bison                HSDESDEVDFPTDIPTIAVFTPFIPTESANDGRGDSVAYGLKSRSKKFRRSNVQSPDATE
JH881339.1:Bos grunniens                        HSDESDEVDFPTDIPTIAVFTPFIPTESANDGRGDSVAYGLKSRSKKFRRSNVQSPDATE
NW_005393912.1:Bos mutus                        HSDESDEVDFPTDIPTIAVFTPFIPTESANDGRGDSVAYGLKSRSKKFRRSNVQSPDATE
KU985440.1:Bos indicus                          HSDESDEVDFPTDIPTIAVFTPFIPTESANDGRGDSVAYGLKSRSKKFRRSNVQSPDATE
XM_005972457.1:Pantholops hodgsonii             HSDESDEADFPTDIPTIAVFTPPFPTESTNDGRGDSVAYGLKSKSKKFRRSNIESPDATE
AF152416.1:Ovis aries                           HSDESDEADFPTDIPTIAVFTPPFPTESTNDGRGDSVAYGLKSKSKKFRRSNVESPDATE
KJ789112.1:Capra hircus                         HSDESDEADFPTDIPTIAVFTPPFPTESTNDGRGDSVAYGLKSKSKKFRRSNVESPDATE
                                                *****.:..****:*: ** ** :***.:******    .    : :   :.    .::******
```

```
HQ161904.1:Cervus nippon                            EDFTSHVESEEMHDAPKKTSQLNDHSKETNSNELSKELKPKAKDESSKHSDVIESQENSK
MKHE01000017.1:Cervus elaphus hippelaphus           EDFTSHVESEEMHDAPKKTSQLNDHSKETNSNELSKELKPKAKDESSKHSDVIESQENSK
XM_020876412.1:Odocoileus virginianus texanus       EDFSSHVESEEMHDAPKKTSQLNDHSKETNSSELSKELKPKAKDESNKHSDVIESQENSK
S45840.1:Bos taurus                                 EDFTSHIESERCM-STKKTSRLTDHSKETNRCELSKELMPKAKDK-NKHSNLIESQENSK
DQ295062.1:Bubalus bubalis                          EDFTSHIESEEMHDAPKKTSQLTDHSKETNSRELSKELTPKAKDKNNKHSNLIESQENSK
M66236.1:Bos                                        EDFTSHIESEEMHDAPKKTSQLTDHSKETNSSELSKELTPKAKDK-NKHSNLIESQENSK
XM_010860191.1:Bison bison bison                    EDFTSHIESEEMHDAPKKTSQLTDHSKETNSSELSKELTPKAKDK-NKHSNLIESQENSK
JH881339.1:Bos grunniens                            EDFTSHIESEEMHDAPKKTSQLTDHSKETNSSELSKELTPKAKDK-NKHSNLIESQENSK
NW_005393912.1:Bos mutus                            EDFTSHIESEEMHDAPKKTSQLTDHSKETNSSELSKELTPKAKDK-NKHSNLIESQENSK
KU985440.1:Bos indicus                              EDFTSHIESEEMHDAPKKTSQLTDHSKETNSSELSKELTPKAKDK-NKHSNLIESQENSK
XM_005972457.1:Pantholops hodgsonii                 EDFTSHIESEEMHDAPKKTSQLTDHSEETNSNELSKELMPKAKDE-SKHSNLIESQENSK
AF152416.1:Ovis aries                               EDFTSHIESEEMHDAPKKTSQLTDHSEETNSDELPKELTPKAKEE-SKHSNRIESQENSK
KJ789112.1:Capra hircus                             EDFTSHIESEEMHDAPKKTSQLTDHSEETNSDELSKELTPKAQEE-SKHSNRIESQENSK
                                                    ***:**:***.   : ****:*.***:***  ** *** ***::: .***: ********

HQ161904.1:Cervus nippon                            VSQEFHSLEEKLDLDHKSE-EDKHLKIRISHELDSTSSEVN
MKHE01000017.1:Cervus elaphus hippelaphus           VSQEFHSLEDKLDLDHKSE-EDKHLKIRISHELDSTSSE--
XM_020876412.1:Odocoileus virginianus texanus       VSQEFHSLEDKLDLDHRSEEEDKHLKIRISHELDSTSSEVN
S45840.1:Bos taurus                                 LSQEFHSLEDKLDLDHKSE-EDKHLKIRISHELDSASSEVN
DQ295062.1:Bubalus bubalis                          LSQEFHSLEDKLDLDHKSE-EDKHLKIRISHELDSASSEVN
M66236.1:Bos                                        LSQEFHSLEDKLDLDHKSE-EDKHLKIRISHELDSASSEVN
XM_010860191.1:Bison bison bison                    LSQEFHSLEDKLDLDHKSE-EDKHLKIRISHELDSASSEVN
JH881339.1:Bos grunniens                            LSQEFHSLEDKLDLDHKSE-EDKHLKIRISHELDSASSEVN
NW_005393912.1:Bos mutus                            LSQEFHSLEDKLDLDHKSE-EDKHLKIRISHELDSASSEVN
KU985440.1:Bos indicus                              LSQEFHSLEDKLDLDHKSE-EDKHLKIRISHELDSASSE-N
XM_005972457.1:Pantholops hodgsonii                 LSQEFHSLEDKLDLDHKSE-EDERLKIRISHELDSASSEVN
AF152416.1:Ovis aries                               LSQEFHSLEDKLDLDHKSE-EDKRLKIRISHELDSVSSEVN
KJ789112.1:Capra hircus                             LSQEFHSLEDKLDLDHKSE-EDKRLKIRISHELDSASSEVN
                                                    :********:******:** **::***********.***
```

Table S1.3: Osteopontin in Artiodactyla, Cervidae and Bovidae. The N-terminus of the sequence for *Odocoileus virginianus texanus* is possibly incomplete. The available *Cervus elaphus hippelaphus* sequence may reflect transcription or translation from an alternative start site.

```
XM_006198167.2:Vicugna pacos           ------------------------------------------------------------
XM_010966141.1:Camelus bactrianus      ------------------------------------------------------------
XM_006175474.2:Camelus ferus           ------------------------------------------------------------
XM_010983105.1:Camelus dromedaries     ------------------------------------------------------------
NM_214023.1:Sus scrofa                 ------------------------------------------------------------
X16575.1:Sus                           ------------------------------------------------------------
XM_007102526.1:Physeter catodon        ------------------------------------------------------------
NW_006776832.1:Lipotes vexillifer      ------------------------------------------------------------
XM_022582289.1:Delphinapterus leucas   MTHLSTPCVGGERLEQPLNSETSWLSTAGRGRAQSAALSTASAPPGTGLFLAAADQGTLI
NW_004438431.1:Orcinus orca            ------------------------------------------------------------
XM_004315204.2:Tursiops truncatus      ------------------------------------------------------------

XM_006198167.2:Vicugna pacos           -MKLAVICFCLLGIASALPVGQTNSGSSEEKQLNNKYPDAVATWLKPDPSQKETFLAPQS
XM_010966141.1:Camelus bactrianus      -MKLAVICFCLLGIASALPVGQTNSGSSEEKQLNNKYPDAVATWLKPDPSQKQTFLAPQS
XM_006175474.2:Camelus ferus           -MKLAVICFCLLGIASALPVGQTNSGSSEEKQLNNKYPDAVATWLKPDPSQKQTFLAPQS
XM_010983105.1:Camelus dromedaries     -MKLAVICFCLLGIASALPVGQTNSGSSEEKQLNNKYPDAVATWLKPDPSQKQTFLAPQS
NM_214023.1:Sus scrofa                 -MRIAVIAFCLWGFASALPVKQTNSGSSEEKLLSNKYTDAVATLLKPDPSQKQTFLAPQN
X16575.1:Sus                           -MRIAVIAFCLWGFASALPVKQTNSGSSEEKLLSNKYTDAVATLLKPDPSQKQTFLAPQN
XM_007102526.1:Physeter catodon        -MRIAVICLCLLGIASALPVKQTDSGSSEEKQLYNKYPDAIATWLKPDPSQKQTFLAPQN
NW_006776832.1:Lipotes vexillifer      -MRTAVICFCLLGIVSALPVKQTDSGSSEEKQLYNKYPDAIATWLKPDPSQKQTFLEPQN
XM_022582289.1:Delphinapterus leucas   TMRIAVICFCLLGITSALPVKQTDSGSSEEKQLYNKYPDAVATWLKPDPSQKQTFLEPQN
NW_004438431.1:Orcinus orca            -MRIAVICFCLLGISSALPVKQTDSGSSEEKQLYNKYPDAVATWLKPDPSQKQTFLEPQN
XM_004315204.2:Tursiops truncatus      -MRIAVICFCLLGIASALPVKQTDSGSSEEKQLYNKYPDAVATWLKPDPSQKQTFLEPQN
                                        *: ***.:** *: ***** **:******* * *** **:** ********:*** **.

XM_006198167.2:Vicugna pacos           TASSEETDNFKQETLPSTSNESPDLTDDVDDDDDDTVDSQDVDSNDSDDHTDDTDRSDES
XM_010966141.1:Camelus bactrianus      TASSEETDNFKQETLPSTSNESPDHTDDVDDDDDDTVDSQDVDSNDSDDHTDDTDRSDES
XM_006175474.2:Camelus ferus           TASSEETDNFKQETLPSTSNESPDHTDDVDDDDDDTVDSQDVDSNDSDDHTDDTDRSDES
XM_010983105.1:Camelus dromedaries     TASSEETDNFKQETLPSTSNESPDHTDDVDDDDDDTVDSQDVDSNDSDDHTDDTDRSDES
NM_214023.1:Sus scrofa                 TISSEETDDFKQETLPSKSNESPEQTDDVDDDDDEDHVDSRDTDSEEADHADDADRSDES
X16575.1:Sus                           TISSEETDDFKQETLPSKSNESPEQTDDVDDDDDEDHVDSRDTDSEEADHADDADRSDES
XM_007102526.1:Physeter catodon        TVSSEETADNKQETLPRKSSESPEHTDTVDDEEDGDGQDADA------N---DDDHSDES
NW_006776832.1:Lipotes vexillifer      TVSSEET-DNKQETLPSKSNESPEHTDTVDDEEDGDSQDTDA------NDSKDADHSDES
XM_022582289.1:Delphinapterus leucas   TVSSEET-DNKQETLSSKSNESPEHTDTVDDEEDGDSQDTDA------NDSNDTDHSDES
NW_004438431.1:Orcinus orca            TVSSEET-VNKQETLPSKSNESPEHTDTVDDEEDGDSQDND---------SNDTDHSDES
XM_004315204.2:Tursiops truncatus      TVSSEET-DNKQETLPSKSNESPEHTDTVDDEEDGDSQDTDA------SDSSDTDHSDES
                                       * ****    *****  .*.***: ** ***::*    .             * *:****
```

```
XM_006198167.2:Vicugna pacos            HHSDESDEVVTDFPTDIPATAVFTPAVPTEDTQDGRGDNIGYALRTKSKKFRRSEVEHPE
XM_010966141.1:Camelus bactrianus       HHSDESDEVVTDFPTDIPATAVFTPAVPTEDTQDGRGDNIGYALRTKSKKFRRSEIEHPE
XM_006175474.2:Camelus ferus            HHSDESDEVVTDFPTDIPATAVFTPAVPTEDTQDGRGDNIGYALRTKSKKFRRSEIEHPE
XM_010983105.1:Camelus dromedaries      HHSDESDEVVTDFPTDIPATAVFTPAVPTEDTQDGRGDNIGYALRTKSKKFRRSEIEHPE
NM_214023.1:Sus scrofa                  HHSDESDELVTDFPTDTPATDV-TPAVPTGDPNDGRGDSVVYGLRSKSKKFRRSEAQQLD
X16575.1:Sus                            HHSDESDELVTDFPTDTPATDV-TPAVPTGDPNDGRGDSVVYGLRSKSKKFRRSEAQQLD
XM_007102526.1:Physeter catodon         HDSDESDEGVTDFPTDVPATSVFTPPVPTGDTYEGRGDSVAYGLRSKSKKFHRSEVQYPD
NW_006776832.1:Lipotes vexillifer       HHSDESDEVVTDFPTDVPATSVFTPPVPTGDTYEGRGDSVAYGLRSK-KMFHRSEVQYPD
XM_022582289.1:Delphinapterus leucas    HHSDESDEVVTDFPTDVPATSIFTPPVPTGDTYEGRGDSVAYGLRSK-KMFRRSEVQYPD
NW_004438431.1:Orcinus orca             HHSDESDEVVTDFPTDVPATSVFTPPVPTGDTYEGRGDSVAYGLRSK-KMFRRSEVQYPD
XM_004315204.2:Tursiops truncatus       HHSDESDEVVTDFPTDVPATYVFTPPVPTGDTYEGRGDSVAYGLRSK-KMFRRSEVQYPD
                                        *.****** ******* *** : ** *** *  :****.: *.**:* * *:*** :  :

XM_006198167.2:Vicugna pacos            A-----MTHVESEEADEAHKAIFDAQGLHTASDWNSQWKDSQETSQPGDHSVETHSQEHS
XM_010966141.1:Camelus bactrianus       A-----MTHVESEEADEAHKAIFDAQGLHMASDWNSQWKDSQETSQPSDHSVETHSQEHS
XM_006175474.2:Camelus ferus            A-----MTHVESEEADEAHKAIFDAQGLHMASDWNSQWKDSQETSQPSDHSVETHSQEHS
XM_010983105.1:Camelus dromedaries      A-----MTHVESEEADEAHKAIFDAQGLHTASDWNSQWKDSQETSQPSDHSVETHSQEHS
NM_214023.1:Sus scrofa                  ATEEDLTSHVESEETDGTPKAILVAQRLHVASDLDSQEKDSQETSQPDDRSVETRSQEQS
X16575.1:Sus                            ATEEDLTSHVESEETDGTPKAILVAQRLHVASDLDSQEKDSQETSQPDDRSVETRSQEQS
XM_007102526.1:Physeter catodon         AREEDISSHLESEEVGDAPKAILVAQRLHRASDWDSRGKDSQETSQPEERRMETHSREHS
NW_006776832.1:Lipotes vexillifer       ATEEDVTSHVESEEVGDAPKAILVAQRLHRASDWDSHGKDSQETSQPDDRSMETHSHEHS
XM_022582289.1:Delphinapterus leucas    ATEEDVTSHVESEEVGDAPKAILVAQRLHRASDWDSRGKDSQETSQPDDRSMETHSHEHS
NW_004438431.1:Orcinus orca             ATEEDVTSHVESEEVGDAPKAILVAQRLHRASDWDSRGKDSQETSQPDDRSMETHSREHS
XM_004315204.2:Tursiops truncatus       ATEEDVTSHVESEEVGDAPKAILVAQRLHRASDWDSRGKDSQETSQPDDRSMETHSREHS
                                        *      :*:****.. : ***: ** ** *** :*: ********* :: :**:*:*:*

XM_006198167.2:Vicugna pacos            EEYKLKADDEGDRHSDVVDSQENSQVSPEIHSQELPSHEDKLVLDPKSEEEDTHLKFRVS
XM_010966141.1:Camelus bactrianus       EEYKLKADDEGDRHSDVIDSQENSQVSPEVHSQELPSHEDKLVLNPKSEEEDTHLKFRVS
XM_006175474.2:Camelus ferus            EEYKLKADDEGDRHSDVIDSQENSQVSPEVHSQELPSHEDKLVLNPKSEEEDTHLKFRVS
XM_010983105.1:Camelus dromedaries      EEYKLKADDEGDRHSDVIDSQENSQVSPEVHSQELPSHEDKLVLNPKSEEEDTHLKFRVS
NM_214023.1:Sus scrofa                  KEYTIKTYDGSNEHSNVIESQENPKVSQE-----FHSHEDKLVPDSKSE-EDKHLKLRVS
X16575.1:Sus                            KEYTIKTYDGSNEHSNVIESQENPKVSQE-----FHSHEDKLVPDSKSE-EDKHLKLRVS
XM_007102526.1:Physeter catodon         KEFKLKAEVESNERSDVTDSQDNSKVSQE-----FHSHEDKVVPDLKSE-EDKHLKFRVS
NW_006776832.1:Lipotes vexillifer       KEFKLKAEDDSKEHSDVIESQDNSKVSQ-----EFHSREDKLVPDLKSE-EDKHLKFRVS
XM_022582289.1:Delphinapterus leucas    KEFKLKDEDDSNEHSDVIESQDNSKVSHEFHSQEFHSREDKLVPDLKSE-EDKHLKLRVS
NW_004438431.1:Orcinus orca             KEFKLKAEDDSYEHSDVIESQDNSKVSHEFHSQEFHSREDKLVPDLKSE-EDKHLKLRVS
XM_004315204.2:Tursiops truncatus       KEFKLKAEDDSYEHSDVIESQDNSKVSHEFHSQEFHSREDKLVPDLKSE-EDKHLKLRVS
                                        :*:.:*    . .:*:* :**:* :**       : *:***:* : *** **.***:***
```

XM_006198167.2:*Vicugna pacos*	HELDSASSEVN
XM_010966141.1:*Camelus bactrianus*	HELDSASSEVN
XM_006175474.2:*Camelus ferus*	HELDSASSEVN
XM_010983105.1:*Camelus dromedaries*	HELDSASSEVN
NM_214023.1:*Sus scrofa*	HELESASSEIN
X16575.1:*Sus*	HELESASSEIN
XM_007102526.1:*Physeter catodon*	HELDDASSEVN
NW_006776832.1:*Lipotes vexillifer*	HELDSASSEVN
XM_022582289.1:*Delphinapterus leucas*	HESDSASSEVN
NW_004438431.1:*Orcinus orca*	HELDSASSEVN
XM_004315204.2:*Tursiops truncatus*	HELDSASSEVN
	`** :.****:*`

Table S1.4: Osteopontin in Artiodactyla (Camelidae, Suidae and Celaceae).

```
XM_014787875.1:Ceratotherium simum simum      MRIAVICLCLLGIAYALPVQQANSGSSEEKLLYNKSPDAVGTWLKPDPSQKQTLLAPQTV
XM_001496152.4:Equus caballus                 MRIAVICLCLLGIAYALPVNQADSGSSEEKQLYNKHSDAVSIWLKPDPSQKQNLLAPQTV
XM_008523685.1:Equus przewalskii              MRIAVICLCLLGIAYALPVNQADSGSSEEKQLYNKHSDAVSIWLKPDPSQKQNLLAPQTV
XM_014842441.1:Equus asinus                   MRIAVICLCLLGIAYALPVNQADSGSSEEKQLYNKHSDAVSIWLKPDPSQKQNLLAPQTV
                                              *******************:**:******* ****  ***. **********.*******

XM_014787875.1:Ceratotherium simum simum      SSEETDDLKQETLPSKSNESPDHTDDVDDDDDGDHVDSQDSIDSDDSDETDPTDDPDQSE
XM_001496152.4:Equus caballus                 SSEETDNLKQETLPSQSNESHDHTDDVDDDDVGDHEDDQDSIDSDDSDETDPTDDPDNSD
XM_008523685.1:Equus przewalskii              SSEETDNLKQETLPSQSNESHDHTDDVDDDDVGDHEDDQDSIDSDDSDETDPTDDPDNSD
XM_014842441.1:Equus asinus                   SSEETDNLKQETLPSQSNESHDHTDDVDDNDDGDHEDDQDSIDSNDSDETDPTDDPDNSD
                                              ******:********:**** ********:* *** *.******:************:*:

XM_014787875.1:Ceratotherium simum simum      ESHHSDESDELVTDFPTDLPATPVFTPAVPTSDTYDGRGDSVGYGPRSKSKKVRRSEVQY
XM_001496152.4:Equus caballus                 ESHHSDESDELVTDFSTDVPATPVFTPAVPTRDTYDGRGDSLSYGLKSKSRKFRRSEDQY
XM_008523685.1:Equus przewalskii              ESHHSDESDELVTDFSTDVPATPVFTPAVPTRDTYDGRGDSLSYGLKSKSRKFRRSEDQY
XM_014842441.1:Equus asinus                   ESHHSDESDELVTDFSTDVPATPVFTPAVPTRDTYDGRGDSLSYGLKSKSRKFRRSEDQY
                                              *************** **:************ *********:.** :***:*.**** **

XM_014787875.1:Ceratotherium simum simum      PDATEEDLTSHVKSKEVGGVHKAILVAQGLHVASDWDSRGKDSLETSQLDDHSVETHSLE
XM_001496152.4:Equus caballus                 PDATEEDLTSPVESKDIDDVHKAVLVAQGLHVASDWDSRGKDSQETSQLDDHSVETHSRE
XM_008523685.1:Equus przewalskii              PDATEEDLTSPVESKDIDDVHKAVLVAQGLHVASDWDSRGKDSQETSQLDDHSVETHSRE
XM_014842441.1:Equus asinus                   PDATEEDLTSPVESKDIDDVHKAVLVAQGLHVASDWDSRGKDSQETSQLDDHSVETHSRE
                                              ********** *:**::..****:******************* ************** *

XM_014787875.1:Ceratotherium simum simum      HSKEYKLKANDETSEHSDVIDSHENSKFSHERHSQEFHSREDKLVLDRKSEEEDKYLKFR
XM_001496152.4:Equus caballus                 HSKEYQLKANDETSEHSDVIDSRENSKVSQEFPSQEFHSSEGKLVRDRKSEDEDKYLKFR
XM_008523685.1:Equus przewalskii              HSKEYQLKANDETSEHSDVIDSRENSKVSQEFPSQEFHSSEGKLVRDRKSEDEDKYLKFR
XM_014842441.1:Equus asinus                   HSKEYQLKANDETSEHSDVIDSLENSKVSQEFPSQEFHSSEGKLVRDRKSEDEDKYLKFR
                                              *****:**************** ****.*:*  ****** *.*** *****:********

XM_014787875.1:Ceratotherium simum simum      VSHELDSASSEVN
XM_001496152.4:Equus caballus                 TSHESESASSEVN
XM_008523685.1:Equus przewalskii              TSHESESASSEVN
XM_014842441.1:Equus asinus                   TFHESESASSEVN
                                              . ** :*******
```

Table S1.5: Osteopontin in Perissodactyla.

```
XM_013004835.1:Echinops telfairi              MRAAVLCCCLLGIAYALPVKHPADSGSSEEKQT---------------------------
NW_016536240.1:Manis javanica                 MRIAVICFSLLGIACTLPVK-QADSRSSEEKQLYQKYPDAVASWLKSDPSHKQTLLALQN
NW_006408627.1:Chrysochloris asiatica         MRIAVICFCLLGIAYALPAKYHTESGSSEEKQLHIKYPDAVATWLKPDPSQKQTLLAPQK
XM_003414088.3:Loxodonta africana             MRTAVICFCLLGIAYALPVKYQADSGSSEEKQLHSKHPDTVATWLKPDVSYKQILLAPQN
XM_012559624.2:Trichechus manatus latirostris MRIAVIYFCLLGIAYALPVKHQADSGSSEEKQLYSKYPDAVDTWLKPDPSYKQILLAPQN
                                              ** **:  .***** :**.*  ::* ******

XM_013004835.1:Echinops telfairi              --------------FPSKSYQSHDQTDDVDA-DDSDHSKSQDSVESDDSDDDDHADEPDH
NW_016536240.1:Manis javanica                 TVSSEETDNFKQETLPSRSNESHD---DVDDEEDGDNRDSQDSADASKSDDVDHTDDPDD
NW_006408627.1:Chrysochloris asiatica         AISSEEIDHLKQETLPSKSNESQDLTDDVDDEDDSDHVNNQDSVESDDSEDHDDTDDPDR
XM_003414088.3:Loxodonta africana             AVSSEENDKLKQETLPSKSNESHDQTDNVDDEDDGDHVDSQDSVDSDDLDDDDHTDDPYH
XM_012559624.2:Trichechus manatus latirostris AVSSEETDDLKQETLPSKSSESQDQTDSVDDEDDGEQVDSQDPIDSDDTHEDDHTDDSNH
                                                            :**:* :*:*   .**  :*.:: ..**  ::.. .: *.:*:

XM_013004835.1:Echinops telfairi              SDESHHSDESDEVTT----DVPATVLFTSALPTDFYSDDGRGDSLGYAVRSKLRRIHEAV
NW_016536240.1:Manis javanica                 S------DESDELVT----DVPATPVFTPADPTG-DTNDGRGDSVAYGLRSKSKKFYRSA
NW_006408627.1:Chrysochloris asiatica         SDESHHSDESDELVTDFPTDMSATPVFTPVVPTV-DIYDGRGDSVAYGQKSRSRKISTPV
XM_003414088.3:Loxodonta africana             SDESHHSDESDELVTGFPTDDPGTPVFTPAVPTV-DTYDGRGDNVVYRLSSKSKKSHKSV
XM_012559624.2:Trichechus manatus latirostris SNESNHSDESDELVTDFPTDDPATSVFTPAVPTV-DTYDGRGDSVVYRLRSKSKKIPRSD
                                              *      *****:.*    *  .* :** . **     *****.: *   *: ::

XM_013004835.1:Echinops telfairi              IQFPDDFASD-----------ENVKAIFPSQVLKKVSSSESQQRDSRETRQPDD--HSVE
NW_016536240.1:Manis javanica                 LQYPDATEEDLTSQMESKEIDKARKATLVARSLRVPSDWDSQET-----SQLDDQSQSAE
NW_006408627.1:Chrysochloris asiatica         ILYPDD----LTSLKKSEAFGDEHKAMSVMQAMKKVSNWDSQQQGSHETSQPDD--HSVE
XM_003414088.3:Loxodonta africana             IQFPDATEEDFTSRMESKELDDAHKAIRVVKSLKRASDWDSHQQDSHETSQLDD--HSVE
XM_012559624.2:Trichechus manatus latirostris IQHPHATEEDSISQMESKMLDDAHKAIPVISSLKKPFDWDSYQQDSRETSQQDD--HSVE
                                              :  .*.                .  **      ::    .  :* :        * **   :*.*

XM_013004835.1:Echinops telfairi              TQSHERSKESALETEVESSERSDRLNSQESSKVSQEAQSQEFHSHED------------
NW_016536240.1:Manis javanica                 TNSQGYSKEYTLKAKDESNEHADVIGNKEDSKVSQ-----EFS-HEDKLVFDPKSREED-
NW_006408627.1:Chrysochloris asiatica         THSLEQSKEYKLKANDESSEHSHMTDSLENSKVSQ-----EFHSHEDKHLPSPKSEDEDQ
XM_003414088.3:Loxodonta africana             THSQEQSKEYKRKANDVSSEHSDMIDSQENSKVSH-----EFHSHEDKHRPDPESEEQDK
XM_012559624.2:Trichechus manatus latirostris THSQEQYKVYKLKANDVSSEHSDTIHSQEDSRVSD-----ELHSREDKYYPDPKSEEEDK
                                              *:*     *     :::  *.*::.    . *.*:**.      *:  :**

XM_013004835.1:Echinops telfairi              ------------------
NW_016536240.1:Manis javanica                 KLKFHTSHELDSESSEAN
NW_006408627.1:Chrysochloris asiatica         QLKFRTSHELESTSYEIN
XM_003414088.3:Loxodonta africana             HLKFLISHELES------
XM_012559624.2:Trichechus manatus latirostris HLKFRISHELES------
```

Table S1.6: Osteopontin in Afrotheria and Xenarthra.

```
XM_001370458.3:Monodelphis domestica      -MRTAVICFCLLGIVSALPVKHQINSGSSEERRLYNKHPNLVATWLNADPSQKQTLLATQ
XM_003772957.3:Sarcophilus harrisii       MMRTAVICFCLLGIVSALPVKQQINSGSSEEKQLYSKHPNFVATWLNADPSQKQTLLATQ
XM_020988577.1:Phascolarctos cinereus     -MRTTVICFCLLGIISAWPVKQQINSGSSEEKPLYSKHPDFVATWLNDDPSQKQTLLATQ
                                           ***:*********:** ***:*********: **.***::****** ************

XM_001370458.3:Monodelphis domestica      NSVSSEESTEDLQQTLPSNSNESPDTTDDVDDDDDDDGHKSTDSDDSDESDEVVTDFPTD
XM_003772957.3:Sarcophilus harrisii       NSLSSEESTEDLQETLPRNSSESPDDIDDEDDDDGDH-NKSIDSDDSDESDEVVTDLPTD
XM_020988577.1:Phascolarctos cinereus     NSVSSEESTEDLQETLPTNSDEIPDDIDDDDNEDGDEGHKSTDSDDSDESDEVVTDFPTA
                                          **:**********:*** **.* **  ** *::*.*. :** **************:**

XM_001370458.3:Monodelphis domestica      IPTTSSFLPDGPTRGDNSGRGDSVAYGLRSKVGAPDISSAETHEVTEEDLTSQMESYESE
XM_003772957.3:Sarcophilus harrisii       TPATPSFLPDGPTRGDNGGRGDSVAYGLRSKLGAPYRSSEQVHDVTEEDLTSQIESYESE
XM_020988577.1:Phascolarctos cinereus     TPVTSSFLPDGPTRGDNGGRGDSVAYGLRSKVGVPYRSSEQVHDITEEDLTSQMESYESE
                                           *.* ************.*************:*.*  ** :.*::********:******

XM_001370458.3:Monodelphis domestica      KAHKAFPLSQNLPKVSAWGSNSKESNEASHPDEYSVETYSHEQFKSYQLEETNPDSQQQG
XM_003772957.3:Sarcophilus harrisii       KTHKAIPLSQTFPKVSSWESNGQESNEASQADEYSVETHSHEQLKSSQLERNIYDSQQQS
XM_020988577.1:Phascolarctos cinereus     KIHKVFPLSQNLAKVSAWNSNSKDSNEASHPDEHSVETQSHEQLKSYRLEQNSYDSQQQS
                                          * **.:****.: ***:* **.::*****: **:**** ****:** :**..  *****.

XM_001370458.3:Monodelphis domestica      DSHDSQENDKVSQEFR------------TQEVNKLSQEFQSQESHPVNDPESVENTKYLK
XM_003772957.3:Sarcophilus harrisii       DSHGSQENDKVSQEFHNREVDRDSQEFHKQQVGKLSQEYHSQEVHLVSDPESVENIKPLK
XM_020988577.1:Phascolarctos cinereus     DSHGSQENDKVSQEFHSPEVDKDSQEFRKLQVNKISQEFRSQEVHLANDPESVENAKHLK
                                          ***.***********:             . :*.*:***::*** * ..******* * **

XM_001370458.3:Monodelphis domestica      LHSFPEADSASYEAH
XM_003772957.3:Sarcophilus harrisii       LHS-HEVDSASFETH
XM_020988577.1:Phascolarctos cinereus     LHSSHEIDSASSEVH
                                          ***  * **** *.*
```

Table S1.7: Osteopontin in Marsupialia.

```
XM_008574253.1:Galeopterus variegatus   --------MRIAVICSCLLGIIYALPVKQPDSGSSEEKQLYNKYPDAMAAWLKPDPSQKQ
XM_016213045.1:Miniopterus natalensis   --------MRAAVICFCLLGIAYALPVKRADSGSSEERQLY-KHPDAVATWLKPDPAQKQ
XM_019668067.1:Hipposideros armiger     --------MRIAVVCFCLLGIAYTLPVKHIDSGSSEEKQLYQKYPDAVATWLKPDPSQKQ
XM_019719344.1:Rhinolophus sinicus      --------MRIAVICFCLLGIAYTLPVKHADSGSSEERQLYNKYPDAVATWLKPDPSQKQ
XM_016161109.1:Rousettus aegyptiacus    --------MRIAVICFCLVGIAYALPVKHADSGSSEERQLFNKYPDAVATWLKPDPSQKQ
XM_011356310.2:Pteropus vampyrus        --------MRIAVICFCLVGMAYALPVKHADSGSSEERQLFNKYPDAVATWLKPDPSQKQ
XM_006910714.2:Pteropus alecto          --------MRIAVICFCLVGMAYALPVKHADSGSSEERQLFNKYPDAVATWLKPDPSQKQ
XM_014452112.2:Myotis lucifugus         ------MTCTVDTSQPLSPPPPSSPKGLMNKVTMVAAMELYNKHPDAVATWLKPDPSQKQ
NW_007370672.1:Eptesicus fuscus         --------MRTAVIFFCFLAITYAFPVKHADSGSSEERQLYNKHPDAVATWLKPDPSQKQ
KE161812.1:Myotis brandtii              --------MRIAVICFCLLGIAYALPVKHADSGSSEERQLYNKHPDAVATWLKPDPSQKQ
KB104739.1:Myotis davidii               --------MRIAVICFCLLGITSALPVKHADSGSSEEKQLYNKHPDAVATWLKPDPSQKQ
                                                * :     :             .       :*: *:***:*:******:***

XM_008574253.1:Galeopterus variegatus   NLLAPQNAVSSEDIDNFKQETLPSKSNESHDHMDDENDEDDGDHVDSQDSIDSDDFDDT-
XM_016213045.1:Miniopterus natalensis   NLLAPQNTVSSEETDDFKQETLPSTSNESHDLTDDVDDDDDSNHVDSQDSTDSNDSDDA-
XM_019668067.1:Hipposideros armiger     NLLAPQNAVSSEETDDLKQETLPSASHESHDLTDDMDDDD--DHVDSQDSTDSTDSDDA-
XM_019719344.1:Rhinolophus sinicus      NLLAPQNAVSSEETADFKQETLPSTSNESHDLTDDVDDDDD-EHVDSQDSIDSNESDDAD
XM_016161109.1:Rousettus aegyptiacus    NLLAPQNTVSSEETDNFKQETLPSQSNESHDNTDDVDDEDDEDHVDSQDHIDSDDSDDA-
XM_011356310.2:Pteropus vampyrus        NLLAPQNTVSSEETDNFKQETLPSQSNESHDHTDDVDDEDDEDHVDSQDHIDSDDSDDA-
XM_006910714.2:Pteropus alecto          NLLAPQNTVSSEETDNFKQETLPSQSNESHDHTDDVDDEDDEDHVDSQDHIDSDDSDDA-
XM_014452112.2:Myotis lucifugus         SLLAPQNTVSSEETDDFKQETLPSKSNESQDHTDDVGDD-DEDRVDSQDSIDSNDSDDD-
NW_007370672.1:Eptesicus fuscus         NLLAPQNTVSSEETDDFKQETLPSKSNESQDHTDDVDDDDDEDHVDSQDSIDSNDSDDD-
KE161812.1:Myotis brandtii              SLLAPQNTVSSEETDDFKQETLPSKSNESQDHTDEVDD-DDEDHVDSQDSIDSNDSDDD-
KB104739.1:Myotis davidii               SLLAPQNTVSSEETDDFKQETLPSQSNESQDHTDDVDDDDDEDHVDSQDSIDSNDSDDD-
                                        .******:****:  ::******* *:**:*  *: .*    ::*****  ** : **

XM_008574253.1:Galeopterus variegatus   DHPDDSHHSDESHHSDESDELVTDF----PTRVPATPV-FTPAVPTIDTYDGRGDSVAYR
XM_016213045.1:Miniopterus natalensis   DQTDDSDNSDESHHSDESDELVTDF----PTDFPGTLI-VTPPVPTRDTYDGRGDSVAYG
XM_019668067.1:Hipposideros armiger     DHPDDPDNSDESHHSDESDELVTDF----PTDLPGTPI-VTPAPPTRDTYNGRGDSVAYG
XM_019719344.1:Rhinolophus sinicus      DRPDDSDTSDESNQSDESDEVVTDF----PTDLPGTPGVVTPPVPTRDTYNGRGDSVAYG
XM_016161109.1:Rousettus aegyptiacus    DNTDDSDNSDESHHSDESDELVTDF----PTDIPETPV-FTPAIPTRDTNDGRGDSVAYR
XM_011356310.2:Pteropus vampyrus        DHTDDSDNSDESHHSDESDELVTDF----PTDFPGTPV-VTPAVPTRDTNDGRGDSVAYR
XM_006910714.2:Pteropus alecto          DHTDDSDNSDESHHSDESDELVTDF----PTDFPGTPV-VTPAVPTGDTNDGRGDSVAYR
XM_014452112.2:Myotis lucifugus         DRTDDPDNSDESHHSDESDELVTDF----PTDFPGTPF-ATPAVPTIDTNDGRGDSVAYG
NW_007370672.1:Eptesicus fuscus         DHTDDPDTSDESHHSDESDELVTDF----PTDFPGTPF-ATPAVPTIDTNDGRGDSVAYG
KE161812.1:Myotis brandtii              GHTDDPDNSDESHHSDESDELVTDF----PTDFPGTPF-ATPAVPTIDTNDGRGDSVAYG
KB104739.1:Myotis davidii               DHTDDPDNSDESHHSDESDELVTDFPTDFPTDFPGTPF-ATPAVPTIDTNDGRGDSVAHG
                                        ..  ** . ****::******:****    ** .* *    **  ** ** :*******:
```

```
XM_008574253.1:Galeopterus variegatus     LRSKSKKFHRADVQYPDTTEEDLTSHVESEEFDDAH---KAIPVAQGLSVPSDWDSHGKD
XM_016213045.1:Miniopterus natalensis     LKSKSKKFHRPAAQYPDATEEDLTSHVESNEADEAH---KAVLVAQGLHVASDWDSHGRE
XM_019668067.1:Hipposideros armiger       LRSKSKKFHRSAIQYPDATEEDLTSHMESEEMDDAH---KAVLVAQGLQVASDWDSHGKD
XM_019719344.1:Rhinolophus sinicus        LRSKSKKFHRSAVQYPDATEEDLTSHVESEDVDDAH---KAILVAQGLQVASDWDSHGKD
XM_016161109.1:Rousettus aegyptiacus      LKSKSKKFHRSVVQYPDATEEDLTSHVESKEMDDVRKAVKAVFVAQGLHVASDWDRPGKD
XM_011356310.2:Pteropus vampyrus          LKSKSKKFHRSVVKYPDATEEDLTSHVESKEVDDARKAVKAVFVAQGLRVASDWDSPGKD
XM_006910714.2:Pteropus alecto            LKSKSKKFHRSVVKYPDATEEDLTSHVESKEVDDARKAVKAVFVAQGLHVASDWDSPRKD
XM_014452112.2:Myotis lucifugus           LRLKSKKLHRSVVQYPDATEEDLTSHMESDEMDDAH---RAILVAQGLPVASDWDSRGKD
NW_007370672.1:Eptesicus fuscus           LRLKSKKLHRSGVQYPDATEEDLTSHMESDEMGDAH---RAILVAQGLPVASDWDSRGKD
KE161812.1:Myotis brandtii                LRLKSKKLHRSVVQYPDATEEDLTSHMESDEMDDAH---RAILVAQGLPVASDWDSQGKD
KB104739.1:Myotis davidii                 LRLKSKKLHRSVVQYPDATEEDLTSHMESDEMDDAH---RAVLVAQGLPVASDWDSRGKD
                                          *: ****:**   :***:********:**.: .:.:   :*: ***** * ****   ::

XM_008574253.1:Galeopterus variegatus     SHETSKLDDQSVETYSYKQSKLHKRKASDESNEHSDGIDSREHSKDSPEFHSREFHSHED
XM_016213045.1:Miniopterus natalensis     SQEISLLDDHSVETHSLKLPKEYQLKAIGQSNEHSDGIDSPENVKISHEFHSQELGSYED
XM_019668067.1:Hipposideros armiger       SQETSQLDDHSVETLSRKLSKEYKLKANDESHEHPDVIDSQESSKVSHEFHSQEFHSHED
XM_019719344.1:Rhinolophus sinicus        SQETSQLDDHSVETYSHKHPKEYKLKAKDESHEHSDVIDSQENSKVSHEFHSQEFHSLED
XM_016161109.1:Rousettus aegyptiacus      SQETSQLNDHSTETRSLEHSEAYKLKANDESNGHADVIDSRENSKVSHEFHSREFHSRED
XM_011356310.2:Pteropus vampyrus          SQETSQLDDHSTETHSLEHSEEYKLKANDESNGHADVIDSQENSKVSHEFHSREFDSRED
XM_006910714.2:Pteropus alecto            SQETSQLDDHSTETHSLEHSEEYKLKANDESNGHADVIDSQENSKVSHEFHSQEFDSRED
XM_014452112.2:Myotis lucifugus           SQETSQLDDHSVETHSHEAAKEYKLKAMDASNEHSVGIDSQENSRVSHEFHSRELQSHED
NW_007370672.1:Eptesicus fuscus           SQETRHLDDHSVEIHSQELAKEYKLKAMDASNEHSDGIDSQENSRVSHEFHSRELHSHED
KE161812.1:Myotis brandtii                SQEASQLDDHSVETHSREAAKEYKLKAMDASNEHSVGIDSQENSRVSHEFHSRELQSHED
KB104739.1:Myotis davidii                 SQETSQLDDHSVETHSHELAKGYKLKAMDASNEHSVGIDSQENSRVSQEFHSRELQSHED
                                          *:*   *:*:*.*  * :  : :: ** . *: *   *** *  : * ****:*: * **

XM_008574253.1:Galeopterus variegatus     KLVLDPKSKEEDKQLKFRISHELESASSEVD
XM_016213045.1:Miniopterus natalensis     KPALHPKSEEQDKHLKFRVSHELESASTEVN
XM_019668067.1:Hipposideros armiger       KLVPDPKSQEQDKHLKFRISHELESASSEVN
XM_019719344.1:Rhinolophus sinicus        KLVLDPKSEEQDKHLKFRVSHELESASSEVN
XM_016161109.1:Rousettus aegyptiacus      KLALDPKSEEQDKHLKFRISHELESASSEVN
XM_011356310.2:Pteropus vampyrus          KLALDPKSEDQDKHLKFRISHELESASSEVN
XM_006910714.2:Pteropus alecto            KLALDPKSEDQDKHLKFRISHELESASSEVN
XM_014452112.2:Myotis lucifugus           KLASAPQSEEQDKHLKFRISHELESASSEVN
NW_007370672.1:Eptesicus fuscus           KLASDPQSEEQDKHLKFRISHELESASSEVN
KE161812.1:Myotis brandtii                KLASAPQSEEQDKHLKFRISHELESASSEVN
KB104739.1:Myotis davidii                 KLASDPQSEEQDKHLKFRISHELESASSEVN
                                          * .  *:*:::**:****:********:**:
```

Table S1.8: Osteopontin in Chiroptera. The *Myotis lucifugus* sequence possibly reflects transcription or translation from an alternative start site. The duplication of PTDF in only one representative of *Myotis* could be a mutation or an error in data entry.

```
XM_016191814.1:Erinaceus europaeus                        MKPLEQHLNSGQCCLPAASQQRRQTRTASSRTGFLSLCKKQGPTTTMRTVVICLCLLGIV
XM_012523397.2:Dasypus novemcinctus                       ------------------------------------------YARPCTRNTLTLSSFFIF
XM_007951712.1:Orycteropus afer afer                      ----------------------------------------------MRIAVICFCLLGVA
XM_007184118.1:Balaenoptera acutorostrata scammoni        ----------------------------------------------MRIAVICFCLLGIA
                                                                                                              .: :. : :

XM_016191814.1:Erinaceus europaeus                        SAFPVKQ-TDSGSSEETH--------------------------NAVS---TEDFSQET
XM_012523397.2:Dasypus novemcinctus                       SVSKVKHQADSGSSEEKQLYDKYPDAVAAWLKPDPSQKETLLAPQNAVSSEETDNFKQDT
XM_007951712.1:Orycteropus afer afer                      STFPVKRQADSGSSEEKQLYNKYPDAVATWLKPDPSHKQTLLASQNTLSSEETDDLKQET
XM_007184118.1:Balaenoptera acutorostrata scammoni        SALPVKH-TDSGSSEEKQLYNKYPDAIATWLKPDPSQKQTFLAPQNTVSSEETADNKQET
                                                          *.  **: :*******.:                              .: .* : .*:*

XM_016191814.1:Erinaceus europaeus                        APSSSNESPEHTDDADDRDGDSLQDALDSHNSDEDDDDDEAEDTDSSDESHHSDESDETT
XM_012523397.2:Dasypus novemcinctus                       LPSKSNESHDHTDNADDEDDGDHVD---SRDSDDSNESDHNDDPDHSDESHHSDESDEVV
XM_007951712.1:Orycteropus afer afer                      LPSKSNESLDQTDDVDDEDDGDHVD---SQESDDSEDHDHTDDPYHSDESHHSDESDELV
XM_007184118.1:Balaenoptera acutorostrata scammoni        LPSKSNESPEHTDTVDDEDDGDSQD---TDAN-------DSNDADHSDESHHSDESDEVV
                                                           **.**** ::** .**.*...  *   :  .       . :*   ************ .

XM_016191814.1:Erinaceus europaeus                        TEFPTDNPATIVFTPAFPTGD-YDGRGDSVAYGLRSKAKKLHRLDVQ---ETEEDLTSYM
XM_012523397.2:Dasypus novemcinctus                       TDFTTDAPATP----VVPTEDAYNGRGDSVAYGLESKS-KLYRSKIQFPDATEEDLTSHM
XM_007951712.1:Orycteropus afer afer                      TDFPTEVPATPVFTPAVPTVETYDGRGDSVASRLKSKSKKIYRSVIQYPDDTE-ELTSHM
XM_007184118.1:Balaenoptera acutorostrata scammoni        TDFPTDVPATSVFTPPVPTGDTYDGRGDSVAYGLRSKSKKFRRSEVQYPDATEEDVTSHV
                                                          *:* *: ***      .** : *:*******  *.**: *: *  :*    ** ::**::

XM_016191814.1:Erinaceus europaeus                        ESREGGDPHK---VAKHLQVSATWDSDPSPGKS---------------------------
XM_012523397.2:Dasypus novemcinctus                       EREGLDDAHRVIHVARRLNRASDWDSHGKDSQETRQLD-DHSVETHSHEQSKS--RKASH
XM_007951712.1:Orycteropus afer afer                      RSKELDDVPKAISVVHVLKRASDWDSHPQDSHEVSQLEISHSVETHSHEQSK--ELEAKD
XM_007184118.1:Balaenoptera acutorostrata scammoni        ESEEVGDAPKAILVAQRLHRSSDWDSRGKDSQETSQPD-DRSVETHSREHSKEFKLKAED
                                                          . .  .*  :.   .: *: :: ***  .  .:.

XM_016191814.1:Erinaceus europaeus                        ---------------GVSH-----ESHSLENSLPVEPQS-QEDRQLKFRVSHELDSASSE
XM_012523397.2:Dasypus novemcinctus                       ESNEHSDVIDSRENSKVSHEFNSQESHSHEDKLVLGPKSKEEDKHLKFHTSHELESASSE
XM_007951712.1:Orycteropus afer afer                      ESNERSDMIDSQEISKVSHEFHSQEFHSQEDKPHRDPKSVEEEQHLKFRVSHELDSASFE
XM_007184118.1:Balaenoptera acutorostrata scammoni        ESSERSDVIESQDNSKVSHELHSQEFHSREDKLVPDLKS-EEDKHLKFRVSHELDSASSE
                                                                          ***     * ** *:.     :* :*:::***:.****:*** *

XM_016191814.1:Erinaceus europaeus                        VN
XM_012523397.2:Dasypus novemcinctus                       IN
XM_007951712.1:Orycteropus afer afer                      TN
XM_007184118.1:Balaenoptera acutorostrata scammoni        VN
                                                           *
```

Table S1.9: Osteopontin in various Eutheria. The available *Dasypus novemcinctus* sequence may be reflective of transcription or translation from an alternative start site.

```
XM_012934005.1:Sorex araneus              ------------------------------------------------------------
XM_004681418.2:Condylura cristata         ------------------------------------------------------------
XM_006885312.1:Elephantulus edwardii      ------------------------------------------------------------
XM_012928520.1:Ochotona princeps          ------------------------------------------------------------
KB320503.1:Tupaia chinensis               MARLWVQVQRPLRGLRAHVYDEGQAMSGMERGIGPIESAVTFKAREDQAAGSRQQPEEAG
D11411.1:Cryctolagus cuniculus            ------------------------------------------------------------

XM_012934005.1:Sorex araneus              -------------------------------------MRTAVICLCLLGIASALPVKTT-
XM_004681418.2:Condylura cristata         -------------------------------------MRIAVICFCLLGIVSALPVKQG-
XM_006885312.1:Elephantulus edwardii      -------------------------------------MRIAVICFCLLGIAYALPVKHQA
XM_012928520.1:Ochotona princeps          -------------------------------------MRIAMISFCLLGMAYALPIKHS-
KB320503.1:Tupaia chinensis               ASHVGTAQLLVGTRPGEPAVRSAAVRAFQGFPWKNHNMRVAVICFCLIGIAYALPVKQP-
D11411.1:Cryctolagus cuniculus            -------------------------------------MRIAVICFCLLGMAYALPVKHA-
                                                                               ** *:*.:**:*:. ***:*

XM_012934005.1:Sorex araneus              DSGSSEEKQ-----------------------------------------TLPSKSTESE
XM_004681418.2:Condylura cristata         DSGSSEKEQ-----------------------------------------SLPSVSDESN
XM_006885312.1:Elephantulus edwardii      TSGSSEEKQLNLKYPDAVATWLKPDPSHKQALLPPQTAVSSEETDLLKQETLPSQSNESP
XM_012928520.1:Ochotona princeps          DSGSSEEKQLYQKSPDDLAAWLSPDPSQKQNLLSVKDDASSEEKSDFKQQTLPSKLQESH
KB320503.1:Tupaia chinensis               DSGSSEERQLYNKYPDAVATWLKPDPSLKQNLLAPQHAVSSEETDDLKQETLPSTSNESH
D11411.1:Cryctolagus cuniculus            DSGSSEEKQLYHKHPDALATWLNPDPSQKQNLLTPQNAMSSEEKDDLKQETLPSKSIESH
                                           *****:.*                                         :***   **

XM_012934005.1:Sorex araneus              DHTDDLDDDDQADSQ----DSVDSEDADDDDDDD-------NNSDHSDESEETTTDSSMD
XM_004681418.2:Condylura cristata         ----DVDDVDDGDNT----ESRDSTDSDDDDHSDD-----SDESHHSDESDEMATDSPTD
XM_006885312.1:Elephantulus edwardii      DHTDDVDDEDDIDHV----DSKDSTESDDSEDHTDDDPYHSDESHHSDESDELPTDSPTE
XM_012928520.1:Ochotona princeps          DHMDDIDEDEDHNDSHSFIDSNDADDDDDDD-NQADDSHHSDESHHSDESDEVVTDLLTE
KB320503.1:Tupaia chinensis               DHMDDVDDEDDGNHV----DSQDS-DDSDDT-DHPDDSHHSDESHHSDESDELVTDFPTD
D11411.1:Cryctolagus cuniculus            DHMDDIDEDEDDDHV----DNRDS-NESDDA-DHPDDSHHSDESHQSDESDEV-TVYPTE
                                              *:*: :: :      :. *: : .*:     *.  ..::*.:****:*  *    :

XM_012934005.1:Sorex araneus              VPATLVFTPAVPTRDTNDGRGDSVDYGLR-LKSKKLLSSETQYSE----DLTSHMESDED
XM_004681418.2:Condylura cristata         LPATVDYTPVVFTRDTYDGRGDSVAYGLK-SKSIKFYSAEDQYPDATKEDLASQMESREM
XM_006885312.1:Elephantulus edwardii      DPSTPVFSPAMPTVDPYDGRGDSLGYGLK-SRSRP----QIQYSD-ETEDFFSHVKSKEL
XM_012928520.1:Ochotona princeps          SPATTAFTEVVPTIETYDGRGDSVAYRLK-SKSKVLRISTAQFLGAAEDDLSSHVDSQDM
KB320503.1:Tupaia chinensis               GPVTPVFTPAAPTMDAYDGRGDSVVYRVR-SKPKTFRMSDVQYPDATEEDLASHLESKEL
D11411.1:Cryctolagus cuniculus            DAATTVFTEVVPTVETYDGRGDSVAYRLKRSKSKMFHVSNAQYPGASEEDLSSHVDSEDL
                                             *  :: .  * :  ******: * ::  .         *:      *: *::.* :
```

```
XM_012934005.1:Sorex araneus          TQE-----SAPSLQMTSDGDSHQEPAKSP-------------------------------
XM_004681418.2:Condylura cristata     RNGPKATLVALNLQSTSDVDSQGEDSFEKR---DDSL-ENNGFEHSQEYKLKANDESSQ-
XM_006885312.1:Elephantulus edwardii  KDAHKIIPVVQALKKSSVWDSYQQDSHEPSQLSDLHSMETHSQEQSKEYTLKTDDVSSE-
XM_012928520.1:Ochotona princeps      DDARKAVLVAQHLNAPSDWDNHD------DSKMDDHGMESQSRERVKQYKRDVKDDSMER
KB320503.1:Tupaia chinensis           DDEHKTIPIARALHVPSDWDSHGKGSQE-TSQLDDHSVETHSGEQAKRYKQKASVESNE-
D11411.1:Cryctolagus cuniculus        DDTPRAIPVAQHLNVPSDWDSQEKDSHD-VSQVDDHSVETQSHEQARQYKREANDNSVE-
                                       :       .  *:  *  *.

XM_012934005.1:Sorex araneus          ------------ASLESHSRESQSLEGDA---QSPRVEEAHLKFRVSHELESASSEAK
XM_004681418.2:Condylura cristata     ---------KTPESSHLSHSQEFHNQEEKLDLDPKSVEEDTHLKFRVSHELDSTSSEVN
XM_006885312.1:Elephantulus edwardii  HSDMLNSQEISKSNLEFHSQEFHSHEDKLHPDPKSTEE-EHLKFRNSHELESASSEAN
XM_012928520.1:Ochotona princeps      HSYSVDSRESSKVSQESH-----SHEDMLAIEPKSTEEDKPGKSSVSQELDSASSEIN
KB320503.1:Tupaia chinensis           HSDVIESQESAKVSHEFHSREFLSHEDKLVLNSKSQEEDKHLKFRVSHELDSASSEVN
D11411.1:Cryctolagus cuniculus        HSHSIDSQESSKVSQESQSREFRSHEDKLAIEPKSEEDEEHRQLRVSHELDSTSSEIN
                                                   .   :     . *       .   :    :   *:**:*:*** :
```

Table S1.10: Osteopontin in Lagomorpha and similar orders. As alternative splicing of exons 4 or 5 has only been shown to occur in humans, there is no experimental evidence that osteopontin actually misses an internal sequence in *Sorex araneus* or *Condylura cristata*.

```
XM_013029816.1:Dipodomys ordii                        -----MRIEVIFFCLIGITHSLPI---ESGSSEENQLFNKKYPDSEASWLKLDLSEKNNL
XM_023564932.1:Cavia porcellus                        -----MRVTVICFCLLGIASSFPVKQADSGSSEEKLHD-NKLPEAASSWLKSDPSQKQNL
XM_004629257.2:Octodon degus                          -----MRIAGICFCLLGIAYSLPVKEADSFSSEDHLRD-NKFPEAMALWLKTDPSLKENI
NW_004955474.1:Chinchilla lanigera                    -MMLMLYFSLSGATATGQRWQKRTHNITSAAEELDFLY-NKLPEAMSSWLKSDPSQKENL
KN124024.1:Fukomys damarensis                         -----MRIALICFCLIGIAYSLPVKQADSGSSEEKLLY-NKLPEAVSSWLKPDPSQKQNL
JH171653.1:Heterocephalus glaber                      -----MRIAVICFCLIGITYSLPVNQADSGSSEEKPLY-NKLPEAVSSWLKPDPSQKQNL
XM_005341895.3:Ictidomys tridecemlineatus             -----MRFAVIFFCLVGIAYSFPVKQADSGSSEERQLY-NKHPDAVATWLKPDPSQKQNL
XM_015499141.1:Marmota marmota marmota                -----MRFAVIFFCLLGIAYSFPVKQADSGSSEERQLY-NKHPDAVATWLKPDPSQKQNL
AB001382.1:Rattus norwegicus                          -----MRLAVVCFCLFGLASCLPVKVAEFGSSEEKAHY-SKHSDAVATWLKPDPSQKQNL
XM_021211046.1:Mus pahari                             -----MRLAVICFCLFGIASSLPVKVTDSGSSEEKM-----------------------
J04806.1:Mus musculus                                 -----MRLAVICFCLFGIASSLPVKVTDSGSSEEK-LY-SLHPDPIATWLVPDPSQKQNL
XM_021162825.1:Mus caroli                             -----MRLAVICFCLFGIASSLPVKVTDSGSSEEKMLY-SLHPDPIATWLVPDPSQKQNL
HM626723.1:Apodemus agrarius(partial)                 -----------------------------------------------------PDPSQKQTL
XM_021659705.1:Meriones unguiculatus                  -----MRVAVICFCLFGIAAALPVKVSVSGSSEERLLY-NKHEDAVATWLRPDPSEKQNL
XM_013350872.1:Microtus ochrogaster                   -----MRFAVICFCLIGIASSFPVKVTDSGSSEENLLS-SKHTDAVATWLEPDPSQKQNL
XM_006975432.2:Peromyscus maniculatus bairdii         -----MRFAVVCFCLFGIASSLPVKVADSGSSEKKLLY-SKHSDAVATWLEPDPSQKQNL
AJ890352.1:Mesocricetus auratus                       -----MRFAVICFCLFGIASSLPVKVADSGSSEEKLHY-SKHTDAVATWLEPDPSQKQNL
HQ647182.1:Cricetulus barabensis(partial)             -----MRLAVVCFCLFGIASSLPVKVADSGSSEQKLHY-SKHTDAVATWLETDPSQKQNL
XM_007638075.2:Cricetulus griseus                     -----MRFAVICFCLFGIASSLPVKVADSGSSEQKLHY-SKHTDAVATWLETDPSQKQNL
NW_004504357.1:Jaculus jaculus                        -----MRAAMLCVCLFGLASSLPVKQADSGSSEETLIY-NKHLDAVATWLKPDPSQKQNL
XM_020154612.1:Castor canadensis                      -----MRIAVIFFCLLGIAYSLPVKQPDSGSSEEKQLFNNKHADAVAIWLKPDPSQKQNL
XM_008830194.2:Nannospalax galili                     -----MKIAVICFCLFGIASSLPVKLIDSGSSEEKQLH-SKHPDAVATWLKPDPSQKQNL


XM_013029816.1:Dipodomys ordii                        LAPQNVLSSEETDVLQPETFSSNSNESH--MDELNE--DDIGNHMDSQDFTESDESDHDS
XM_023564932.1:Cavia porcellus                        LAPQNAMSSEETNDLKQETLPSTSNESHDHMDDLDDEDD--GDL-------DTD---DPN
XM_004629257.2:Octodon degus                          IVPQNSVSSEEDNGLQQETLPSNSNESHDHMDDEDDDDRDE----------DSVDT----
NW_004955474.1:Chinchilla lanigera                    LAPQDAVSSEETDDLKQETLPSNSNESHDHMDDVDDDDD--GDQ-------DTADSADTD
KN124024.1:Fukomys damarensis                         LESQNAVSSEETDDLKQETLPSNSNERHDHMDDVDDDDD--GDQ-------DSVDTDDDD
JH171653.1:Heterocephalus glaber                      LAPQNSVSSEETDDLKQETLPSNSNESHDHMDDVDDEDD--GDQ-------DSVDVDDDD
XM_005341895.3:Ictidomys tridecemlineatus             LAPQNAVSSEERDDFKQETLPSKSNESHDHMDDVDDDDD--GDHVNNQDSTDSDDSDED-
XM_015499141.1:Marmota marmota marmota                LAPQNAVSSEERDDFKQETLPSKSNESHDHMDDVDDDDD--GDHVNNQDSTDSDDSDDD-
AB001382.1:Rattus norwegicus                          LAPQNSVSSEETDDFKQETLPSNSNESHDHMDDDDD-DDDDGDHAESEDSVNS-------
XM_021211046.1:Mus pahari                             ----NAVSSEEKDDFKQETLPSNSNESHDHMDDDDDDDDDDGDHAESEDSVDS-------
J04806.1:Mus musculus                                 LAPQNAVSSEEKDDFKQETLPSNSNESHDHMDDDDDDDDDDGDHAGSEDSVDS-------
XM_021162825.1:Mus caroli                             LAPQNAVSSEEKDDFKQETLPSNSNESHDHMDDDDDDDDDDEDHAESEDSVDS-------
HM626723.1:Apodemus agrarius(partial)                 LALQNAVSSEEKDDFKQETLPSTSNESHDHMDDDDDDDDDDGDHAESEDSVDS-------
XM_021659705.1:Meriones unguiculatus                  LAPQNVVSSEETDDLTQETLPSNFEKRHDHEDNDDDDDDDDGDHTESKDSLDSDESDS--
XM_013350872.1:Microtus ochrogaster                   LAPQNTVSSEETDDLKQETLPSNSNESQDHTDDDD--DDDDGDHADSQDSVDSDESDDD-
XM_006975432.2:Peromyscus maniculatus bairdii         LAPQNVVSSEETDALKQDTLPSNSNESHDHMDDDD-DD-DDRDHADSQDSVDSDESDED-
AJ890352.1:Mesocricetus auratus                       LAPQNVVSSEETDDLKQETLPSNSNESHDHMDDDN-DDDDDGDHANSQDSVDSNESDDDD
HQ647182.1:Cricetulus barabensis(partial)             LAPQNVVSSEETDDLKQETLPSNSNESHDHMDDDD-DDNDDGDHANSQDSVDSDESDEDD
XM_007638075.2:Cricetulus griseus                     LAPQNVVSSEETDDLKQETLPSNSNESHDHMDDDD-DDNDDGDHANSQDSVDSDESDEDD
NW_004504357.1:Jaculus jaculus                        LAPQNTVSSEETNDFRQETLPSNSNESHDHMDDDD-DDDDDGDHVDSHDSVDSDDSEDTD
XM_020154612.1:Castor canadensis                      LAPQNVVSSEETDDIKQQTLPSNSNESHDHMDDMD--DEDDGDHVDSQDSVDSDDSDDTD
XM_008830194.2:Nannospalax galili                     LAPQNAVSSEETDDFKQETLPSNSNESHDHMDDDD---DDDRDHVDSQDSVDSDDTDDAD
                                                          : :**** : :  :*: *. :: :   *: :                ::
```

```
XM_013029816.1:Dipodomys ordii                         DHEHHSHHHDHHEHHKNSHHSHESEEVISL-PPPLTLETPVFTPVFPAMSTLE-SHGDSN
XM_023564932.1:Cavia porcellus                         D-------EDDAHHSNESDHSDESDEVVTGGPPRIGSETPTSQPIVLTIETFD-GRGDSL
XM_004629257.2:Octodon degus                           --------DDPDHHSDESDHSDESDEVVTEAP-TEGPETPAFSPIVPTVEIYD-GRGDSL
NW_004955474.1:Chinchilla lanigera                     D-------ADDSHHSDESDHSDESDEVVTEAP-TNGPETPVFTPIVPTVETYD-GRGDSL
KN124024.1:Fukomys damarensis                          H-------PDDSHHSDESDHSEESDEEVTDAP-TNSPETPVFTPIVPTIETYDDGRGDSP
JH171653.1:Heterocephalus glaber                       H-------PDDSHHSDESDHSDESDEVVTDAP-TNGPETPVFTPIVPTVETYD-GRGDSL
XM_005341895.3:Ictidomys tridecemlineatus              ----------DSHHSDESHHSDESDEVVTESP-TDGPDTPVFTQIVPTVETYD-GRGDSV
XM_015499141.1:Marmota marmota marmota                 ----------DSHHSDESHHSDESDEVVTESP-TDGPDTPVFTQIVPTVETYD-GRGDSV
AB001382.1:Rattus norvegicus                           ------------DESDESHHSDESDESFTAS-----TQADVLTPIAPTVDVPD-GRGDSL
XM_021211046.1:Mus pahari                              ------------EESDESHHSDESDETFTAS-----TQADTFTPIAPTVDVPD-GRGDSL
J04806.1:Mus musculus                                  ------------DESDESHHSDESDETVTAS-----TQADTFTPIVPTVDVPN-GRGDSL
XM_021162825.1:Mus caroli                              ------------DESDESHHSDESDETFTAS-----TQADTFTPIAPTVDVPD-GRGDSL
HM626723.1:Apodemus agrarius(partial)                  ------------NESDESH-----------------------------------------
XM_021659705.1:Meriones unguiculatus                   ----------------DSHHSDESEESVTA-----TLQTDVFTPLVPTVDTPA-ARGDSL
XM_013350872.1:Microtus ochrogaster                    -----------D-QPDDSHHSDESDESVTA-----TTQTEIFTPVVPTVEIPD-GRGDSL
XM_006975432.2:Peromyscus maniculatus bairdii          -----------D-HPDDSHHSDESDESFDT-----TTQTEVFTPVVPTVDIPD-GRGDSL
AJ890352.1:Mesocricetus auratus                        H-------PDDD-HPDDSHHSDESDESVTA-----TTQTEVFTPAVPTVEIPD-GRGDSL
HQ647182.1:Cricetulus barabensis(partial)              -------------HPGDSHHSDESDESITV-----TTQTEVFTPAVPTVEIPN-GRGDSL
XM_007638075.2:Cricetulus griseus                      -------------HPDDSHHSDESDESITV-----TTQTEVFTPAVPTVEIPN-GRGDSL
NW_004504357.1:Jaculus jaculus                         H-------HDDSHHSDESHHSDESDELVTDVP-TNGPQTQDFTPIVPTVETFD-GRGDSV
XM_020154612.1:Castor canadensis                       H-------PDDSHHSDESHHSDESDELFTSSP-TDNPGTQVFTPVVPTVEVYD-GRGDNV
XM_008830194.2:Nannospalax galili                      D-------PDDSHHSDESHHSDESDELFTDTP-TDIPQTEVFTPVVPTVYTVD-ARGDSL
                                                                       :*.

XM_013029816.1:Dipodomys ordii                         APEGKKSKSSDITNVQVSPFTDMFNGSKQSSLLGMQAVHIQLSFSKHKNIFSKEGKEGKE
XM_023564932.1:Cavia porcellus                         A-YGLESRS-KKLPV---------------------------------------------
XM_004629257.2:Octodon degus                           A-YSLRSRS-KKLHI---------------------------------------------
NW_004955474.1:Chinchilla lanigera                     A-YRLRSRS-TKLHI---------------------------------------------
KN124024.1:Fukomys damarensis                          A-YGLRSRS-KKFSI---------------------------------------------
JH171653.1:Heterocephalus glaber                       A-YGLRSRS-KKFHI---------------------------------------------
XM_005341895.3:Ictidomys tridecemlineatus              D-YGVRSKS-KKFYI---------------------------------------------
XM_015499141.1:Marmota marmota marmota                 D-YGVRSKS-KKFYI---------------------------------------------
AB001382.1:Rattus norvegicus                           A-YGLRSKS-RSFPV---------------------------------------------
XM_021211046.1:Mus pahari                              A-YGLRSKS-RSFQV---------------------------------------------
J04806.1:Mus musculus                                  A-YGLRSKS-RSFQV---------------------------------------------
XM_021162825.1:Mus caroli                              A-YGLRSKS-RSFQV---------------------------------------------
HM626723.1:Apodemus agrarius(partial)                  ------------------------------------------------------------
XM_021659705.1:Meriones unguiculatus                   A-YGLRA---RKLYL---------------------------------------------
XM_013350872.1:Microtus ochrogaster                    A-YGLQSKS-RKIHI---------------------------------------------
XM_006975432.2:Peromyscus maniculatus bairdii          A-YGLRSKS-RKFHI---------------------------------------------
AJ890352.1:Mesocricetus auratus                        A-YGLRAKS-RKFHI---------------------------------------------
HQ647182.1:Cricetulus barabensis(partial)              A-----------------------------------------------------------
XM_007638075.2:Cricetulus griseus                      A-YGLRSKS-RKFHI---------------------------------------------
NW_004504357.1:Jaculus jaculus                         V-YRLKSKS-RTFHI---------------------------------------------
XM_020154612.1:Castor canadensis                       A-YGLRSRYLKKFDI---------------------------------------------
XM_008830194.2:Nannospalax galili                      A-YGLRSKS-RKIHI---------------------------------------------
```

```
XM_013029816.1:dipodomys_ordii                  GKEGKEGKEGKEGKEGKEGKEGKESHDEHLEHKELHDTLRVIPIPHITHEDFSPHWES-K
XM_023564932.1:Cavia porcellus                  ---------------------------------------SDSQFSDVTDEDLTSHVKSQE
XM_004629257.2:Octodon degus                    ---------------------------------------SDAQDPDATDEDLTSHVESQE
NW_004955474.1:Chinchilla lanigera              ---------------------------------------SDAQDPDATDEDLTSHVESQE
KN124024.1:Fukomys damarensis                   ---------------------------------------SDAQYRDATDEDLTSHAESQE
JH171653.1:Heterocephalus glaber                ---------------------------------------SDPQYPAATDKDLTSNVESQE
XM_005341895.3:Ictidomys tridecemlineatus       ---------------------------------------SEVQYPDATDEDLTSHMESSE
XM_015499141.1:Marmota marmota marmota          ---------------------------------------SEVQYPDATDEDLTSHMESAE
AB001382.1:Rattus norwegicus                    ---------------------------------------SDEQYPDATDEDLTSRMKSQE
XM_021211046.1:Mus pahari                       ---------------------------------------SDEQYPDATDEDLTSHVNSGE
J04806.1:Mus musculus                           ---------------------------------------SDEQYPDATDEDLTSHMKSGE
XM_021162825.1:Mus caroli                       ---------------------------------------SDEQYPDATDEDLASHVNSGE
HM626723.1:Apodemus agrarius(partial)           ------------------------------------------------------------
XM_021659705.1:Meriones unguiculatus            ---------------------------------------SDEQYSDATDEDLPSQV----
XM_013350872.1:Microtus ochrogaster             ---------------------------------------PSDQFSDEIGEDLTSHLKSKE
XM_006975432.2:Peromyscus maniculatus bairdii   ---------------------------------------SDDQYPDATDEDFTSHMKSKE
AJ890352.1:Mesocricetus auratus                 ---------------------------------------SDDQYPDTTDEDLSSHMKSKE
HQ647182.1:Cricetulus barabensis(partial)       ------------------------------------------------------------
XM_007638075.2:Cricetulus griseus               ---------------------------------------SDDQDPDATDEDLSSHMKSKE
NW_004504357.1:Jaculus jaculus                  ---------------------------------------SDAKYPDATEEDLTSHMESEE
XM_020154612.1:Castor canadensis                ---------------------------------------SDAQFSDATEEDLTSHIESEE
XM_008830194.2:Nannospalax galili               ---------------------------------------SDIQYPDATHEDLTSHMESKD

XM_013029816.1:Dipodomys ordii                  PHDTLKVIPIPHHIRFPTEWDTHELEQHGLELRRSESKGSESH-------NNNNHNNGDQ
XM_023564932.1:Cavia porcellus                  SHEAHQAVLLAQHLNVPSDWDSHGKDSHESSQLDDLSVETHSQEHPREHTRKARDHSS--
XM_004629257.2:Octodon degus                    SSAAHKAVLVAQRLNVPSDWDSHGKDSHESSQLDVPSVESHSQEQSRERMRKARDNSG--
NW_004955474.1:Chinchilla lanigera              SSDAHKAVLVAQRLNVPSDWDSHGKDSHESSQLDDPSVETHSQEQSKEHKRKARDNSS--
KN124024.1:Fukomys damarensis                   SSDAHKAVLVAQHLNVPSDWDSHGKDSHESSQLDEQNVESHSQEQSREHKRKARDDSN--
JH171653.1:Heterocephalus glaber                SSDAQKAVLVAHRLNMPSDWDSRGKDSHESSQLDDPSVETHSQEQSREHKRKARNDSS--
XM_005341895.3:Ictidomys tridecemlineatus       LNDAHKAIPVAHRLKVPSDWDSHGKDSHETSQLDDHSVETHSQEQTREYKRNARDN----
XM_015499141.1:Marmota marmota marmota          LNDAHKVIPVAHRLKVPSDWDSHGKDSHETSQPDDHSVETHSQEQTREYKRNARDN----
AB001382.1:Rattus norwegicus                    SDEALKVIPVAQRLSVPSDQDSNGKTSHESSQLDEPSVETHSLEQSKEYKQRASHESTEQ
XM_021211046.1:Mus pahari                       SKEALKVIPVAQLLSVPSDQDSNGKASHESRQLDEPSLETHSLEQSKENK--ASHESADQ
J04806.1:Mus musculus                           SKESLDVIPVAQLLSMPSDQDNNGKGSHESSQLDEPSLETHRLEHSKESQ----------
XM_021162825.1:Mus caroli                       SKESLNVIPVAQLLSMPSDQDNNGKGSHESSQLDEPSLETHSLEHSKESH----------
HM626723.1:Apodemus agrarius(partial)           ------------------------------------------------------------
XM_021659705.1:Meriones unguiculatus            -------------YSVPSDQDSNGKTSHESSQLDELSAETYRDGQAEEHKQD-SHDS---
XM_013350872.1:Microtus ochrogaster             FDDVLKVIPVVHRLSVPSDQDSTGKTSHESSQLDEPSVETQSHEQSQEDKLKASHES---
XM_006975432.2:Peromyscus maniculatus bairdii   LDDALKVIPVAHRLNVPS-----GKTSQESSQMDEPSVETHSHEQSQEDKQKASHES---
AJ890352.1:Mesocricetus auratus                 LVDTLKVIPVAYRLNEPSDQDSTGKTSHESSQLDEPSVETHSHEQSQEHKQKASHES---
HQ647182.1:Cricetulus barabensis(partial)       ------------------------------------------------------------
XM_007638075.2:Cricetulus griseus               LVDTLKVIPVVHRLNMPSDQDSTGKTSHESSQLDEPSVETHSHEQSQEHKQKASHES---
NW_004504357.1:Jaculus jaculus                  SDDALKVVPVARHLHVPSDWDSHAKDSHESSQLGDQSVETQSHEQPREYKQKTSHES---
XM_020154612.1:Castor canadensis                SDDALKAIPVAQHLKVPSDWDS-----HESSQLDDHSVETHSREQSREHKQKASHES---
XM_008830194.2:Nannospalax galili               LDDLLKAVPVAQSLNVPSDWDS-----HESSQLDEPSVESHSHEQPREGKQKASHES---
```

XM_013029816.1:*Dipodomys ordii*	FSVSKKSKYKP---------DGPDS-L---------------------------------
XM_023564932.1:*Cavia porcellus*	-----------------EHSDAVDS-REGARASPALRSQEHHSQELRSQEHHSQELRSQE
XM_004629257.2:*Octodon degus*	-----------------EHSDVVDS-QEGAKASQEL------------------------
NW_004955474.1:*Chinchilla lanigera*	-----------------EHSDQIDS-QESARASQEL------------------------
KN124024.1:*Fukomys damarensis*	-----------------EHSDVIES-QEGARASQEL------------------------
JH171653.1:*Heterocephalus glaber*	-----------------EHSDVIDS-QEGARASQEL------------------------
XM_005341895.3:*Ictidomys tridecemlineatus*	---------------SSEHSDVIDS-QESSRVSQEFQ-----------------------
XM_015499141.1:*Marmota marmota marmota*	---------------SSEHSDVIDS-QESSRVSQEFQ-----------------------
AB001382.1:*Rattus norvegicus*	S-DAIDSAEKPDAIDSAERSDAIDS-QASSKASLEHQ-----------------------
XM_021211046.1:*Mus pahari*	----------SDVIESADQSDVIDS-QASFKASLEHQ-----------------------
J04806.1:*Mus musculus*	--------------ESADQSDVIDS-QASSKASLEHQ-----------------------
XM_021162825.1:*Mus caroli*	--------------ESADQSDVIDG-QASSKASLEHQ-----------------------
HM626723.1:*Apodemus agrarius*(partial)	--
XM_021659705.1:*Meriones unguiculatus*	----------------TEQSDEMYSKEKLSKASWEAQ-----------------------
XM_013350872.1:*Microtus ochrogaster*	----------------TELSDGIDS-KESSRASQAHA-----------------------
XM_006975432.2:*Peromyscus maniculatus bairdii*	----------------TELSDVIDS-KESSKASQEHQ-----------------------
AJ890352.1:*Mesocricetus auratus*	----------------TELSDVIDS-QESSKASQEHQ-----------------------
HQ647182.1:*Cricetulus barabensis*(partial)	--
XM_007638075.2:*Cricetulus griseus*	----------------TELSDVIDS-KESSKASQEHQ-----------------------
NW_004504357.1:*Jaculus jaculus*	----------------IEHSDGIDS-SESSKVSQEFH-----------------------
XM_020154612.1:*Castor canadensis*	----------------REHSDVIDS-QESSKVSQEVH-----------------------
XM_008830194.2:*Nannospalax galili*	----------------SEHADVIDN-RENSKASQESH-----------------------

XM_013029816.1:*Dipodomys ordii*	--GSSKVSHKLHSNEFLSQEDMLVLDPRSIE-DKHPNFRISHELDSTSSEAN
XM_023564932.1:*Cavia porcellus*	LRSQEHHSQELHSPELHSPELHSVEDPKSKEEDEHLKIRISHELDSASSEAN
XM_004629257.2:*Octodon degus*	----------------PSQEFPSHEDPQSKEEEGRLEVRVSQELDSASSEAK
NW_004955474.1:*Chinchilla lanigera*	----------------HSQEFHSHEDPQSREEGEHLKMRVSHELDSASSEAN
KN124024.1:*Fukomys damarensis*	----------------H-----SHEDPKSKQEDEHLKMSASRELDSASSEAN
JH171653.1:*Heterocephalus glaber*	----------------HSQEFPSHEDPKSKEEDEHLKIRVSHELDSVSSEAN
XM_005341895.3:*Ictidomys tridecemlineatus*	--SHES----------HSKEDKLVLDPKSKE-DKHLIFHVSHEIESASSEVN
XM_015499141.1:*Marmota marmota marmota*	--SHES----------HSNEDKLVLDPKSKEEDKHMIFRVSHEIESASSEVN
AB001382.1:*Rattus norvegicus*	--SHEF----------HSHEDKLVLDPKSKEDDRYLKFRISHELESSSSEVN
XM_021211046.1:*Mus pahari*	--SHKF----------HSHEDKLVLDPKSKEDDRYLKFRFSHELDSSSSEIN
J04806.1:*Mus musculus*	--SHKF----------HSHKDKLVLDPKSKEDDRYLKFRISHELESSSSEVN
XM_021162825.1:*Mus caroli*	--SHKF----------HSHKDKLVLDPKSKEDDRYLKFRISHELESSSSEVN
HM626723.1:*Apodemus agrarius*(partial)	--
XM_021659705.1:*Meriones unguiculatus*	--SPEF----------HSQEDKLALGAESKKDENSLKFRVSHELDSSSSEVN
XM_013350872.1:*Microtus ochrogaster*	--SHEF----------HSHEDKLVPGSKSKEDANSLKFHISHEIESSSSEVN
XM_006975432.2:*Peromyscus maniculatus bairdii*	--SHEF----------HSQEDKLVPVSKSKEDTNNLKIYISHEIESSSSEVN
AJ890352.1:*Mesocricetus auratus*	--SH---------------EDKLVPDFKSKEDTNHLKIRISHEIESSSSEVN
HQ647182.1:*Cricetulus barabensis*(partial)	--
XM_007638075.2:*Cricetulus griseus*	--SH---------------EDKLVPDSKSKEDINHLKIRISHEIESSSSEVN
NW_004504357.1:*Jaculus jaculus*	--SQEFHSQELHSQEVHSQEDKLVADPKSREEEKHLKFRISHELDSTSSEVN
XM_020154612.1:*Castor canadensis*	--SHES----------TSLEDKPVLDPKSQKEDKHLKYRISHELDSASSEVN
XM_008830194.2:*Nannospalax galili*	--SHEF----------HSHEDKLVLDPKSKEDDKYLKFRISHELDSASSEVN

Table S1.11: Osteopontin in Rodentia. The available *Chinchilla lanigera* sequence possibly reflects the transcription or translation from an alternative start site. Uniquely, the *Dipodomys ordii* sequence contains a long repetitive insert. Further downstream a repetitive insert is reported only for *Cavia porcellus*.

```
AKHW03002540.1:Alligator mississippiensis    ---------MGLINSHIPGKVNITMKTAVLCLCLIGIAFALPVSKSKHRPDSRSSEERDD
NW_017728948.1:Crocodylus porosus            MIFICRYRTTDCISGFPCSKVNITMKIAVLCLCLIGIAFALPVNKSKHQPDSRSSEERDD
XM_006018107.1:Alligator sinensis            ------MTSTYMLWQKGVIMVNITMKTAVLCLCLIGIAFALPVSKSKHRPDSRSSEERDD
XM_019526454.1:Gavialis gangeticus           ------------------------MKIAVLCLCLIGIAFALPVSKSKHQPDSRSSEERDD
XM_006135415.2:Pelodiscus sinensis           ------------------------MKLAVFCLCLIGMTFALPVSKSNHHAISESSEEKHN
XM_005292014.2:Chrysemys picta bellii       ------------------------MKIAVLCLCLISIAFALPVSKLKRHAISESSEENHD
XM_007066639.1:Chelonian mydas               ------------------------MKIAVLCLCLISIAFALPVSKSKHHAISESSEDNHD
                                                                     ** **:*****.::*****.* :::  *.***:..:

AKHW03002540.1:Alligator mississippiensis    SRSHHHHRHHL-RVQTESRVSPPWAQEVHVLPQQTSFSSEESVEVPKRQIFQVA-SRSTE
NW_017728948.1:Crocodylus porosus            SRSHH--RHHH-HVQIESRVSPPRAQEVHVLPQQTSFSSEESVEVPKRQIFQVA-SRSTE
XM_006018107.1:Alligator sinensis            SRSHHHHRHHH-RVQTESRVSPPWAQEVHVLPQQTSFSSEESVEVPKRQIFQVA-SRSTE
XM_019526454.1:Gavialis gangeticus           SRSHHHHRHHH-RVQIESRVSPPRAQEVHVLPQQTSFSSEESVEVPKRQIFQVA-SRSTE
XM_006135415.2:Pelodiscus sinensis           P--------------------------------------------------SESASVSNE
XM_005292014.2:Chrysemys picta bellii       SRSHHSHRHHHQHVHTQSRVRPAQTQEFLVPPQQDCFSSDESVD-TEQQSLPEPASVSNE
XM_007066639.1:Chelonian mydas               SRSHHSHRHHHQQVHTQSRVRPAKTQELLVPPQQSHFSSEESVENTEQQSLPKPASVSNE
                                                                                                    * *.*

AKHW03002540.1:Alligator mississippiensis    SE-DDHDDDSNDTDESDEDAVTDFPTDFPVTETFPPFSPTRGDNSGRGDSVGYRMRAKAK
NW_017728948.1:Crocodylus porosus            SE-DDQDDDSNDTDESDEDAVTDLPTDFPVTETFPPFPPTRGDNSGRGDSVGYRMRAKAK
XM_006018107.1:Alligator sinensis            SE-DDHDDDSNDTDESDEDAVTDFPTDFPVTETFPPFSPTRGDNSGRGDSVGYRMRAKAK
XM_019526454.1:Gavialis gangeticus           SE-DD--DDSNDTDESDEDAVTDFPTDFPVTETFPPFPPTRGDNSGRGDSVGYRMRAKAK
XM_006135415.2:Pelodiscus sinensis           DDGDHDDNDSNDTDESDENNVTDFPTDAPITTPFTPALPTRGDNSGRGDSVAYRMGAKAK
XM_005292014.2:Chrysemys picta bellii       DDGDHDDNDSNDTDESDEDVVTDFPTDAPVTTPLTPALPTRGDNSGRGDSVAYRMRAKAK
XM_007066639.1:Chelonian mydas               DDGDHDDNDSNDTDESDEDIVTYFPTEAPVTTPLTPAVPTRGDNSGRGDSVAYRMRAKAK
                                             .: *.  :**********: ** :**: *:*  : *  *************.*** ****

AKHW03002540.1:Alligator mississippiensis    LV------KSSRHYQATGKFIVHDATEEGDSTPDTESQQVDYSKPLPAAHLVPGKYDISV
NW_017728948.1:Crocodylus porosus            LV------KSSRHYQATGKFIVHDATEEDDSTPDTESQQVDYSKPLPAAHLVPGKYDISV
XM_006018107.1:Alligator sinensis            LV------KSSRHYQATGKFIVHDATEEGDSTPDTESQQVDYSKPLPAARLVPGKYDISV
XM_019526454.1:Gavialis gangeticus           LV------KSSRHYQATGKFIVHDATEEDDSTPDAESQQVDYSKPLPAAHLVPGKYDISV
XM_006135415.2:Pelodiscus sinensis           LLEVYHKEKPSKLYKSSGKFIVYDVTEEDDSTPDIESQQVDSSKRQPAAHHFPGKSNISM
XM_005292014.2:Chrysemys picta bellii       LADVYHKEKSSKLYKAAGKFIIHEVSEEDASKPETESQQVDSFKAQPAAHRFPGKSDISL
XM_007066639.1:Chelonian mydas               LVDIYHKEKSSKLYKALGKFIVHDVTEEDDSTPDAESQQVDSSKAPCAACHFPGKFDMSM
                                             *       * *: *:: ****:::.:**. *.*: ******  *   **  .*** ::*:

AKHW03002540.1:Alligator mississippiensis    EWDDKSHMKDSNEVISRSRDD-----SHQRLESVEVDTDDSKPDVPEDSHMSNESREQHH
NW_017728948.1:Crocodylus porosus            EWDDKSHMKDSNEVTSRSRDD-----SHQRLESIEVDRDDSNPDVPEDSHMSNESREQHH
XM_006018107.1:Alligator sinensis            EWDDKSHMKDSNEVTSRSRDD-----SHQRLESVEVDRDDSKPDVPEDSHMSNESREQRH
XM_019526454.1:Gavialis gangeticus           EWDDKSHMKDSNEVTSRSRDD-----SHQRLESVEVDRDDSNPDVPENSHMSNESREQHH
XM_006135415.2:Pelodiscus sinensis           KLNDKNNVQGSNEVSSRSHDKSMEKDSQQQLDSVAVDSNNSKSDVTEDSHMSNESTEQQQ
XM_005292014.2:Chrysemys picta bellii       ELDDKSNMQDSNEVNSRSRDKSMENDSQQQLDSVEADSDNSKRDVSEDSHMSNESTEQLR
XM_007066639.1:Chelonian mydas               EWDDRSNIQDISEVNRRSHDKSMENGSQQQLDSVEADSDNSKPDVTEDSHMSNESTEQQQ
                                             : :*:.:::. .**  **:*.     *:*:*:*: .* ::*: ** *:******* ** :
```

```
AKHW03002540.1:Alligator mississippiensis      TQI-----SASVPETDADSNQTAESAEDIQDHHSLEDNEVTI
NW_017728948.1:Crocodylus porosus              TQI-----SASLPETDADSNQTAESVEDIQDHHSLEDNEVTI
XM_006018107.1:Alligator sinensis              TQI-----SASVPETDADSNQTAESAEDIQDHHSLEDNEVTI
XM_019526454.1:Gavialis gangeticus             TQI-----SASVPETDADSNQTAESVEDIQDHHSLEDNEVTI
XM_006135415.2:Pelodiscus sinensis             VQTEYLQQIDDVPEGNDNSNQTSESTEDIQDHNSIEDNEIIL
XM_005292014.2:Chrysemys picta bellii          AQIEDLQQVDGIPEVN-DSNQTSESTEDTQDHKSIEDNEVIL
XM_007066639.1:Chelonian mydas                 AQTEDLQQVDVVPEVN-DSNQTAESTEDTQDHSSIEDNEVIL
                                               .*         :** :  ****:**.** *** *:****: :
```

Table S1.12: Osteopontin in Reptilia (Crocodilia and Testudines).

```
XM_015411327.1:Gecko japonicus                    MKIAVLCLCLFTIAFAIPIKKTKFHDG-AGSSEDNHDSRGHHVHKHRHHHHHSHSQSQEH
XM_020796504.1:Pogona vitticeps                   MKIAILCLCLLTITFAHPVRKSRHHDDGSMSAEGRHDSRGHHHHGHRHH--HHDSHSQEH
XM_003221296.2:Anolis carolinensis                MKIAILCLCLLTIAFALPISKSRHHDD-SGSLERKHEHRHHAHPHHHHR--HHNSQSHEH
XM_007443523.2:Python bivittatus                  MKIAVLILCLFTVAFALPISKSRHHDD-SRSAERKHDSREDEAYAHHSH--HHNSHSHEH
NW_013658771.1:Thamnophis sirtalis                MKIAVLTLCLFTVAFALPISQSRDHDN-SMSAESKHDSKENEAHAHHHH--HHHSHSH--
XM_015819303.1:Protobothrops mucrosquamatus       MKIAVLIVCLFTVALALPISKSTHHDD-SRSAERKHDSRENEAHAH-HH--HHNSHSHEH
GBEX01002350.1:Crotalus adamanteus cadam          MKIAVLIVCLFTVAFALPISKSTHHDD-SRSAERKHDSRENEAHAHHHH--HHNSHSHEH
                                                  ****:* :**:*:::* *:  ::  **. : * * .*: : .    *  :  * .*:*:

XM_015411327.1:Gecko japonicus                    SPEHVNSHE-SQEHQ------LPSHQSSDSDESEEITEQQTLLVHPD--KSAEDDHSDVD
XM_020796504.1:Pogona vitticeps                   SQEHSQEHVHSHESQ--EQQVSSQLSAPSSEESEEITEQQTLLVPAVASKSMEDD----D
XM_003221296.2:Anolis carolinensis                SREHVNSHE-SQ-----EQQVSAQLNSMSSEESDEITEQQTLLVPSS--RSKEDD----H
XM_007443523.2:Python bivittatus                  SHEHRNS----QESQEH---ASSQLNSQSLEDRDEITEQQTLLASSS--KSHEDD----D
NW_013658771.1:Thamnophis sirtalis                --------EHSQESPEHHSQESSQLNSQSSEDRDEITEQQTLLASSS--KSHEDD----D
XM_015819303.1:Protobothrops mucrosquamatus       SQEHGNSHEHSQESPEH---ASSQLNSQSLEDRDEITEQQTLLASSS--KSNEDD----D
GBEX01002350.1:Crotalus adamanteus cadam          SQEHGNSHEHSQESPEH---ASSQLNSQSLEDRDEITEQQTLLASSS--KSNEDD----D
                                                                         . .: . :: :*********.     :* ***    .

XM_015411327.1:Gecko japonicus                    DHDDVDDHDDVDDDSHGEADDSDESHESDDSHQVVTEVPTDRPFTPSAAPGPFTGRGDI-
XM_020796504.1:Pogona vitticeps                   NHSDVDDDD---DDHSD-SDDSDESH-----EGTVTDLPTDSPFTPPVPPDPFTGRGDSA
XM_003221296.2:Anolis carolinensis                DHSDVDDDD---DDND--HTDSHES---DESHEVVTDFPTDAPFTLPVTPDTFTGRGDSA
XM_007443523.2:Python bivittatus                  DHSDASNDD----DHVD----SDES---DESHETFTDFPTDAPFTLPVSPDPFTGRGDVA
NW_013658771.1:Thamnophis sirtalis                DHSDVSKDD----DHSDDSDDSSES---DESHETVTDAPTEAPYT-----ESVTNRGDVP
XM_015819303.1:Protobothrops mucrosquamatus       NHSDVSKDD----DHPD----SDES---DESHENFTDFPTQAPVTF----DSVTYRGDVA
GBEX01002350.1:Crotalus adamanteus cadam          NHSDVSKDD----DHPD----SDES---DESQENFTEYPTQAPVTL----DSVTYRGDVA
                                                  :*.*....*    *. .    * **   .  .  .*: **: * *       .* ***

XM_015411327.1:Gecko japonicus                    PYGIQHGQ----LKFLRKSKKSSEQFDSHDTTQEDDSLPDVDSHETHSSKAVSHESQDTS
XM_020796504.1:Pogona vitticeps                   PNGIRAKVDLVHFKK---SYKKSGKFDSHDVTDDFDSTPDVESHESYSSKAHSLESHDTS
XM_003221296.2:Anolis carolinensis                PYGLKSRVDLVHFEKSKKVYKKSGKFDSHDVTDDFDSSPDVESHESYSSKAHSTESHDTS
XM_007443523.2:Python bivittatus                  PYGIRAKLDRMSLGKSGKAHKKSGKFDSQDFSDEVDSTPDEDSHEY--SKARSLESHDTS
NW_013658771.1:Thamnophis sirtalis                SDEFRPNVDFMSLGRS-------GKFDSQSVSDEVDSTPDDESHED--SKARSLESHE--
XM_015819303.1:Protobothrops mucrosquamatus       PYGFGAKDDQMSLERS-------GKFDSQDVSDEVDSTPDDESHEY--AKARSLESHE--
GBEX01002350.1:Crotalus adamanteus cadam          PYGFRAKDDQMSLERS-------GKFDSQDVSDEVDSTPDDESREY--AKARSLESHE--
                                                     :        :           :***:. ::: ** ** :*:*   :** * **::

XM_015411327.1:Gecko japonicus                    TESEDKSNLQDSNEVHSASHDKSVENDSRQKLDSVEEDH-----DNSKADISQESDGIDH
XM_020796504.1:Pogona vitticeps                   VE----SDLRESNEVHSTSHDRSLERASQQTLESVEDDQDDADHDNSKITASLESHE---
XM_003221296.2:Anolis carolinensis                VEKDDASHLRDSNEVLRASRDKSLENNSRQKLESAEYDHDDADHDNIKLAIALASHE---
XM_007443523.2:Python bivittatus                  VERVDKSNLRDSNEVHSASHERSLEDNSQEKPESVEDS-----HDNSKVTISLESTE---
NW_013658771.1:Thamnophis sirtalis                --SDDKSDLTDSNEVHSASHDISLEDHSPEKPESVEDS-----SDHSKASLSLESTE---
XM_015819303.1:Protobothrops mucrosquamatus       --GDDKSNLRDSNEVHSASHDTSLEDHSPEKAESVEDS-----HDHSKVSVSLESTE---
GBEX01002350.1:Crotalus adamanteus cadam          --RDDKSNLRDSNEVHSASHDTSLEDHSPEKPESVEDS-----HDHSKVSVSLESTE---
                                                        *.* :****  :*:: *:*  * :. :*.* .      *: *   :  *
```

```
XM_015411327.1:Gecko japonicus                ITELLKARRGEPQEADHDHDVDND------------------------------------
XM_020796504.1:Pogona vitticeps               STELQKDYHDGLQQVAHDRSIDDVSNQTLESTEDHHSVEDRHSAEDKHSVEDH-SIENHH
XM_003221296.2:Anolis carolinensis            SREYLKDRSVELRQVDHDHDVDDVSNQSLENVEDHHSVEDHHSVEDHHSIEDHHSVEDKH
XM_007443523.2:Python bivittatus              SREEKKDRSEELQQLEP--DVDDVSNQTLESTESHHSVEDHHSAEDHHSAEDHHSAEDHH
NW_013658771.1:Thamnophis sirtalis            SREQKKDHSEEPQQLDS--NPDDASNQTVESAESQHSS----------------------
XM_015819303.1:Protobothrops mucrosquamatus   SGEQKKDHSEELQQLDS--NPDDVSNQSLESAENHHSA----------------------
GBEX01002350.1:Crotalus adamanteus cadam      SGEQKKDHSEELQQLDS--NPDDVSNQSLESTENHHST----------------------
                                                *  *      ::     . *:           :**

XM_015411327.1:Gecko japonicus                ----------------------HHSVED------NEIEY
XM_020796504.1:Pogona vitticeps               SVEN-------------HSTEDHHSVEDHHSIEDNEVTL
XM_003221296.2:Anolis carolinensis            SVEDKHSVEDHHSIEHPLSVEDHHSVEDHHSIEDNEVTL
XM_007443523.2:Python bivittatus              --------------------EDHHSAEHHDSVEDNEVIL
NW_013658771.1:Thamnophis sirtalis            --------------------EDHHSVEHHDSVEDNEVIL
XM_015819303.1:Protobothrops mucrosquamatus   --------------------EDHHSVEHHDSVEDNEVIL
GBEX01002350.1:Crotalus adamanteus cadam      --------------------EDHHSVEHHDSVEDNEVIL
```

Table S1.13: Osteopontin in Reptilia (Squamata).

M59182.1:*Gallus*	MKLAFLCLCFISIAAAWPVSKSRQHAISASSEEKYDPRSHHTHRYHQ-----DHVDSQSQ
XM_003205565.3:*Meleagris gallopavo*	MKLALLCLCFISIAAAWPVSKSKQHAISASSEEKYDPRSHHAHRYHQ-----DHVDSQSQ
XM_021395699.1:*Numida meleagris*	MKVALLCLYFISIAAAWPVSKSKQHAISASSEEKYDPRSHHAQRYHQ-----DHVDSQSQ
NM_001323234.1:*Coturnix japonica*	MKLTLLCLCFISITAAWPVSKSKQHAISASSEEKYDPRSHHTHRYHQ-----DHVDSQSQ
XM_010218705.1:*Tinamus guttatus*	MKVAVLCLCFISITIAWPLSKSKQHAVSASSEEKYDARGHQSHRHH-------HVTSQSL
XM_009676586.1:*Struthio camelus australis*	MKVAVLCLCLISITVAWPVSKSKQHAVSASSEEKYDSRGHHSHRHHH-----RHVNSHSQ
XM_013957376.1:*Apteryx australis mantelli*	MKVAVLCLCLISITVAWPVSKSKRHAISASSEEKYDSRGHRSHRQHH-----HHVNSQSH
XM_021526515.1:*Lonchura striata domestica*	MKVAVLCLCLISITAAWPVVPSKQHAISASSEEKYDSRGQHLHRYHN-----DHVNSQSQ
XM_002190366.1:*Taeniopygia guttata*	MKVAVLCLCLISITAAWPVVPSKQHAISASSEEKYDSRGQHLHRYHN-----DHVNCQYQ
NW_008240042.1:*Corvus brachyrhynchos*	MKVAVLCLCLISITAAWPVIQSKHHAISASSEEKYDSRSHHLRRYHN-----DHMNSQSQ
XM_010412044.2:*Corvus cornix cornix*	MKVAVLCLCLISITAAWPVIQSKHHAISASSEEKYDSRSHHLRRYHN-----DHMNSQSQ
XM_015623543.2:*Parus major*	MKEAILCLCLISITAAWPVIQPKQHAISASSEEKYDSRGHHLHRYHN-----DQENSQSQ
XM_005525626.2:*Pseudopodoces humilis*	MKEAVLILCLISITAAWPVIQSKQHAISASSEEKYDSRGHHLHRYHN-----DHENSQSQ
XM_014878941.1:*Sturnus vulgaris*	MKVAVLCLCLISITAAWPVIQSKQHAISASSEEKYDSRGHHFHRYHD-----YHVNSQSQ
XM_016297843.1:*Ficedula albicollis*	MKVAVLCLCLISITAAWPVIQSKQHAISASSEEKYDSRGHHLRRYYD-----DHVNSHSQ
NW_007931124.1:*Serinus canaria*	MKETVLCLCLISITAAWPVIQSKQPVISASSKEKYDSRGHHLHRYHN-----DYVNSQSW
XM_005418195.1:*Geospiza fortis*	MKVVVLCLCLISITAAWPVIQSKKHAISASSKEKYDSRGHHLHKYHN-----DYVNSQSQ
XM_005485296.1:*Zonotrichia albicollis*	-MKVVLCLCLISITAAWPVIQSKQHAISSSSKEKYDSRGHHLHKYHN-----DYVNSQSQ
XM_009904975.1:*Picoides pubescens*	MKAAVLCLCLLSLAAAWPVHKSKQQATSASSEEKYDPKGHYSHRYKH----------HSW
XM_005012779.3:*Anas platyrhynchos*	MKVAVLCLCLISVAAAWPI-KSKQHAISASSEEKYDTRGHHSHRQQH-----NLVNSHSQ
XM_013190510.1:*Anser cygnoides domesticus*	MKVTVLCLCLISVAAAWPI-KSKQHAISASSEEKYDPRGHHSHRQHH-----DLVNSHSQ
KZ506572.1:*Limosa lapponica baueri*	----------MVARSLMTSSFSATWGLVAVPGTGFKFPEVGDVRQSGDEGRKPELLGINW
XM_005498492.2:*Columba livia*	MKVAVLCLCLISIVAAWPVSKSKQHAVSGSSEEKYDPRSHHLHRQHH-----KHVNSQSW
LSYS01006159.1:*Patagioenas fasciata monilis*	MKVAVLCLCLISIVAAWPVSKSKQHAVSGSSEEKYDPRGHHSHRQHH-----KHVNSQSW
XM_008500834.1:*Calypte anna*	MKVEVLYLCLISIAAARPVNKSKQHAVSASSEEKYDPRGHHSHRYQH------HMNSQSW
XM_009993754.1:*Chaetura pelagica*	MKVAVLCLCLISITAAWPVNKSKQHATSASSEEKHDPRGHHSHRHHH-----HQVNPQSW
XM_008942236.1:*Merops nubicus*	MKVAVLCLCLTSIAAAWPVSKPKQHAVSASSEEKYDPRGHHSYKYQ------HHVNSQSW
XM_010019624.1:*Nestor notabilis*	MKVAVLCLCLISITAAWPVSKSKQHAISASSEEKYDTKGHHSRRYHQ-----HHVNSQSW
XM_005146580.1:*Melopsittacus undulatus*	MKVAVLCLCLISIAAAWPVSKTKQHAISASSEEKYDPKAHHSHRYH-------HVNSQSW
XM_009559526.1:*Cuculus canorus*	MKVAVLCLCLISIVTAWPVNKSKKHAISASSEEKYDLKGHSSHRHYH-----HHVNSQSW
XM_005444503.1:*Falco cherrug*	MKVAVLCLCFISITAAWPVSKSKHHAISASSEEKYDARGHHSHRYRH-----HHVNSQSW
XM_005233595.1:*Falco peregrinus*	MKVAVLCLCFISITAAWPVSKSKHHAISASSEEKYDARGHHSHRYRH-----HHVNSQSW
XM_009073853.1:*Acanthisitta chloris*	MKVAVLCLCLISITTAWPVRKFKQHATSASSEEKYDSRGHKLHRYHH-----DHVNSQSW
XM_008930247.2:*Manacus vitellinus*	MKVAVLCLCLISITAAWPVNKSKQHAISASSEEKYDPRGHHLHRYHH-----DHVNSQSW
NW_016690259.1:*Lepidothrix coronata*	MKVAVLCLCLISITAAWPVSKSKQHAISASSEEKYDPRGHHLHRYHH-----DRVNSQSW
XM_010202192.1:*Colius striatus*	MKVAVLCLCLIGITAAWPVSKFNQHAISASSEEKYDPRGHDSHRYQH-----HHANSQSW
XM_010173470.1:*Caprimulgus carolinensis*	MKVAVLCLCLISITAAWPVSKSKQHGVSASSEEKYDHRGHHSHRYHH----HHHVNSQSW
XM_010181850.1:*Mesitornis unicolor*	MKVAVLCLCLISVAAAWPVSKSKQHAISASSEEKYDLRGHQLHRHHP-----HHVNSQSW
XM_009868641.1:*Apaloderma vittatum*	MKAAVLCLCLISITAAWPVSKSKQHAISASSEEKYDARGHHSHRYHP----HHDVNSQSW
XM_009958978.1:*Leptosomus discolor*	MKVAVLCLCFISITTAWPVSKSKQHAISASSEEKHDARGHHSHRYHH-----QHVNSQSW
XM_010079868.1:*Pterocles gutturalis*	MKVAVLCLCLVSIAAAWPVSKSKQHAISASSEEKYDPRGHHSHRHHH-----HHVNSQSW
XM_010119303.1:*Chlamydotis macqueenii*	MKVAVLCLCIISVAAAWPVGKSKQHATSASSEEKYDPRSHLSHRHY-----HHHVNSQSW
XM_009970430.1:*Tyto alba*	MKLAVLCLCLISITDAWPVSKSKQHAFSASSEEKYDPRGHHSHRYH------HHMNSRSW
XM_009932280.1:*Opisthocomus hoazin*	MKVTVLCLCLISITAAWPVSKSKQHAISVSSEEKSDARGHQTHRYHH-----HHRNSQSW
XM_010145609.1:*Buceros rhinoceros silvestris*	MKVAVLCLCLISITAAWPVSKSKQHAISASSEEKHDPRGHHSHRYHH-----RSVNSQSW
XM_009978083.1:*Tauraco erythrolophus*	MKVVVLCLCLTSITVAWPVSKSKQHAISASSEEKYDPRGHHSHRYH------HHVNSQSW
XM_010154680.1:*Eurypyga helias*	MKVAVLCFCLISITAAWPVSKSKQHAISTSSEEKYDPRGHHSHRYHHD---HHDVNSQSW
XM_009639993.1:*Egretta garzetta*	MKVAVLCLCLISITAAWPVSKSKQHAISASSEEKYDPRGHHSHRYHH-----HHVNSQSW
XM_009327481.1:*Pygoscelis adeliae*	MKVAVLCLCLISITAAWPVSKSKKHAISASSEEKYDPRGHHSHRYHH-----HHVNSQSW
XM_009280227.1:*Aptenodytes forsteri*	MKVAVLCLCLISITAAWPVSKSKKHATSASSEEKYDPRGHHSHRYHHHHHHHHVNSQSW
XM_009512482.1:*Phalacrocorax carbo*	MKVAVLCLCLISITAAWPVSKSKQHAISASSEEKYDSKDHHSNRYHH-----HHVNSQSW
XM_014961493.1:*Calidris pugnax*	MKVAVLCLCLISITTAWPVSKSKQHAISASSEEKYDPRGHHSHRYHHHP--HHHVNSQSW
XM_009813620.1:*Gavial stellata*	MKVAVLCLCLISITAAWPVSKSKQHAISASSEEKYDPRGHHSRRYHH--H-HHHVNSQSW
XM_009916485.1:*Haliaeetus albicilla*	MKAAVLCLCLISITAAWPVSKYKQHAVSASSEEKYDPRGHHSHRYHH-----HHMNSQSL
XM_010569846.1:*Haliaeetus leucocephalus*	MKAAVLCLCLISITAAWPVSKYKQHAVSASSEEKYDPRGHHSHRYHH-----HHMNSQSL
XM_011600806.1:*Aquila chrysaetos canadensis*	MKAAVLCLCLISITAAWPMSKYKQHAISASSEEKYDPRGHHSHRYHH-----HHMNSQSW
XM_009490811.1:*Pelecanus crispus*	MKVAVLCLCLISITTAWPVNKSKQHAISASSEEKYDPRGHHSHRYH------HQVNSQSR
XM_009694723.1:*Cariama cristata*	MKVAVLCLCLISITAAWPVNKSKQRAISASSEEKCDSRGHRSHRYHHHD---HHVNSQSW
XM_010311221.1:*Balearica regulorum gibbericeps*	MKVAVLCFCLISIAAAWPVSKSKQHAISASSEEKYDTRGHHSHRYHH-----HHVNSQSW
XM_010287426.1:*Phaethon lepturus*	MKVAVLCLCLVSITVAWPVSKSKQHAISASSEEKYDPRGHHSHRYHHHRR-HHHVNSQSW
XM_009890748.1:*Charadrius vociferus*	MKVAVLCLCLISITAAWPVSKSKQHAISASSEEKYHPRGHHSHRYQH--H-HHHVNSQSW
XM_009584852.1:*Fulmarus glacialis*	MKVAVLCLCLISVTVAWPVSKSKQHAISASSEEKYDPRGRHSYRYHH-----HHVNSQSW
XM_009467655.1:*Nipponia nippon*	MKVAVLCLCLISITAAWPVSKSKQHAISASSEEKYDPRGHYSHRYRH-----HHVNSQSW

M59182.1:*Gallus*	EHLQQT-------QNDLASLQQTHYSSEENADVPEQPDFPDIPSKSQEAVDDDD---DGD
XM_003205565.3:*Meleagris gallopavo*	EHLQQT-------RNDLASLQQTHYSSEENADVPEQPDFPDIPSKSQEAVDDND----DD
XM_021395699.1:*Numida meleagris*	EHLQQS-------QNDLASLQQTLYSSEENADVPEQPDFPDIPSKSHEAVDDDD---DDN
NM_001323234.1:*Coturnix japonica*	EHLQQT-------QNDLASLQQTHYSSEENADVPEQPDFPDVPSKSQETVDDDD---DDD
XM_010218705.1:*Tinamus guttatus*	ENLLLP-------REDLALPQKTLYSSEER-ADVLVPERFPAVFSKSHEDPDDD---VDD
XM_009676586.1:*Struthio camelus australis*	ESLLHP-------RDDLASPQQTLRSSEEK-AVVPVGLRFTDVTSKSHEDPDDD---VDD
XM_013957376.1:*Apteryx australis mantelli*	ESLLRP-------RDDLASPQQTLYSSEER-ADVLVHPRFPGLLSKSREDPDDD---VDD
XM_021526515.1:*Lonchura striata domestica*	ESQQHP-------QSDLASSQQTLYSSEES-MDVPEQLHFPDVSSKSHEDVDDDDD-DDD
XM_002190366.1:*Taeniopygia guttata*	ESQQHP-------QSDLASSQQTLYSSEES-MDVPEQLHFPDVSSKSHEDVDDDDD---D
NW_008240042.1:*Corvus brachyrhynchos*	ESQQHP-------QSDLASSQQTLHSSEES-MDVPVQLHFPDVSSKSHEDVDDDDD-DDD
XM_010412044.2:*Corvus cornix cornix*	ESQQHP-------QSDLASLQQTLHSSEES-MDVPVQLHFPDVSSKSHEDVDDDDD-DDD
XM_015623543.2:*Parus major*	ESQQHP-------QSDLASSQQTLYSSEES-VDVPEQLHFPDVSSKSHEDVDDDDD-DDD
XM_005525626.2:*Pseudopodoces humilis*	ESQQHP-------QSDLASSQQTLYSSEES-VDVPEQLHFPDVSSKSHEDVDDDDD-DDD
XM_014878941.1:*Sturnus vulgaris*	ESQQHP-------QSDLASSQQTLYSSEES-VDVPEQQHFPDVSSKSHEDVDDDDD-DDD
XM_016297843.1:*Ficedula albicollis*	ESQQHP-------QSDLASSQQTLYSSEES-VDVPEQPHFPDVSSKSHEDVDDDDD-DDD
NW_007931124.1:*Serinus canaria*	ESQQHP-------QSDLASSQQTLYSSEES-VDVPEQLHFSDVSSKSHEDVDDDDD-DDD
XM_005418195.1:*Geospiza fortis*	ESQQDP-------QSDLASSQQTLYSSEES-VDVPEQLHFPDVSSKSHEDVDDDDDDDDD
XM_005485296.1:*Zonotrichia albicollis*	ESQQHP-------QSDLASSQQTLYSSEES-VDVPEQLPFPDVSSKSHEDVDDDDD-DDD
XM_009904975.1:*Picoides pubescens*	ESLQHP-------QPDLVLPQQTLYSSEES-VDVSV-HQIPDVPSTSQEDADGGDDDDDD
XM_005012779.3:*Anas platyrhynchos*	ELLQHP-------QNDLASLQQTLYSSEENAENVPVKPHFPDVPSKSHEVVDDDNDD-DD
XM_013190510.1:*Anser cygnoides domesticus*	ELLQHP-------QNDLASLQQTLYSSEENAENVPVKPHFPDVPSKSHEAVDDDNDDDDD
KZ506572.1:*Limosa lapponica baueri*	PELLLGSLRALLLQLTPPAPEKTLHSSEEY-VDDPVQPHFPDVSSKSHEDVDDD---DDD
XM_005498492.2:*Columba livia*	ESLQHT-------QNDL--AQQTLDSSEES-VDVPVQAHFPVVSSKSHEDVDGDD-----
LSYS01006159.1:*Patagioenas fasciata monilis*	ENLQHT-------QNDL--AQQTLHSSEES-VDVPVQPHFPVVSSKSHEDVDDDD-----
XM_008500834.1:*Calypte anna*	ETLESPQ------QXGLC-CLQTLYSSEES-TDILGQPHFPDVSSKSHEDVKDDD--GGN
XM_009993754.1:*Chaetura pelagica*	ESLQQP-------QDDLVSPQQALYSSEES-MDVPVQPDFPSVSSKSHEDTEDDD--NDD
XM_008942236.1:*Merops nubicus*	ESLQHES------QDDLESPQQTLYSSEES-VDVPVQTHAPDVSSKSHEDVDDN----DD
XM_010019624.1:*Nestor notabilis*	ESLQQP-------QNDLVSPQQTLYSSEES-VDVPVQPQFPDVSSKSHEDVDDD---DDD
XM_005146580.1:*Melopsittacus undulatus*	ESLQQP-------QNDLASPQQTLYSSEES-VDVPEQPQFPDVSSKSHEDVDDD---DDD
XM_009559526.1:*Cuculus canorus*	ESLQHP-------QNDLASPQQTLYSSEES-VDVPEQPHFPDVSSKSHEDVDD-----DD
XM_005444503.1:*Falco cherrug*	ESLEHP-------QNDLASPQQTLYSSEES-VDLPVKPHFPDVSSKSHEDVDDDDD-DDE
XM_005233595.1:*Falco peregrinus*	ESLEHP-------QNDLASPQQTLYSSEES-VDLPVKPHFPDVSSKSHEDVDDDD--DDE
XM_009073853.1:*Acanthisitta chloris*	ESQQHS-------QDDLASPQQTLYSSEES-VDDPAQPQFPDVSSKSHEDVDDDN--DDD
XM_008930247.2:*Manacus vitellinus*	ESQQHT-------QTDLASPQQTLYSSEES-VDVPEEPHFPDVSSKSHEDVNDNDDDDDD
NW_016690259.1:*Lepidothrix coronata*	ESQQHT-------QTDLASPQQTLYSSEES-VDVPVEPHFPDVSSKSHEDVNDND--DDD
XM_010202192.1:*Colius striatus*	ESMKHP-------QNDLASPRQTLYSSEES-VDVPAQPNFPDVSSKSHEDADDD----DD
XM_010173470.1:*Caprimulgus carolinensis*	ESLQDS-------QNDLASPQQTLYSSEES-VDVPVKPHFPDVSSKSHEDVDDD--DDDD
XM_010181850.1:*Mesitornis unicolor*	ESLQHA-------RNDLASPQQTLYSSEES-VDVPEQPRFPDVSSKSHEDVADD---DDD
XM_009868641.1:*Apaloderma vittatum*	ESLQHA-------QNDLASPQQTLYSSEES-VDVAVQPHFADVSSKSHEDVDDD---DDD
XM_009958978.1:*Leptosomus discolor*	ESLQHT-------ENDLASPQQTLYSSEES-VDVPAKLHFADVSSKSHEDVDDD---DDD
XM_010079868.1:*Pterocles gutturalis*	ERLQHA-------QNDLPSPQQTLYSSEES-VDVPVQPQFPDVSSKSHEDVDDD---DDN
XM_010119303.1:*Chlamydotis macqueenii*	ERLQHS-------QNDVASPQQTLYSSEES-VDVPVKPHFPDVSSKSHEDVDDD---DDD
XM_009970430.1:*Tyto alba*	ESLQHP-------QNDLASPQQTLYSSEES-VDVPVQPHFPDMPSKSHEDVDDD---DDD
XM_009932280.1:*Opisthocomus hoazin*	EGLHHP-------QNDLASPQQTLYSSEES-VDVPVQPHFPDVSSKSHEDVDDD----DD
XM_010145609.1:*Buceros rhinoceros silvestris*	ERLQHP-------QNDLVSPQQTLYSSEES-VDVPEQPHFPDVSSKSHEDVDDD---DDD
XM_009978083.1:*Tauraco erythrolophus*	ESLQHP-------QNDLASPQQTLYSSEES-VDVPVQPHFPDVSSKSHEDVDDD---DDD
XM_010154680.1:*Eurypyga helias*	ESLQHP-------QNDLASPQQTLYSSEES-AGVPVQPHFPEVSSKSHEDVDDD---NDD
XM_009639993.1:*Egretta garzetta*	ERLQHP-------QNDLASPQQTLYSSEES-VDAPVQPHFPDVSSKSQEDVDDD---NDD
XM_009327481.1:*Pygoscelis adeliae*	ESLQHS-------QNDLASPQQTLYSSEES-VDVPVQPHFPDVSSKSHEDVDDD----DD
XM_009280227.1:*Aptenodytes forsteri*	ESLQHP-------QNDLASPQQTFYSSEES-VDVPVQPHFPDVSSKSHEDVDDD----DD
XM_009512482.1:*Phalacrocorax carbo*	ESLQHP-------QNDLASPQQTLYSSEES-MDVPVKPQFPDVSSKSHENVDDD---DDD
XM_014961493.1:*Calidris pugnax*	ESLQHP-------QNDLASPQQTLHSSEEY-VDDPVQPHFPDVSSKSHEDVDDD---DDD
XM_009813620.1:*Gavial stellata*	ESLQHP-------QNDLASPQQTLYSSEEN-VDVPVQPHFPDVSSKSHEDVDDD---DDD
XM_009916485.1:*Haliaeetus albicilla*	ESLQHT-------QNDLMSPQQTLYSSEES-VDVPVQPHFPDVSSKSHEDVDDD---NDD
XM_010569846.1:*Haliaeetus leucocephalus*	ESLQHT-------QNDLMSPQQTLYSSEES-VDVPVQPHFPDVSSKSHEDVDDD---NDD
XM_011600806.1:*Aquila chrysaetos canadensis*	ESLQHT-------QNDLMSPQQTLYSSDES-VDVPVQPHFPDVSSKSHEDVDDD---NDD
XM_009490811.1:*Pelecanus crispus*	ESLQHP-------QNDLESPQQTLYSSEES-VDVPVQPHFPEVSSKSHEDVDDD---NDD
XM_009694723.1:*Cariama cristata*	ESLQHS-------QNDLASPQQTLYSSEES-VDVPVQPHFPDVSSKSHEDVDDD---DDD
XM_010311221.1:*Balearica regulorum gibbericeps*	ESLQHP-------QNDLVSPQQTLYSSEES-VDVPVQPRFPDVSSKSHEDVDDD---DND
XM_010287426.1:*Phaethon lepturus*	ESLQHP-------QNDLASPQQTLYSSEES-MDVPVQPHFPDVSSKGHENMDDD---DDD
XM_009890748.1:*Charadrius vociferus*	ESLKHP-------QNDLASPQQTLYSSEES-MDVPVQPHFPDVSSKSHEDVDDD---DDD
XM_009584852.1:*Fulmarus glacialis*	ESL-HP-------QNDLASPQQTLYSSEES-MDVPVQPHFPDVSSKSHEDVDDD---DDD
XM_009467655.1:*Nipponia nippon*	ESLQHP-------QNDLASPQQTLYSSEES-VDVPVQPHFPDVSSKSHEDVDDD---DDD

```
M59182.1:Gallus                                          NDSNDTDESDEVVTDFPTEAPV--TPF---------NRGDNAGRGDSVAYGFRAKA----
XM_003205565.3:Meleagris gallopavo                       NDSNDTDESDEVVTDFPTEAPV--TPF---------NRGDNAGRGDSVAYGFRAKA----
XM_021395699.1:Numida meleagris                          NDSNDTDESDEVVTDFPTEAPV--TPF---------NRGDNAGRGDSVAYGFRAKA----
NM_001323234.1:Coturnix japonica                         NDSNDTDESDEVFTDFPTEAPV—-APF---------NRGDNAGRGDSVAYGFRAKA----

XM_010218705.1:Tinamus guttatus                          NDSNDADESDEVVTVFPTDAPVT-AAFPPYPF----TRGDNAGRGDSVAYRIKAEP----
XM_009676586.1:Struthio camelus australis                NDSDDTDESDEVVTNFPTDTPVT-APFPPYPF----TRGDNAGRGDSVAYRIKAKA----
XM_013957376.1:Apteryx australis mantelli                DDSNDTDESDEVVTDFPTDAPVT-APFPPYPF----TRGDNSGRGDSVAYRIKAKA----

XM_021526515.1:Lonchura striata domestica                DDSNDTDESEEVVTSFPTDIPVT-EPFPTFPF----TRGDNAGRGDSVAYRMRAKA----
XM_002190366.1:Taeniopygia guttata                       NDSNDTDESEEVITSFPTDIPVT-EPFPTVPF----TRGDNAGRGDSVAYRMRAKA----
NW_008240042.1:Corvus brachyrhynchos                     NDSNDTDESEEVVTSFPTDIPVT-EPFPTFPF----TRGDNAGRGDSVAYRMRAKA----
XM_010412044.2:Corvus cornix cornix                      NDSNDTDESEEVVTSFSTDIPVT-EPFPTFPF----TRGDNAGRGDSVAYRMRAKA----
XM_015623543.2:Parus major                               NDSNDTDESEEVVTSFPTDIPVT-EPFPTFPF----TQGDNAGRGDSVAYRMKAKA----
XM_005525626.2:Pseudopodoces humilis                     NDSNDTDESEEVVTSFPTDIPVT-EPFPTFPF----TQGDNAGRGDSVAYRMKAKA----
XM_014878941.1:Sturnus vulgaris                          NDSNDTDESEEVATGFPTEIAVTVEPFPTFPF----TRGDNAGRGDSVAYRMRAKAALLR
XM_016297843.1:Ficedula albicollis                       NDSNDTDESEEVVTSFPTDIPVTVEPFPTFPF----TRGDNAGRGDSVAYRMRAKAALLT
NW_007931124.1:Serinus canaria                           NDSNDTDESEEIVTIFPTDIPVT-EPFPTFPF----TQGNNAGRGDSVAYRMRAKA----
XM_005418195.1:Geospiza fortis                           NDSNDTDESEEVVTIFPTDIPIT-EPFPTFPF----TRGDNAGRGDSVAYRMRAKA----
XM_005485296.1:Zonotrichia albicollis                    NDSNDTDESEEVVTIFPTDIPVT-EPFPTFPF----TRGDNAGRGDSVAYRMRAKA----

XM_009904975.1:Picoides pubescens                        DDSNDTEESQEVTTIFPTDIPLT-FP--PS------TQSDTAGRGDSVAYRLRAKA----
XM_005012779.3:Anas platyrhynchos                        NDSNDTDESDEVVTDFPTDAPVT-VPFPPFPF----TRGDNAGRGDSVAYRMKAKA----
XM_013190510.1:Anser cygnoides domesticus                NDSNDTDESDEVVTDFPTDIPVT-APSPPFPF----TRGDNAGRGDSVAYRMKAKA----
KZ506572.1:Limosa lapponica baueri                       NDSNDTDESDEVVTSFPTDIPVT-VPFPPFPF----TRGDNTGRGDSVAYRVRPKA----
XM_005498492.2:Columba livia                             ---DDTDESDEDDDGFPTDVPVT-VPFSPFTRGDSAGRGDSAGRGDSVAYAVRAKA----
LSYS01006159.1:Patagioenas fasciata monilis              ---NDTDESDEVDDSFPTDVPVT-VPFSPFTRGDSAGRGDSAGRGDSVAYVVRAKA----
XM_008500834.1:Calypte anna                              NDSNDTDESDEAVTSLPTDIAVT-VPFPPFPF----TRGDNAGRGDSVAYRVRAKA----
XM_009993754.1:Chaetura pelagica                         SDSNDTDESDEVTTGFPTDIPVT-VPFPPFPF----TRGDNAGRGDSVGYRVRAKA----
XM_008942236.1:Merops nubicus                            NDSNDTDESDEVVTSFPTDIPVT---FPPFPF----SRGDNAGRGDSVAYRARAKA----
XM_010019624.1:Nestor notabilis                          NDSNDTDESDEVVTSFPSDIPVT-VPFPPFPF----TRGDNAGRGDSVAYRVRAKA----
XM_005146580.1:Melopsittacus undulatus                   NDSNDTDESDEAVTSLPTDIPVT-APFPPFPF----TRGDNAGRGDSVAYRMRARA----
XM_009559526.1:Cuculus canorus                           NDSNDTDESDEVVTGFPTDIPVT-VPFHP--F----TRGDNAGRGDSVAHRVRAKP----
XM_005444503.1:Falco cherrug                             DDSNDTDESDEVVTSFPTEIPVT-VPFPP--F----TRGDNAGRGDSVGYRVRA------
XM_005233595.1:Falco peregrinus                          DDSNDTDESDEVVTSFPTEIPVT-VPFPP--F----TRGDNAGRGDSVGYRVRA------
XM_009073853.1:Acanthisitta chloris                      NDSNDTDESDEVVTGFPTDIPVT-VP-----F----TRGDNAGRGDSVAYRLKAKA----
XM_008930247.2:Manacus vitellinus                        NDSNDTDESDEVVTGFPTDIPVT-VPFPPFPF----TRGDNAGRGDSVAYRMRAKA----
NW_016690259.1:Lepidothrix coronata                      NDSNDTDESDEVVTGFPTDIPVT-VPFPPFPF----TRGDNAGRGDSVAYRMRAKA----
XM_010202192.1:Colius striatus                           NDSNDTDESDEVVTGFPTDIPVT-VPFPPFPF----TWGDQAGRGDSVAYRVRAKA----
XM_010173470.1:Caprimulgus carolinensis                  NDSNDTDESDEAVSSFPTEIPIT-VPMPP--F----TRGDNAGRGDSVAYRVRAKA----
XM_010181850.1:Mesitornis unicolor                       NDSNDTDESDEVVTSFPTDIPVT-ASFPPFPF----TRGDNAGRGDSVAYRVRAKA----
XM_009868641.1:Apaloderma vittatum                       NDSNDTDESDEVVTSFPTDIPIT-VPFPP--F----TRGDNAGRGDSVAYRVRAKA----
XM_009958978.1:Leptosomus discolor                       NDSNDTDESEEVVTSFPTDIPVT-VPFPP--F----TRGDNIGRGDSVAYRVRAKA----
XM_010079868.1:Pterocles gutturalis                      NDSNDTDESDEEVTSFPTDIPVT-APFPPFPF----TRGDNAGRGDSVAYRVRAKA----
XM_010119303.1:Chlamydotis macqueenii                    NDSNDTDESDEVVTTFPTDIPVT-APFPPFPF----TRGDNAGRGDSVAYRLRAKA----
XM_009970430.1:Tyto alba                                 NDSNDTDESDEVVTSFPTDIPVT-VPFPP--F----TRGDNAGRGDSVAYRVRAKA----
XM_009932280.1:Opisthocomus hoazin                       NDSSDTDESDEVVTSFPTDIPVT-VPFPPFPF----TRGDNAGRGDSVAYRVRPKA----
XM_010145609.1:Buceros rhinoceros silvestris             DDSNDTDESDEVVTNFPTDIPVT-FP----PF----TRGDNVGRGDSVAYRVRAKA----
XM_009978083.1:Tauraco erythrolophus                     NDSNDTDESDEDVTGFPTDIPVT-VPFPPFPF----TRGDNAGRGDSVAYRARAKA----
XM_010154680.1:Eurypyga helias                           NDSNDTDESDEVVTSFPTDIPVT-VPFPPFPF----TRGDNAGRGDSVAYRMRAKA----
XM_009639993.1:Egretta garzetta                          NDSNDTDESDEVVTNFPTDIPVT-VPFPPFPF----TRGDNSGRGDSVAYRVRAKA----
XM_009327481.1:Pygoscelis adeliae                        NDSNDTDESDEVVTSFPTDIPVT-VPFSPFPF----TRGDNAGRGDSVAYRVRAKA----
XM_009280227.1:Aptenodytes forsteri                       NDSNDTDESDEVVTSFPTDIPVT-VPFSPFPF----TRGDNAGRGDSVAYRVRAKA----
XM_009512482.1:Phalacrocorax carbo                       NDSDDTDESDEVATSFPTDIPVT-VPFPPSPF----TRGDNAGRGDSVAYRVRAKA----
XM_014961493.1:Calidris pugnax                           NDSNDTDESDEVVTSFPTDIPVT-VPFPPFPF----TRGDNTGRGDSVAYRVRPKA----
XM_009813620.1:Gavial stellata                           NDSSDTDESDEVVTSFPTDIPVT-VPFPPFPF----TRGDNAGRGDSVAYRVRAKV----
XM_009916485.1:Haliaeetus albicilla                      NDSNDTDESDEVVTSFPTDIPVT-VPFPPFPF----TRGDNSGRGDSVAYRVRAKA----
XM_010569846.1:Haliaeetus leucocephalus                  NDSNDTDESDEVVTSFPTDIPVT-VPFPPFPF----TRGDNSGRGDSVAYRVRAKA----
XM_011600806.1:Aquila chrysaetos canadensis              NDSNDTDESDEVVTSFPTDIPVT-VPFPPFPF----TRGDNAGRGDSVAYRVRAKA----
XM_009490811.1:Pelecanus crispus                         NDSDDTDESDEVVTSFPTDIPVT-VPFPPFPF----TRGDNAGRGDSVAYRVRAKA----
XM_009694723.1:Cariama cristata                          NDSNDTDESDEVVTSFPTDIPVT-VPLPPFPF----TRGDNAGRGDSVAYRVRAKA----
XM_010311221.1:Balearica regulorum gibbericeps           NDSNDTDESDEVVTSFPTDIPIT-VPFPPFPF----TRGDNSGRGDSVAYRVRAKA----
XM_010287426.1:Phaethon lepturus                         NDSNDTDESDEVVTSFPTDIPVT-VPFPPFPF----TRGDNAGRGDSVAYRVRAKA----
XM_009890748.1:Charadrius vociferus                      NDSNDTDESDEVVTNFPTDIPVT-VPFPPFPF----TRGDNIGRGDSVAYRVRAKA----
XM_009584852.1:Fulmarus glacialis                        NDSNDTDESDEIVTSFPTDIPVT-VPFPP--F----TRGDNAGRGDSVAYRARAKA----
XM_009467655.1:Nipponia nippon                           NDSNDTDESDEVVTSFPTDIPVT-VPFPPFPF----TRGDNAGRGDSVAYRVRAKA----
```

M59182.1:*Gallus*	---HVVKASKLRKAARKLI-EDDATA-----EVGDSQLAGL-------------------
XM_003205565.3:*Meleagris gallopavo*	---HMVKASKLRKTARKLI-EDDATA-----EAGGSQLAGL-------------------
XM_021395699.1:*Numida meleagris*	---HVVKASKLRKVARKLI-EDDATE-----EVGDSQPVGL-------------------
NM_001323234.1:*Coturnix japonica*	---HVVKASKIRKAARKLI-EDDATT-----EDGDSQPAGL-------------------
XM_010218705.1:*Tinamus guttatus*	---AVMKPSKLHKAARKLMVHDVTEE-DLSALAADSHQEGFSREAFG-AHRSAEKYADSL
XM_009676586.1:*Struthio camelus australis*	---LVVKPSKLHKAARKLIVHDATEE-DESALAADSRQAGLSREDLG-AHRSAGKYADSG
XM_013957376.1:*Apteryx australis mantelli*	---TVVKSSKLHKAARKLIVHDATEE-DESALVTDSHQAGLSQEDLG-ARHLAGKYADSG
XM_021526515.1:*Lonchura striata domestica*	---ALLKYIKFHKDAKKLL-YDATEE-DESDMDADSQR-PLSRENPA-FRRSLTKPASSV
XM_002190366.1:*Taeniopygia guttata*	---ALLKYIKFHKDAKKLL-YDATEE-DESDMDAASQR-SLSRENPA-FRRSLTKPSSSV
NW_008240042.1:*Corvus brachyrhynchos*	---ALLKSIKLYKAAKKLI-YGATEE-DESDMDADSQR-SVSREDSA-SRSSLRKHASSV
XM_010412044.2:*Corvus cornix cornix*	---ALLKSIKLYKAAKKLI-YGATEE-DESDMDADSQR-SVSREDSA-SRSSLRKHASSV
XM_015623543.2:*Parus major*	---TLLKSIKLHKAAKKLI-YDATEE-DESYMDADSQR-SVSREDSA-SRSSLRKHASS-
XM_005525626.2:*Pseudopodoces humilis*	---TLLKSIKLHKAAKKLI-YDATEE-DESYMDADSQR-SVSREDSA-SRSSLRKHASS-
XM_014878941.1:*Sturnus vulgaris*	AKAALLKSVKLHKAAKKLI-YDATEE-DESDMDADSQR-SVSREDAA-PRRSLKKHASRV
XM_016297843.1:*Ficedula albicollis*	AKAKLLKSVKLHKAAKKLI-YDATEE-DESDLDADSQR-SVSREDSA-SRSSLRKHASSV
NW_007931124.1:*Serinus canaria*	---ALLKSIKLHKATKKLI-YDATQE-DESDMDADSQR-SVSREDSA-SRRSLRKHASSM
XM_005418195.1:*Geospiza fortis*	---ALLKSIKLHKAAKKLI-YDATEE-DESDTDADSQR-SVSREDSA-SRRSLRKHASSV
XM_005485296.1:*Zonotrichia albicollis*	---ALLKSIKLRKATKKLI-YDATEE-DESDMDAESQH-SVSREDPA-SRRSLRKHASSV
XM_009904975.1:*Picoides pubescens*	---RLLKSSKLGKAAQKHCKATRSNQTAESAEQARD-------------RHSIERNEVTL
XM_005012779.3:*Anas platyrhynchos*	---SMVKSSKLRKAARKLIADDATEE-DESALPGDSQQVGLWQQELA-AHRAPGKRAVSG
XM_013190510.1:*Anser cygnoides domesticus*	---SMVKSSKLRKAARKLIADDATEE-DESALHGDSQQVGLWQKELA-AHRAPGKRAVSG
KZ506572.1:*Limosa lapponica baueri*	---AVVKSSKLRKAAKKLIVYDASED-GESALDAGSQQAGLSQEGAA-ARRSLGKHVISG
XM_005498492.2:*Columba livia*	---TVVKSSKIRKAAKQVIAYDTKEE-DESALDADSRQGRLSQEDLS-ARRSLGKLSITG
LSYS01006159.1:*Patagioenas fasciata monilis*	---TVVKSSKIRKAAKQLIVYDDTEE-DESTLDADSRQGHLSREDLS-ARHSLGKLSITG
XM_008500834.1:*Calypte anna*	---TALQSSKLHKAAKKLIVYDATEE-DESALNAVSHQAEFSQEGPY----VLGKHTVSE
XM_009993754.1:*Chaetura pelagica*	---VALKSSKHLKAAKKLVVGEDSEE-DESTLEAASQQAELSQEARA-AQGFPRKHGIRG
XM_008942236.1:*Merops nubicus*	---TVVRSGKLRKAAKQLLGYDTAEE-DESALDAASQQAGLPWGAPA-ARRSLGKHDISG
XM_010019624.1:*Nestor notabilis*	---TVVKSSKPRKAAKKLIAYDATEE-DESALDADSQQVGLSQEHPA-AHRSLVK-HISR
XM_005146580.1:*Melopsittacus undulatus*	---SVVKSSNLRKAAKKLIAYDATEE-DESALDADSQQVGLSQEHPA-ARRSLVK-HISR
XM_009559526.1:*Cuculus canorus*	---IVMKASKLHKPAKKLTVHDATEE-NNSALDADSQQ-GLSQEDSA-SDRSLGTHTIIG
XM_005444503.1:*Falco cherrug*	-----VKSSKLRKAAKKLIAYDATEE-DESVPDADSQQAGLSQEDSA-TRHSLGKHTVSG
XM_005233595.1:*Falco peregrinus*	-----VKSSKLHKAAKKLIAYDATEE-DESVPDADSQQAGLSQEDSA-TRHSLGKHTVSG
XM_009073853.1:*Acanthisitta chloris*	---ALVKAIKLHKAAKKLM-YDATEE-DESDLDADSQQTGLSQEDSA-SHSSLGKHASSM
XM_008930247.2:*Manacus vitellinus*	---ALVKSIKLRKDAKKVM-YDATVE-DESDLDADSQRAGFSREDSA-SRGSLEKHAISV
NW_016690259.1:*Lepidothrix coronata*	---ALVKSIKLRKDAKKVM-YDATVE-DESDLDADSQRAGFSREDSA-SRRSLEKHAISV
XM_010202192.1:*Colius striatus*	---TVVKSSKLRKAAKKLIAYDATEE-DVSALDADSQQAGSQ-EGP-AALRSLGKHGISG
XM_010173470.1:*Caprimulgus carolinensis*	---AAMKARKLRKAAKKLIVYDATEE-DESTLDADSQQAGLSQEDPA-VRRALGK-YISR
XM_010181850.1:*Mesitornis unicolor*	---TLVKSSKLRKAAKKLMVYDATKE-DESDLDAASQQAGLSWEDSA-AHRSLGKHISSG
XM_009868641.1:*Apaloderma vittatum*	---TAVKSSKLHKAPKKLI-YDATEE-DESALDADSRARSLE---VPAARVSLGKHSISG
XM_009958978.1:*Leptosomus discolor*	---TMV-SSKLRKAAKKLIAHDATEE-DEGALDADSQQVGLAQEDLA-APRSLGKHAISR
XM_010079868.1:*Pterocles gutturalis*	---AVVKASNLRKAAKKLIVYDATEE-DESTLDVDSQQAI-SR-EAAVARRSLGKHTLSG
XM_010119303.1:*Chlamydotis macqueenii*	---ALVKAIKLRKAAKKLIAYDATEE-DESALDADSQQAGLSQEDPA-VRRTLGKHTISR
XM_009970430.1:*Tyto alba*	---TVVKSSKLRKAAKQLIEYDATTE-DESALDADSQQAGFSQEHLA-ARRSLGKHAISG
XM_009932280.1:*Opisthocomus hoazin*	---TAVKSSRLRKAAKKLIVYDATEERDESALDADSQQAGLSWEDPV-ARLSLGKHAISG
XM_010145609.1:*Buceros rhinoceros silvestris*	---AVLKSSKLHKAAKKLIAYDATGE-D----DADSQQAGLSREDAA-VHHSLGKHTISG
XM_009978083.1:*Tauraco erythrolophus*	---VAVKSSKLRKAAKKLIVYDATEE-DESTLDTDSQQAGLSQEDPA-ALHSLRKHAVSG
XM_010154680.1:*Eurypyga helias*	---TAVKASKLHKVAKKLILYDATNE-DEGDLDADSQQAGLSQEDSAAAHLSLAKHAISR
XM_009639993.1:*Egretta garzetta*	---AVVKPSKLHKAAKKLIVYDATEE-DESALDADSQQAGLSQEDAA-AYRSLGKQAISR
XM_009327481.1:*Pygoscelis adeliae*	---TVVKSSKLRKAAKKLIVYDATEE-DESALDVDSQQAGLSREDPA-ACRSLGKHAISG
XM_009280227.1:*Aptenodytes forsteri*	---TVVKPSKLRKAAKKLIVYDATEE-DESALDVDSQQAGLSREDPA-ACRSLGKHAISR
XM_009512482.1:*Phalacrocorax carbo*	---TVVKSSKPHKAAKKLIVYDATEE-DESALDVDNQQAGLSQEEPA-AHRSLGKHPISG
XM_014961493.1:*Calidris pugnax*	---AVVKSRKLRKAAKKLIVYDASED-DESALDAGSQQAGLSWEDAA-ARRSLGKHAISG
XM_009813620.1:*Gavial stellata*	---SVVKSNKLHKAAKELIEYDATEE-DESALDADSQQAGLSREDSA-VRRSLGKHAISG
XM_009916485.1:*Haliaeetus albicilla*	---TVVESSKLRKAAKKLIVYDASEE-DESALDAHSQQTGLSRE----------KHAISR
XM_010569846.1:*Haliaeetus leucocephalus*	---TVVESSKLRKAAKKLIVYDASEE-DESALDAHSQQTGLSRE----------KHAISR
XM_011600806.1:*Aquila chrysaetos canadensis*	---TVVESRKLRKAAKKLIVYDASEE-DESALDADSQQTGLSREDPA-AHHSLGKHAISR
XM_009490811.1:*Pelecanus crispus*	---TVVKSSKLRKAAKKLTVYDATEE-DESALDADSQQAGLSQEDPA-VHRSLEKHAISG
XM_009694723.1:*Cariama cristata*	---TIVKSSKLSKAAKKLILYDASEE-DESALDADSQQAGLSQEDSA-ARRSLGKHAISR
XM_010311221.1:*Balearica regulorum gibbericeps*	---AMVKSSKLSKAAKKLIVYDTTEE-DESTLDADSQQAGLSREDPA-ARRSLGKHAISR
XM_010287426.1:*Phaethon lepturus*	---TVVKPSKLHKAAKKLIVYDPTEE-DESALDADSQQAELSREDPA-AHRSLGKHTISE
XM_009890748.1:*Charadrius vociferus*	---AVVKPSKLRKAAKKLIVYDATED-DDSALDADSQQAGLSWDDPA-ARRSLGKHAISG
XM_009584852.1:*Fulmarus glacialis*	---TVVKSSKLNKAAKKLAVYDATEE-DKSALDADSQQAGLSREDPA-AHHSLGKHAISG
XM_009467655.1:*Nipponia nippon*	---TMVKSSQLRKAAKKLVVYDATEE-DESALDADSQQAGLSREDPA-AHRSLGKHAISR

```
M59182.1:Gallus                                    WLPKESREQ---DSRELAQHQSVENDSRPRF---DSPEVGGGD-SKASAG----------
XM_003205565.3:Meleagris gallopavo                 WWPKESREQ---DSRELTQHQSVENDSRPRF---DSPEVDGGD-SKASAG----------
XM_021395699.1:Numida meleagris                    WWPKESREQ---ESRELAQHQSVENDSRPKF---DSREVDGGD-SKASAG----------
NM_001323234.1:Coturnix japonica                   WWPKESREQ---NSRELPQHQSVENDSRPKF---DSREVDGGD-SKASAG----------

XM_010218705.1:Tinamus guttatus                    EWGDKSHGHDSNEVGRKLPERSLEKDSRQKF---DSPERD--S-GEWGARGDSPPS----
XM_009676586.1:Struthio camelus australis          EWRDKSRGQDSDEVVRKVPDRSLENDSRQKL---DSLEAEVGD-SR-------------
XM_013957376.1:Apteryx australis mantelli          EWGHKSRGRDSNEVVRKLPDWSVENDSRQKF---DSPEVERDS-SERGARGGIPLS----

XM_021526515.1:Lonchura striata domestica          VWSDRSHGRDGSEQDSEPHHRSLDNDSWPKS---DSREAQSDS-SKPGVRRDSHPSVESR
XM_002190366.1:Taeniopygia guttata                 VWSDQSHGRDSSEQDSDPHHRSLENLSWPKS---DSHEAQGDS-SKWFHP-SSMPFLPAQ
NW_008240042.1:Corvus brachyrhynchos                VWSDQSHGRDSSEQDSNPHDRSLENNSRHKS---DSHEAEGDS-SKSGVRGDSLSTVESK
XM_010412044.2:Corvus cornix cornix                VWSDQSHGRDSSEQDSNPHDRSLENNSRHKS---DSHEAEGDS-SKSGVRGDSLSSVESK
XM_015623543.2:Parus major                         VWADQSHGRDSSEQDSDPRHRSLENDSWHES---ASHEAEGDS-SKSGVRADSLSSTETK
XM_005525626.2:Pseudopodoces humilis               VWSEQSHGRDSSEQDSDPRHRSLENDSWHKS---ASHEAEGDS-SKSGVRGDSLSSMETK
XM_014878941.1:Sturnus vulgaris                    AWSDRSHGRDSGEQDSDLRAWSLENDSRHKS---DSHEAEGDS-SKSAT----------
XM_016297843.1:Ficedula albicollis                 AWSDQSHGRDSSEQDSDLRARSLENDSRHKS---DSHEAEGDS-SKSGVRGDSLSSVESR
NW_007931124.1:Serinus canaria                     VWSDQSHGRDSSEQDSDPHYRSLENDSRPDS---HE--AEGDS-SESGIRGDSHSSVESR
XM_005418195.1:Geospiza fortis                     VWSDQSHGRDSSEQDSDPHDRSLENDSRPKS---DSHEAESDS-SKPGM----------
XM_005485296.1:Zonotrichia albicollis              VWSDRSHGRDSSEQDSHPHDRSLENDSRPKS---DSHEAEGDS-SKSGM----------

XM_009904975.1:Picoides pubescens                  ------------------------------------------------------------
XM_005012779.3:Anas platyrhynchos                  EWLDKSHERDSKELTS---HQSMENDSRQKF---DSPEVEGGD-SRDGVRD-DHHS----
XM_013190510.1:Anser cygnoides domesticus          EWLDKSHERDSSELTH---HQSMENDSRQKF---DSPEVEGGD-SQDGARGDGRHS----
KZ506572.1:Limosa lapponica baueri                 EWADKSHGQDSSELDSKQHDQSTENDSRQKF---DSHEAEGDD-SKVSIRGDSHQS----
XM_005498492.2:Columba livia                       EWAAKGHRQDSSELDSKLRDRSVENDSRQKF---DSREMEGDDDSKAGVRGDSHWS----
LSYS01006159.1:Patagioenas fasciata monilis        KWAAKGHEQDSSELDSKLRDRSVENDSRQKF---DSREMERDDDSKAGVRGDSHWS----
XM_008500834.1:Calypte anna                        EWAGKSDRQDSSQQDSKQHDQSMENNSLQKS---DSHETKGDD-SKADVRADSHQS----
XM_009993754.1:Chaetura pelagica                   EWADRSHQQDSSELDGKQRDWSAENDSRQKF---NSHEVERDG-SKFGARWDSHQS----
XM_008942236.1:Merops nubicus                      EWADKGQGHDSLELGSKQHHHSMENDSRQKF---DSHEAED--DSQAGV------F----
XM_010019624.1:Nestor notabilis                    EWADKSQGQDSRELDSKQPDWXXXXXXXXXXXGKKSASHEAEDDSKAGIREDSHHS----
XM_005146580.1:Melopsittacus undulatus             EWADKSHGQDSSELDSKQRDRSLEN---DSRQKFDSHEA--DDDSKSGIREDSHHS----
XM_009559526.1:Cuculus canorus                     EWADKSREQDSSELDSKLHDRSVENDSRQKF---DSHKTEGDDNSKAGIRADSHHS----
XM_005444503.1:Falco cherrug                       EWADKSHGQDSSELDSKHCDQSVENDSWQKF---DSREAEA--DSKASVRGDSHQS----
XM_005233595.1:Falco peregrinus                    EWADKSHGQDSSELDSKHCDQSVENDSWQKF---DSREAEA--DSKASVRGDSHQS----
XM_009073853.1:Acanthisitta chloris                EWSDKSHGQDSSDLDSNQRDQSLENDSRQKF---ESHEADSAD-SKSGVRGDSHPS----
XM_008930247.2:Manacus vitellinus                  EWSDKSHGQDSSELDSNQPDRSLENDSRQKF---DRHEAEGDD-SKSAVRGDSFPS----
NW_016690259.1:Lepidothrix coronata                EWSDKSHGQDSSELDSNQPDRSMENDSRQKF---DSHEAEGDD-SKSAVRGDSFPS----
XM_010202192.1:Colius striatus                     EWADKSHQQDSNKQDSKQRDQSMENDSQQKF---DSHEVEGNY------SKDSQQS----
XM_010173470.1:Caprimulgus carolinensis            EWSDKNHGQDSSELDSKQRDQSMENDSRQKF---DSHEVEADV-SKASVKEDSHQS----
XM_010181850.1:Mesitornis unicolor                 KWADKSHGQDSSELDSKLRDWSMEN-SQQKF---DSREREGDDDSKASVRGDSHQS----
XM_009868641.1:Apaloderma vittatum                 EWADKSRGQDSSELDSKQRDRSMENDSRQKF---DSHEVERDD-RKAGVRGDSHQS----
XM_009958978.1:Leptosomus discolor                 EWADKSHRQDSSELDSKQRDQSLENDSRQKF---DSHEA--GDDSKAGVRGDSHQS----
XM_010079868.1:Pterocles gutturalis                EWADKSHGQDSSELDSKLRDRSVENDSRQKL---DSREVEGDDDSKAGVRVDSRQS----
XM_010119303.1:Chlamydotis macqueenii              EWADKSHEQDSSELDSKLRDRSMENDSRQKF---DSREMEGDDESKASIRGDSHQS----
XM_009970430.1:Tyto alba                           EWADKSHGQDSSELDSKQRDQSMENDSRQKF---DSHEA-GDD-SKAGARGDSRQS----
XM_009932280.1:Opisthocomus hoazin                 EWADKSHRQDSSELDSKQHDQSIENDSRQKF---DSHEVEGDD-SKAGARVDSHQS----
XM_010145609.1:Buceros rhinoceros silvestris       EWADKSHGQDSSELDSKQQDRSMENDSRQKF---DSREVEGDD-SKAGVRGNSHQS----
XM_009978083.1:Tauraco erythrolophus               EWADKSHRQDSSELDSKQRDRSTENDSRQKF---DSHEAEGDD-SKAVVRRERHQS----
XM_010154680.1:Eurypyga helias                     EWADKSHRQDSSELDSKQRDRSMENDSRQKF---DSHEAE-DD-SKAGVRGDSHQS----
XM_009639993.1:Egretta garzetta                    EWADKSHGQDSSELDSKQRDRSMENDSRQKL---DSHEAE-DD-SKAGVRGDSHQS----
XM_009327481.1:Pygoscelis adeliae                  EWADKSHGQDSSELDSKQRDRSVESGSRQKL---DSHEAE-DD-SKAGVRGNSHQS----
XM_009280227.1:Aptenodytes forsteri                EWADKSHGQDSSELDSKQRDRSMENGSRQKL---DSHEAE-DD-SKAGVRGDSHQS----
XM_009512482.1:Phalacrocorax carbo                 EWADKSRRQDSNELDSKHRDQSMENDSRQKF---DSHEAEGDD-SKAGVREGSHQS----
XM_014961493.1:Calidris pugnax                     EWADKSHGQDTSELDSKQRDQSMENDSRQKF---DSHEVEGDD-SKASIRVDSHQS----
XM_009813620.1:Gavial stellata                     EWADKSHGQDSSELDSKQRDRSMENDSRQKF---DSREVEGDD-SKAGVRGDSHQR----
XM_009916485.1:Haliaeetus albicilla                EWADKSHGQDSSELDSKQHDRSMENDSRQKF---DSHEA-GDD-SKAGVRGDSYQT----
XM_010569846.1:Haliaeetus leucocephalus            EWADKSHGQDSSELDSKQHDRSMENDSRQKF---DSHEA-GDD-SKAGVRGDSYQT----
XM_011600806.1:Aquila chrysaetos canadensis        EWADKSHGQDSSELDSKQHDRSMENDSRQKF---DSYEAEGDD-SKAGVRGDSYQT----
XM_009490811.1:Pelecanus crispus                   EWADKSHGQDSSELDSKQRDWSVENDSQQKF---NSHEVEGDD-SKAGVRGDSHQS----
XM_009694723.1:Cariama cristata                    EWADKSHGRDSSEPDSKQRDQSMENDSRQKF---DSHEVEVDD-SKAGVRGDSHQS----
XM_010311221.1:Balearica regulorum gibbericeps     EWADKSRGQDSSELDSKQRDRSMEKNSQQKF---DSHEVERDD-SKAGVRGDSHQS----
XM_010287426.1:Phaethon lepturus                   EWADKSHREDSSEPDSKQHDRSMENDSQQKF---DSHEAERDD-SKAGVRGDSHLS----
XM_009890748.1:Charadrius vociferus                EWADKSHGQDSSELDSKQHDQSMENDSRQKF---DSHEAERDD-SKAGVRGDSHQS----
XM_009584852.1:Fulmarus glacialis                  EWADKSHGQDSSELDSKQHDRSMENDSRQKF---DSHEAERDD-SKAGVRGDSHQS----
XM_009467655.1:Nipponia nippon                     EWADKSHGQDSSELDSKQHDRSMENDSRQKF---DSHEVERDD-SKASVRGDSHQS----
```

M59182.1:*Gallus*	--
XM_003205565.3:*Meleagris gallopavo*	--
XM_021395699.1:*Numida meleagris*	--
NM_001323234.1:*Coturnix japonica*	--
XM_010218705.1:*Tinamus guttatus*	--
XM_009676586.1:*Struthio camelus australis*	--
XM_013957376.1:*Apteryx australis mantelli*	--
XM_021526515.1:*Lonchura striata domestica*	ERGDSHPSVE------------------------SRERGDSHPSVESRERGDSHPSVESR
XM_002190366.1:*Taeniopygia guttata*	KKNM--
NW_008240042.1:*Corvus brachyrhynchos*	E--RGDSHPSAESW
XM_010412044.2:*Corvus cornix cornix*	E--RGDSHPSAESW
XM_015623543.2:*Parus major*	E--REDSHPS----
XM_005525626.2:*Pseudopodoces humilis*	E--RGDSHPS----
XM_014878941.1:*Sturnus vulgaris*	--
XM_016297843.1:*Ficedula albicollis*	E--RGDSHPS----
NW_007931124.1:*Serinus canaria*	EKGDSLSSVESRERGDSLSSVESREKGDSLSSVESREKGDSLSSVESREKGDSLSSVESR
XM_005418195.1:*Geospiza fortis*	---RGDNLSSVESR
XM_005485296.1:*Zonotrichia albicollis*	---RGDSLSSVESR
XM_009904975.1:*Picoides pubescens*	--
XM_005012779.3:*Anas platyrhynchos*	--
XM_013190510.1:*Anser cygnoides domesticus*	--
KZ506572.1:*Limosa lapponica baueri*	--
XM_005498492.2:*Columba livia*	--
LSYS01006159.1:*Patagioenas fasciata monilis*	--
XM_008500834.1:*Calypte anna*	--
XM_009993754.1:*Chaetura pelagica*	--
XM_008942236.1:*Merops nubicus*	--
XM_010019624.1:*Nestor notabilis*	--
XM_005146580.1:*Melopsittacus undulatus*	--
XM_009559526.1:*Cuculus canorus*	--
XM_005444503.1:*Falco cherrug*	--
XM_005233595.1:*Falco peregrinus*	--
XM_009073853.1:*Acanthisitta chloris*	--
XM_008930247.2:*Manacus vitellinus*	--
NW_016690259.1:*Lepidothrix coronata*	--
XM_010202192.1:*Colius striatus*	--
XM_010173470.1:*Caprimulgus carolinensis*	--
XM_010181850.1:*Mesitornis unicolor*	--
XM_009868641.1:*Apaloderma vittatum*	--
XM_009958978.1:*Leptosomus discolor*	--
XM_010079868.1:*Pterocles gutturalis*	--
XM_010119303.1:*Chlamydotis macqueenii*	--
XM_009970430.1:*Tyto alba*	--
XM_009932280.1:*Opisthocomus hoazin*	--
XM_010145609.1:*Buceros rhinoceros silvestris*	--
XM_009978083.1:*Tauraco erythrolophus*	--
XM_010154680.1:*Eurypyga helias*	--
XM_009639993.1:*Egretta garzetta*	--
XM_009327481.1:*Pygoscelis adeliae*	--
XM_009280227.1:*Aptenodytes forsteri*	--
XM_009512482.1:*Phalacrocorax carbo*	--
XM_014961493.1:*Calidris pugnax*	--
XM_009813620.1:*Gavial stellata*	--
XM_009916485.1:*Haliaeetus albicilla*	--
XM_010569846.1:*Haliaeetus leucocephalus*	--
XM_011600806.1:*Aquila chrysaetos canadensis*	--
XM_009490811.1:*Pelecanus crispus*	--
XM_009694723.1:*Cariama cristata*	--
XM_010311221.1:*Balearica regulorum gibbericeps*	--
XM_010287426.1:*Phaethon lepturus*	--
XM_009890748.1:*Charadrius vociferus*	--
XM_009584852.1:*Fulmarus glacialis*	--
XM_009467655.1:*Nipponia nippon*	--

```
M59182.1:Gallus                                     --------VDSRESL--ASR---SAVDTSNQTLESAEDAEDRHSIENNEVTR
XM_003205565.3:Meleagris gallopavo                  --------VDSRESQ--GSV---PAVDTSNQTLESAEDAEDRHSIENNEVTR
XM_021395699.1:Numida meleagris                     --------VDSRESQ--GSV---PAVDTSNQTLESAEDAEDRHSIENNEVTR
NM_001323234.1:Coturnix japonica                    --------VDSRESQ--GSV---PAVDASNQTLESAEDAEDRHSIENNEVTR

XM_010218705.1:Tinamus guttatus                     --------RESRESQARVSAE-VPDGDNSNQTLESAEDAQDHLSIKHNEVIF
XM_009676586.1:Struthio camelus australis           ----------------------------------------------------
XM_013957376.1:Apteryx australis mantelli           --------RESRESPAHVLTRVIVVDDNSNQTFESAEDVQDLRSIKNNEVAL

XM_021526515.1:Lonchura striata domestica           ERGDSHPSVESRESP--DRVSAELADDISSQTLESAEDSQDRHSIESNEVTL
XM_002190366.1:Taeniopygia guttata                  ----------------------------------------------------
NW_008240042.1:Corvus brachyrhynchos                 ESRDSHPSAESRESQ--DRVSAELSDDISNQTLESAEDSQDRRSIESNEVTL
XM_010412044.2:Corvus cornix cornix                 ESRDSHPSAESRESQ--DRVSAELSDDISNQTLESAEDSQDRRSIESNEVTL
XM_015623543.2:Parus major                          --------VESRESR--DRVSAELSDGISNQTLESAEDSQDRHSIESNEVTL
XM_005525626.2:Pseudopodoces humilis                --------VESRESR--DRVSAELSDGISNQTLESAEDSQDRHSIESNEVTL
XM_014878941.1:Sturnus vulgaris                     ----------GVESR--DRVSEELSDDISNQTLESAEDSQDRHSIESNEVTL
XM_016297843.1:Ficedula albicollis                  --------VESRESR--DRVSAELSDDISNQTLESAEDSQDRHSIESNEVTL
NW_007931124.1:Serinus canaria                      ERGDSHPSVESRESR--DRVSAELSDDIITQTLENAEDSQDRHSIESNEVTL
XM_005418195.1:Geospiza fortis                      ERGDSHPSVESRESR--DRVSAELSDHISSQTLESAEDSQDRHSIESNEVTL
XM_005485296.1:Zonotrichia albicollis               E---SHLSVESRERR--DSHSTELSDHISIQKLESAEDSQDRHSIESNEVTL

XM_009904975.1:Picoides pubescens                   ----------------------------------------------------
XM_005012779.3:Anas platyrhynchos                   --------MESRERRVSAAAGIPAIDDTSNQTLESAENTQERHSIENNEVTI
XM_013190510.1:Anser cygnoides domesticus           --------LESRERRVSAAASIPTIDDTSNQTLESAENAREHHSIENNEVTI
KZ506572.1:Limosa lapponica baueri                  --------MESRESQ--VRVSAENTNDNSNQTLESAEDAQDHHSIENNEVTL
XM_005498492.2:Columba livia                        --------AESRESQ--ARILPEIPDEDSNQTLESAEDAQDRHSIENNEVTL
LSYS01006159.1:Patagioenas fasciata monilis         --------VESRESQ--ARVLPEIPDDNSNQTLESAEDAQDRHSIENNEVTL
XM_008500834.1:Calypte anna                         --------MESKESQ--VQVPAEVPDDNSNQTLESAEDAQDHHSIENNEVTI
XM_009993754.1:Chaetura pelagica                    --------RESRESF-----PAGIPEDNSTQTLESAEDAGDHHSIDTNEVTL
XM_008942236.1:Merops nubicus                       --------LESRESQ--VRVSAEIPDDKSNQTLESAEDARDRDSIKNNEVTL
XM_010019624.1:Nestor notabilis                     --------VESRESQ--VRVSAEIPDDNSNQTLESIEDARDRHSIENNEVTL
XM_005146580.1:Melopsittacus undulatus              --------VESRESQ--VRVSAEIPDDNSNQTLESSEDAQDRHSIENNEVTL
XM_009559526.1:Cuculus canorus                      --------MESRESQ--VHVSAEIPDDNSNQTVESAEDARGQHSIENNEVTL
XM_005444503.1:Falco cherrug                        --------VESRERQQSSRVSAESPDDNSNQTLESAEDAHDRHSIENNEVTL
XM_005233595.1:Falco peregrinus                     --------VESRERQQSSRVSAESPDDNSNQTLESAEDAHDRHSIENNEVTL
XM_009073853.1:Acanthisitta chloris                 --------VESRESQ--DQASSEISDDTSNQTLESAEDSQDRHSLENNEVTL
XM_008930247.2:Manacus vitellinus                   --------VESRESQ--VRVSAEVSDDNSNQKLESAEDSQDRHSIENNEVTL
NW_016690259.1:Lepidothrix coronata                 --------VESRESQ--VRVSAEVSDDNSNQKLESAEDSQDRHSIENNEVTL
XM_010202192.1:Colius striatus                      --------MESRESQ--VLVSAEIPDDNSNQTVESTEDAQDRPSIENNEVTL
XM_010173470.1:Caprimulgus carolinensis             --------MESRESQ--VHVSAEIPDDNSNQTLESAEDARDRHSIENNEVTR
XM_010181850.1:Mesitornis unicolor                  --------IESKESQ--VHVSAEIPDDNSSQMLESAEDAQEHHSIKNNEVTL
XM_009868641.1:Apaloderma vittatum                  --------MESRESQ--VRISAEVPDDNSNETLESAEDARDHRSIENNEVTL
XM_009958978.1:Leptosomus discolor                  --------IESRESQ--VHISAEFPDDNSNQTVESAEDARDHHSIENNEVTL
XM_010079868.1:Pterocles gutturalis                 --------VESRESQ--VHVSAEIPDDNSNQTLESAEDARDRHAIENNEVTL
XM_010119303.1:Chlamydotis macqueenii               --------LESKESQ--VHVSSEIPDDNSNQTLESAEDAQDHHSIENNEVTF
XM_009970430.1:Tyto alba                            --------MESRESQ--AHVSAESRDDNSNQTLESTEDAQDHHSIENNEVTL
XM_009932280.1:Opisthocomus hoazin                  --------VESRESQ--VRISAEVPDDNSNQTAESAEDAQDRHSIENNEVTL
XM_010145609.1:Buceros rhinoceros silvestris        --------MESRESQ--VRVSAEIPDDNSNQTVESAEDAQDHHSIENNEVTL
XM_009978083.1:Tauraco erythrolophus                --------VESRESQ--VRISAEIPDENSNQTLESAEDARDRHSIENNEVTL
XM_010154680.1:Eurypyga helias                      --------TESKESQ--VRVSAEIPDDNSNQTLESAEDAQDRHSIENNEVTH
XM_009639993.1:Egretta garzetta                     --------MESKESQ--VHVSAEIPDDNSNQTLESTEDAQDHHSIENNEVTL
XM_009327481.1:Pygoscelis adeliae                   --------MESRESQ--VHVSAEIPDDNSNQTMESAEDAQDHHSIENNEVTL
XM_009280227.1:Aptenodytes forsteri                 --------MESKESQ--VHVSAEIPDDNSNQTTESAEDARDHHSIENNEVTL
XM_009512482.1:Phalacrocorax carbo                  --------MESRESQ--VHVSAEIPDDNSNQTLESAEDAQDHHSIENNEVTL
XM_014961493.1:Calidris pugnax                      --------MESRESQ--VRASAENPKDNSNQTLESAEDAQDHHSIENNEVTL
XM_009813620.1:Gavial stellata                      --------VESRESQ--VRVSAEIPDDHSNQTLESAEDAQDRHSIENNEVTL
XM_009916485.1:Haliaeetus albicilla                 --------MESRESQ--VRVSAETPDDNSNQTLESAEDARDHHSIENNEVTL
XM_010569846.1:Haliaeetus leucocephalus             --------MESRESQ--VRVSAETPDDNSNQTLESAEDARDHHSIENNEVTL
XM_011600806.1:Aquila chrysaetos canadensis         --------MESRESQ--VRVSAEIPDDNSNQTLESAEDARDHHSIENNEVTL
XM_009490811.1:Pelecanus crispus                    --------MESRESQ--VRVSAEIPDDNSNQTLESAEDARDHHSIENNEVTL
XM_009694723.1:Cariama cristata                     --------MESRESQ--VHVSAEIPDDNSNQTLESAEDSRDHHSIENNEVTL
XM_010311221.1:Balearica regulorum gibbericeps      --------VESRENQ--DRVSAEIPNDNSNQTLESAEDAQDHHSIENNEVTL
XM_010287426.1:Phaethon lepturus                    --------MESRESQ--VRVSAEIPDDNSNQTLESAEDAQDHHSIENNEVTL
XM_009890748.1:Charadrius vociferus                 --------MESRESQ--VRVSAEIPDDNSNQTLESAEDAQDHHSIENNEVTL
XM_009584852.1:Fulmarus glacialis                   --------MESRESQ--VHVSAEIPDDNSNQTLESAEDAQDHHSIENNEVTL
XM_009467655.1:Nipponia nippon                      --------MESRESQ--VHVSAEIPDDNSNQTLESAEDAQDHHSIENNEVTL
```

Table S1.14: Osteopontin in Aves. The large number of available sequences reveals 4 distinct subgroups (separated by free lines). The *Limosa lapponica baueri* sequence has a long unique N-terminal stretch, which requires corroboration.

```
NC_030431.1:Ictalurus punctatus                  -MHFDWLNPEAAGFKSQAAVSIKTKQDLKQETWKVVVDPLSDSSKKVRFTAAEEQNTPRK
NC_007121.7:Danio rerio                          ------------------------------------------------------------
NM_001124267.1:Oncorhynchus mykiss               ------------------------------------------------------------
AF223388.1:Salvelinus fontinalis                 ------------------------------------------------------------
NW_018128285.1:Monopterus albus                  ------------------------------------------------------------
AY651247.1:Sparus aurata                         ------------------------------------------------------------
NW_019174336.1:Seriola dumerili                  ------------------------------------------------------------
JN854244.1:Oreochromis mossambicus(partial)      ------------------------------------------------------------
NW_016094914.1:Kryptolebias marmoratus           ------------------------------------------------------------
NW_015443488.1:Nothobranchius furzeri            ------------------------------------------------------------

NC_030431.1:Ictalurus punctatus                  MKAVIVFVLLFAAVYCHPVKRSASSSESADL----QPVDTQV---------------LKV
NC_007121.7:Danio rerio                          MKSIIVLTLLVATVFCVPVKRSASSSESSEELVIVQRPPPILRKAAANVFQVEPT--QTT
NM_001124267.1:Oncorhynchus mykiss               MKAAIVFVLLFATVLGRPAIRSPGNNPESLEELVKPAPVLGIAP----AELTEVAPVQNV
AF223388.1:salvelinus_fontinalis                 MKAAIVFVLLFATVLGRPAIRSPGNSPESSEELVKPAPVLGIAP----AELTEVAPVQNG
NW_018128285.1:monopterus_albus                  MKVAIVTVLLFATVLCGPVRKASYRSSESSEEVVRRPAVPAIRKQAAVVPQSRAAHVQNV
AY651247.1:sparus_aurata                         MKVAIVFVLLFATVLCRPARKVADSS-ESSEEVVRKPAPPALKKQAAVVPQAPAAPVQDI
NW_019174336.1:seriola_dumerili                  MKVAVVFILLFATVLCRPARKVSDSSSDSSEEVVRQPAAPAVQTEAD------AAPAQNV
JN854244.1:oreochromis_mossambicus(partial)      ---------------------------ESSEEVVRQPKPPALRKQAPVVLKARPPPVQNV
NW_016094914.1:kryptolebias_marmoratus           MKVAVVFVLLFATVLCRPARKVSVSSSDSSEEVVRRPAAPALRKQALVAPKSRPSPVKKV
NW_015443488.1:nothobranchius_furzeri            MKVAVVFVLLFAAVLCRPARKVSDSSSDSSEEVVRRPAPPAVRKQAVVAAKHRAKPVQNV
                                                                           .:            :

NC_030431.1:Ictalurus punctatus                  TAVA-----D--------------------------------------VSSEE-DDDDDD
NC_007121.7:Danio rerio                          P-----TESNESTD------------------------------------SAD------D
NM_001124267.1:Oncorhynchus mykiss               DTAT---TSDESSDK-DETEGADPTS-------------DPTSDSADSADS---------
AF223388.1:salvelinus_fontinalis                 DTAT---TSDESSDE-DETEGADPTS-------------DPTSDSTASTDSTDSADSADS
NW_018128285.1:monopterus_albus                  VAAAA-TDSDESKGSSDEDESVVTT----------DGTNTALASDTTSDKSKDSGDDDDD
AY651247.1:sparus_aurata                         VAAA-AAGSDESSESSDEDAQAAPEA--PVEVQSSDSSDTASTSDTASVNSKDSEDSEDS
NW_019174336.1:seriola_dumerili                  VAAAA--GSDESKESSDEDELAAA--------------DTTSSSDTASVNSDDSDDDD--
JN854244.1:oreochromis_mossambicus(partial)      VAAAA-AGSGESAETSEEEEHQQQVAAEALPEVKSVVTDTT-SDSP-SVNTQDS-----E
NW_016094914.1:kryptolebias_marmoratus           VAAP-AAQSDESTETSEEDQGVAPVK-DAVKFKAISADPNPPTESPS-VISKDNDDDDDD
NW_015443488.1:nothobranchius_furzeri            VAAAAAAGSDESSETSEENQGVAVEK-ATVEFKAAGVDTNAAPVSDKGTNSQDSDDDDDD
                                                         .                                         :

NC_030431.1:Ictalurus punctatus                  ----------------DSDSDESDENGEP-------TSSPPIIETT--------------
NC_007121.7:Danio rerio                          TEEADEESETDEKEEENETDSSESESGESDTTIIPVTVDP------------------TL
NM_001124267.1:Oncorhynchus mykiss               --------ADSADSADSDDTDESSESEEADTTAAPATAE-------PTVEPTMATTEAP-
AF223388.1:salvelinus_fontinalis                 -----NESKDSADSADSDDTNESSESEEADTTAAPPTAE-------PTVEPTMATTEAP-
NW_018128285.1:monopterus_albus                  --DNDDETK------ETEEDESSSESGES-STLAPATDTPVIITDEPVAETTVEPIVPTI
AY651247.1:sparus_aurata                         --DDDDETEE---SETEEEDSESSESGES-STAVPSTLTPVIVTDAPVAETTPQPIDPTI
NW_019174336.1:seriola_dumerili                  -----DEAEE--ADTDEDDSSDSSESGES-STPAPTTASPVIITEEPVIETTVSPIEPTI
JN854244.1:oreochromis_mossambicus(partial)      DGDDDDEAEESETEEEEDESSDSSESGES-STPAPSTVAPVVVTETPAPEPTDDSIVATV
NW_016094914.1:kryptolebias_marmoratus           DADDDEGTEETDEDEDESNDSSDSESGES-STPAPGTLSPAIVTAEPVPETTVDTILPSI
NW_015443488.1:nothobranchius_furzeri            ------EAEESEAEEEEDESDSDSESGES-STPAPGTVSPVVVTVEPLPGTTVDTILPTI
                                                                  .  ....*. *        :
```

```
NC_030431.1:Ictalurus punctatus                LIPVINGRGDSI-GFQSDYKKPVYYVDGNNIEKGPSSHKSYGSD-----------KTKYD
NC_007121.7:Danio rerio                        GPIINTGRGDSL-GYPSDYKKSIVYVDAKDFEKLPSPYKSYSSDKLGGLTFVSKKTSAYD
NM_001124267.1:Oncorhynchus mykiss             --IYDDGRGDSM-GYPSDYKKSIIYVDTNNIEKGPSPYKSYGKI--DEGMYAGKKVSVYN
AF223388.1:salvelinus_fontinalis               --IYDDGRGDSM-GYPSDYKKSIIYVDTNNIEKGPSSYKAYGKM--DEGLYTGKKVSLYD
NW_018128285.1:monopterus_albus                VTDADTGHGDNMRGYPGDY-KSIVYVEDKSYHKIPVAYKSYEFV--GTGK-----KTAYD
AY651247.1:sparus_aurata                       VTDTETGRGDSYGGYPSDY-KSIDYVEEKTYYKVPVPYKSYELV--GTGK-----KMAYD
NW_019174336.1:seriola_dumerili                VTDTETARGDNYGGYPGDY-KSIVYVEDKSYHKVPAPYKSYEFV--GAGK-----KMAYD
JN854244.1:oreochromis_mossambicus(partial)    VTD--TARGDNLGGYPSEY-KSIVYVEEKSYHKAPGPYKSYEFV--DTGK-----KTAYS
NW_016094914.1:kryptolebias_marmoratus         VTDTDSGRGDSLGGYPGDY-KSIMYVEEKSYHKVPVPYKSYEIV--GAEK-----KLGYD
NW_015443488.1:nothobranchius_furzeri          VTDPDAGRGDNLGGYPGDY-KSI-YVEEKTYHKMSGPYKSYEFV--DSGK-----KTGYD
                                                     .:**.  *: .:* * : **: :   *    :*:*                 *.

NC_030431.1:Ictalurus punctatus                GA-------DLKVYKAL--QVHNV----------GLDQGVDASSGTDSDDASQQEQKAAS
NC_007121.7:Danio rerio                        DQSINDVEKEITLYKAL--QVHDLE-DGNTSTSEIDNIEAN------------ERQAALG
NM_001124267.1:Oncorhynchus mykiss             TGLGNKIEKTMTVFKAL--QVHDLM-EEDTSTPDVESQVLDASSGKAEESSMRKAHVDVA
AF223388.1:salvelinus_fontinalis               TGLGNEIEKKMTGFKAL--QVHDLM-EEDTSTPDVESQVLDSSSGKAEEPQQRQAHVDVA
NW_018128285.1:monopterus_albus                MTEGNEVEKSLKVYKAI--QVHSDLVEEDTSTPEVESQGLDASSVTTQD--HQEEGASP-
AY651247.1:sparus_aurata                       MTDGNEVEKSLQVYKAL--QVHSDILEEDTSTPEVESQGLDTSSGISQD--QDLRQASLP
NW_019174336.1:seriola_dumerili                MTDGNEVEKSLKVYKAL--QVHSDILEEDTSTPEVESQGLDATSQ-DQA--ISPRQASLP
JN854244.1:oreochromis_mossambicus(partial)    MTGGNEVEKLPKVYKTIY--VNPELLEEDTST----------------------------
NW_016094914.1:kryptolebias_marmoratus         MTDVNEVEKLPKVYKVQAFQIQSDILEEDTSTPEVESQGLDVSSGTSQDQDINPRQASLP
NW_015443488.1:nothobranchius_furzeri          MTDGNEVEKSPKVYKVQAFHTQSQLLEEDTSTPEVETQSLDVSSGTSQDQDISHRQASLP
                                                            :*.       ::

NC_030431.1:Ictalurus punctatus                ---------------------SSSAS---------------DAVPSQ-ESEEDSQSSEEN
NC_007121.7:Danio rerio                        AQEIVPVGDQAA--------------TEEGASARDSPSDSASASASQ-EENEEESDSEET
NM_001124267.1:Oncorhynchus mykiss             SQDRTPSESPESQKAKSLESDATSEL----TSSASSDGTSESTSTSASNETDSSNSSEEA
AF223388.1:salvelinus_fontinalis               SQDRTPSESPESQKAKSLESDATSVR----TSSASSDGTSESTSTSASNETDSSNSSEEA
NW_018128285.1:monopterus_albus                S------------DA------TTSTPEEEEEE-------EESASTSQESEDKESQSSEEA
AY651247.1:sparus_aurata                       EEESTSTS-----DATTSESESSSTPEEEEEESASTASEDDSTSTSQESEDEESQSSEEA
NW_019174336.1:seriola_dumerili                EEATASTSEASASDATASESESSSAPQEEEEED-SASTASDSASTSQESEDEESQSSEEA
JN854244.1:oreochromis_mossambicus(partial)    ------------------------------------------------------------
NW_016094914.1:kryptolebias_marmoratus         EEES-----------TAGESESSSTPEEEEEESAST---------TAESDDEESQSSEEA
NW_015443488.1:nothobranchius_furzeri          EEAS-----------TTSESDSSSTPEEEGEESASST----SSSASAESDDEESQSNEET

NC_030431.1:Ictalurus punctatus                TTAATATDSNSSESDESEETNT-----ADQPALLKNENPEVLIA-
NC_007121.7:Danio rerio                        TSTPGAADSESNSSQSTESTESQESDSDE-ETTQTTEASIIIVK-
NM_001124267.1:Oncorhynchus mykiss             TARPGAADSDSAESNESHDSDSDSAEEAATVATITTDAPIVITAK
AF223388.1:salvelinus_fontinalis               TARPGAADSDSAESNESHDSDSDSAEEAATVATITTDAPVVITAR
NW_018128285.1:monopterus_albus                TVTPGAADSDSDESDSDE---S------EGTGPDATTDTPLVITAK
AY651247.1:sparus_aurata                       TATPGAADSDSDESDSAG---SDS-DEQAAGPDTTTDMPVVITAK
NW_019174336.1:seriola_dumerili                TATPGAADSDSDESDSKE---SDS-DEQGAGPDTTTDMPVVITAK
JN854244.1:oreochromis_mossambicus(partial)    ---------------------------------------------
NW_016094914.1:kryptolebias_marmoratus         TATPGASDSDED--DSVE---SDS-DEEAVGPDVTTDMPAVVTAK
NW_015443488.1:nothobranchius_furzeri          TATPGAADSDSDESESSE---GDS-NEEGEGPDATADMPAVLTAK
```

Table S1.15: Osteopontin in Fish. Alignment after elimination of KX833902.1 (*Polypterus senegalus*) and XM_006007328.1 (*Latimeria chalumnae*).

Table S2: Alignment of the osteopontin amino acids by taxonomic groups. 2.1. Canonical sequences were derived from each group in Table S1. They were aligned with Clustal Omega, followed by manual adjustments. These adjustments reflect investigator supervision to override the rigid gap creation and gap extension rules of the program and bring common sequences together. The signal sequence (light gray) and the preserved GRGD motif are framed. The transparent blue boxes highlight the SSEE motifs; the transparent green box depicts the histidine-rich, variable-length domain in Reptilia and Aves; the (Q/E)TLP(S/D) sequence marks the beginning of the central domain and is shown dark purple background; the poly-aspartate sequences are on yellow background; the transparent red box covers the GRGD domain with the upstream sequences; the transparent dark purple box shows the potential thrombin cleavage site and heparin binding site; a second heparin binding site in primates (RL(Y/H)KRK) is marked by a light purple box (with a conserved stretch upstream in very light purple); between the heparin binding sites is a conserved domain of unknown function that is shown in a transparent faint orange box; the gray boxes depict the C-terminal domains. The colors match those in Figure 1. Common polymorphisms are indicated by lower case letters in smaller font. Lower case x indicates any amino acid. Variable inserts are within parentheses. **2.2.** From an alignment of all sequences retrieved, shown are two representatives of each group (4 representatives for 2 subgroups of Aves). The horizontal lines separate three clusters of distinct osteopontins. The birds and reptiles on top have high internal similarity. The Fish sequences (middle) are very different from all others. Eutheria and Prototheria (bottom) display a pattern that is internally similar, but clearly distinct from Fish and from Reptilia/Aves. The box covering all entries at the beginning of the alignments represents the putative signal sequence. Among the birds and reptiles, differences include a far N-terminal histidine-rich insert in the Squamata (boxed), a deletion (2nd small box) and a short substitution (3rd small box) in a subset of birds. Cervidae and Bovidae lack a sequence of about 20 amino acids in the downstream part of osteopontin (box across two lines). Reptilia,c+t = Crocodilia and Testudines, Artiodactyla,c+b = Cervidae and Bovidae, Afro+Xen = Afroteria and Xenarthra, Artiodactyla,ccs = Camelidae Celacea and Suidae.

```
Fish                    MKavAivVFVLLFATVLCRPav rik rk sv psaxSSsep-sedSSEEVVRrqp rp ap vpPALRKQ-------------
Prototheria             MRTAVICLCLISIACALPVKRSGPEANSGSSEEKQLYNKH--------------------
Marsupialia             MRTAVICFCLLGIVSALPVKQ---QINSGSSEEKxLYSKH--------------------
Carnivora               MRilAVICFCLLGIAYTIPIK----QtapDSGSSEEKQLYnhKY--------------------
Artiodactyla(b)         MRIAVICFCLLGIASALPVK----PTSSGSSEEKQLNNKY--------------------
Rodentia                MRlfiAVICFCLfliGIAsySLPVK----vq atDSGSSEEkrXhlYsnKhl--------------------
Artiodactyla(a)         Mkr ilAVICFCLLGIASALPVgk----QTdnSGSSEEKQLnysNKY--------------------
Lagomorpha(+similar)    MRIAVICFCLLGimAYALPVK----hqxDSGSSEEKQLYxKY--------------------
Eutheria(various)       MRxxVICFCLLGxxSxxPVKx---QatDSGSSEEKQLYNKY--------------------
Afroteria,Xenarthra     MRIAVICFCLLGIAYALPVKx---QADSGSSEEKQLyhSKY--------------------
Perissodactyla          MRIAVICLCLLGIAYALPVN----QADSGSSEEKQLYNKH--------------------
Chiroptera              MRIAVICFCLlvGIAYALPVK----HADSGSSEERQLyfNKhy--------------------
Primata                 MRIAVIcsFCLLGIatYALPVK----QADSGSSEEKQLYNKY--------------------
Reptilia(b)             MKIAVLicLCLFTviAFALPISKSRHHDDSrgmSAERKHDSREnh eh ahHAHHHHHHnhSHSHEHSQE
Aves(1)                 MKLALLCLCFISIAAAWPVSKSKQHAISASSEEKYDPRS---HHatHRYHQ---DHVDSQ-
Reptilia(a)             MKtiAVLCLCLIgsIAFALPVSKSKHhrq pa diSreSSEErn dhDSRS---HHshHRHHH---QrhVqh ti qe-
Aves(3)                 MKveAVLCLCLISITAAWPViv qpSKqhHAISASSEEKYDSRG---HHLhrRYHnd---Dhy vmeNSQ-
Aves(2)                 MKVAVLCLCLISITVAWPVSKSKQHAVSASSEEKYDSRG---HHSHR----HHHHVNSQ-
Aves(4)                 MKvaAVLCLCLISiv tav atvAWPVsnKSKqkHAitvSASSEEKYDpas rkG---HHslHRyqh hqrH(HH)hdHvmNSqh--
                        *:   :: . ::      *           *:*.
```

Fish	---AxVva pe kqlXre vapAPVQNVvdAAAAxatGSDESstk ed stSedEDEGav adPevtXXXXXXXXXXXtd apXst sd sd
Prototheria	--PNQLSSWLNTDPSQKQALLA-----PQNLVSSEESKE----------NIQQQTLPSIS
Marsupialia	--PNFVATWLNADPSQKQTLLA-----TQNSVSSEESTE-----------DLQETLPxNS
Carnivora	--PGAVATWplKPDPSQKQTFLA-----LQNatVLSeqEtnDD-----------FkqQKTLasSKS
Artiodactyla(b)	--PDAVATWLKPDPSQKQTFLate-----PQNSVSSEETDD-----------NKQNTLPSKS
Rodentia	--pt edAvim as tsWLvekPDPSQKQNLLA-----PQNxVSSEEtkDD-----------lfKQETLPSNS
Artiodactyla(a)	--PDAVATWLKPDPSQKQTFLae-----PQsnTaivSSEET(D)dn-----------nfKQETLPStkS
Lagomorpha(+similar)	--PDAvlATWLKPDPSQKQNLLx-----PQxAVSSEEtkDD-----------LKQETLPSKS
Eutheria(various)	--PDAVATWLKPDPSQKQTLLA-----PQNatVSSEETxD-----------xKQETLPSKS
Afroteria,Xenarthra	--PDAVATWLKPDPSYKQitLLA-----PQNAVSSEETDx-----------LKQETLPSKS
Perissodactyla	--SDAVSIWLKPDPSQKQNLLA-----PQT-VSSEETDN-----------LKQETLPSQS
Chiroptera	--PDAVATWLKPDPSQKQsnLLA-----PQNatVSSEETDdn-----------FKQETLPSqktS
Primata	--PDAVATWLknPDPSQKQNLLA-----PQNAVSSEETndD-----------FKQETLPSKS
Reptilia(b)	HgvNSHEHSQESpqEH--(EQQ)avSS-----QLNSQSlsEde rsDE----------ITEQQTLLavSS
Aves(1)	-------SQEHLQQTQNDLASL-----QQTHYSSEENAD----------VPEQPDFPDIP
Reptilia(a)	-------SRVsrPpaXatQEVhlVplP-----QQTSFSSEESVE----------Vpt ek qrQxxxxx(A)
Aves(3)	-------SQESQQHPQSDLASS-----QQTLyhSSEESvmD----------VPevQLHFPDVS
Aves(2)	-------SxESLLxPRDDLASP-----QQTLYSSEERAV----------VPVxxRFPxVx
Aves(4)	-------SWEsr lqQHptsQndDLvamSP-----QQTLYSSEESvmD----------VPve qkPhqFPDVS
	*:

```
Fish                     TASVNSqkDS-DDDDDDxxDDDDEtaEESeaXdes------------ae de se des ed ds sd sedSSESGESstTap
Prototheria              ---NESHDDVDDADDQDDSD--------------------------HKD---ESDDSDES
Marsupialia              ---xESPDDIDDxDDDDGDxG------------------------HKST---DSDDSDES
Carnivora                ---ndESHde dv vdDEDDegrDD-------VDSQDSVDShnD-----------tvDD---DSNQSDES
Artiodactyla(b)          ---NESPEQTDDLDDDDdeNSQ--------deVtnSdnDSDDat de nt ptDDpsDHSdn---ESHHSDES
Rodentia                 ---NESHDHMDDvdDDDDDDDGDhq av ednSZqeDSVDSDdeSDdeDDhdPDDsd hd he spD---deShdHSDES
Artiodactyla(a)          ---NESPdeHTDdtVDDde deDdeg dt vsh---dqv ds----QdvadXXXXXXdn hd tas dnDtaDrhSD---ESHHSDES
Lagomorpha(+similar)     ---xESHDHtmDDviDde de deDxdnH---VDSxDSxDxDDD-xxxxxDDSHHSD---ESHHSDES
Eutheria(various)        ---NESxDHTDDavDDEDDGDH---qvDS---xxSDDSxDxDHxDDxDHSD---ESHHSDES
Afroteria,Xenarthra      ---NEShqDQTDDVDDEDDgsDH---VDSQDSVdeSDDSDDDDHTDDPDHSD---ESHHSDES
Perissodactyla           ---NESHDHTDDVDDDDvdGDH---EDDQDSIDSDDSDETDPTDDPDNSD---ESHHSDES
Chiroptera               ---NESqhDlhTDDVDDdeDDEDH---VDSQDshIDSndDSDDadDhr tpDDspDNSD---ESHHSDES
Primata                  ---NESHDHMDDVDDedDDDDH---VDSQDSIDSNDSDedVDDTDDSHQSD---ESHHSDES
Reptilia(b)              ---SKSnhEDDDdnHSDVsd kdDDD----------------------HxDxxD---DSDESDES
Aves(1)                  ---SKSQEAVDDDD-D------------------------------DDN---DSNDTDES
Reptilia(a)              ---SvrSntEsd ed(G)Dhd---------------------------------hdDnd---DSNDTDES
Aves(3)                  ---SKSHEDVDDDDDD------------------------------DDN---DSNDTDES
Aves(2)                  ---SKSHEDPDDD--V------------------------------DDN---DSNDTDES
Aves(4)                  ---SKSHEDVDDD--dn------------------------------DDnd---DSNDTDES
                               * :  .:                                           .* .:.**
```

```
Fish                       APxTval tsPVIviTxEPVpavEptTvm da ptIlev paTIV-----TDxDTGRGDsn lmy(G)GYPgsDY(K)KSIviYVed edt
Prototheria                ------DEVVTDFPTDVPATAVFTPAAP----TRGDNGGRGDRVYR-GLKTKPGVLYKA-
Marsupialia                ------DEVVTDFPTDTPxTSSFLPDGP----TRGDNGGRGDSVAY-GLRSKVGAPYRS-
Carnivora                  ------DELVTDFPTDvifPATQFFTPAVP----TRDSynDGRGDSVAyh-GLRSkrSKKSHRY-
Artiodactyla(b)            ------DEva--DFPTDIPTtiAVsfTPpf ifP----TEns taNDGRGDSVAY-GLKSkrSKKFRRS-
Rodentia                   ------DexvfiTxsaPTnd gt pt qe ta epd vtFTPviaVP----TVed tvi pyDGRGDSvlAY-GLRSrkSrk sk lf hyq viS-
Artiodactyla(a)            ------DEVVTDFPTDivtPATasVFTPapVP----TegDtp qyn deGRGDns vi agvY-agLRtsKSKkmFrhRS-
Lagomorpha(+similar)       ------DElvxTDSPTdexPATPVFTPavVP----TxDTYDGRGDSVAY-rgLkrSKSKxflxxS-
Eutheria(various)          ------DEVVTDFPTDVPATPVFTPAVP----TGDTYDGRGDSVAY-GLRSKSKKLYRS-
Afroteria,Xenarthra        ------DELVT(DFPT)DdvPATPVFTPAVP----TVDTYDGRGDSVvaY-rgLRSKSkrKIxxS-
Perissodactyla             ------DELVTDFSTDVPATPVFTPAVP----TRDTYDGRGDSLSY-GLKSKSRKFRRS-
Chiroptera                 ------DELVTDFPTDflPGTPfvi avfTPAVP----TriDTnyDGRGDSVAY-rgLkr lsKSKKflHRS-
Primata                    ------DELVTDFPTDLPATEVFTPVVP----TVDitYDGRGDSVxY-GLRSKSKKFRRP-
Reptilia(b)                ------HEtnv fvTDxPTqd asPvfTx(PVxP)Dsp fv----------TGRGDvsAPY-GifRAKVDlq mv sh---
Aves(1)                    ------DEVVTDFPTEAPVT------PF----NRGDNAGRGDSVAY-GFRAKAHV-----
Reptilia(a)                ------DEDAVTDFPTDafPVTte pt lf ptPaf lpsP---TRGDNSGRGDSVagY-RMRAKAKLV(DVY-
Aves(3)                    ------EEVVTsiFPTDIPVTEPFPTFPF----TrqGDNAGRGDSVAY-RMrkAKA(ALLRAKA)
Aves(2)                    ------DEVVTxFPTDAPVTAPFPPYPF----TRGDNAGRGDSVAY-RIKAKAxV-----
Aves(4)                    ------DEVVTxFPTDIPVTvaPFPPFPF(GDSA)TRGDNasGRGDSVAY-RxRAKAats x-----
                                       .  *  .                ****            :
```

```
Fish                      kn snt yi heKvgPvsgPYKSYegFVgdTGK(LTGKK)KstmAYDMTDGNEVEKsl pmlKVYKALQVHsd dl limLEEDTST
Prototheria               ----------------------------------------------AVQGHDAS-DDF-T
Marsupialia               ----------------------------------------------SEQVHDVTEEDL-T
Carnivora                 ----------------------------------------------EdvQYPDSTEEDfl-T
Artiodactyla(b)           ----------------------------------------------snVqeSPDATEEDF-T
Rodentia                  ----------------------------------------------DaeqQyfd psDATDEDL-T
Artiodactyla(a)           ----------------------------------------------Evia qe hyq pl deA(TEEDV)-tm
Lagomorpha(+similar)      ----------------------------------------------xxQYPDAxEEDL-x
Eutheria(various)         ----------------------------------------------xviQYPDATEEDL-T
Afroteria,Xenarthra       ----------------------------------------------VIQfyPDATEEDL-T
Perissodactyla            ----------------------------------------------EDQYPDATEEDL-T
Chiroptera                ----------------------------------------------avVQYPDATEEDL-T
Primata                   ----------------------------------------------DIQYPDATDEDI-T
Reptilia(b)               ----------------------------------lf egk ksSxKSYKKSGKFDShqDVstDed fvDST
Aves(1)                   -----------------------------------VKASKLRKAARKLIEDDATAEVG--
Reptilia(a)               HKE)---------------------------------KSSkr hlYqkATGKFIVHDavTEEDDST
Aves(3)                   ---------------------------------atLLKsy ivKLhyKAAKK-LilYDATEEDESdy
Aves(2)                   -----------------------------------VKPSKLHKAARKLIVHDATEEDESA
Aves(4)                   -----------------------------------xKspa siKLrhKAAKKLimxYDAtsEEDESadt
                                                                              *   :
```

```
Fish                       --PEVESQGLDASSGtk saQDxxxqrsxraeQA-------Slv ap es eqEsrTsp st seSPE------------
Prototheria                --SRLESLESDESP---EAYPDAHKLQ-----KSSEWH--SNEASHQDDRSMQKSSEWHS
Marsupialia                --SQMESYESEKxH---KAFPLSQNLP-----KVSAWx--SNSKESNE------------
Carnivora                  --SLVKSxSmtEDDF---NAVLLShqrTVR-----gr st spDrgD--SHvaKDSQE------------
Artiodactyla(b)            --SHviESEEMHDAPKK--------------------------------------------
Rodentia                   --SHvm kenSxEsl--ls sk de at hlKav vi lpVAhq rhlLnsk-----vmPSDwqD--ShntGKtdSHE------------
Artiodactyla(a)            --stHVESEEavt dg deg at ph---KAIlf vdAQrgLH-----xASDWdn--Srq gweKDSQE------------
Lagomorpha(+similar)       --SHvm deSxDlmDDae ph---KAIplVAQahLqn-----xptSDWD--Sqh qe kgDSHE------------
Eutheria(various)          --SHMESEELDDAph---KAIxVAxRLx-----RASDWD--SHgpKDSQE------------
Afroteria,Xenarthra        --SQMESKELDDAH---KAIxVxQSLK-----KVSDWD--SQQQDShrE------------
Perissodactyla             --SPVESKDIDDVH---KAVLVAQGLH-----VASDWD--SRGKDSQE------------
Chiroptera                 --SHvmESkdeEmvDDArh(KAV)krAvi lfVAQGLphq-----VASDWD--ShprGKDSQE------------
Primata                    --SHvmESEELNGAY---KAIPVavQxLN-----vaPSDWD--SrhGKDSHE------------
Reptilia(b)                --PDvdESHEsyxxsa----KArhSL-----ES-Hed(TS)VExDDKSndLRDSNE------------
Aves(1)                    ----------------------------DSQlpAGLWWPKESREQDSRE------------
Reptilia(a)                --PDatESQQVDsyS----Kap qlPAAHhl fvPGKsyNI-SmvEWDDKSnhMqkDSNE------------
Aves(3)                    --MDADSQ-RSvlS----REDspASRrsSLRKHAS-SVVWSEqrSHGRDSSE------------
Aves(2)                    --LAADSHQAGLS----REDLGAHRSAGKYAD-SGEWGDKSHGxDSNE------------
Aves(4)                    --LDADSQqr vatGlfS----qrwEDspaAasv hr hrSLGKHats iv-SgrvEWasDKSHgreQDSSE------------
```

```
Fish                      ---saQkdA-tk tasSESedSstSTPEEEEet esSASS---xsgxSedStaSTSqaEsenEDE--ESQ------S
Prototheria               SEASHQDDRSVETHSHEEAKGYRLKQEDH---SSQQDDLNDSQESYK---VSR---ENDS
Marsupialia               --ASHPDEYSVETxSHEQLKSYQLExNxY--DSQQQSDSHGSQENDK---VSQ---EFHx
Carnivora                 --TSQVDDHSMETKSrh raHsta krEYKLkrASDE---nsNKHSHEIGSQEsnSde---VSS---ELVgs
Artiodactyla(b)           --TSQLntDHSkeETNSnsdELSKELktmPKAKDek----snKHSdn vlrIESQENSK---vlSQ---EFH-
Rodentia                  --SSQLDde phSVEtsHShqlEQsp rkqEhyKrqKArs hd enSxESats ed qhlSDVIDSqkr ae sg sa rk---vaSql---Ehlf qhS
Artiodactyla(a)           --TSQPdsDrhSmvETHSrqhEHSekEfyKLKAedyDedg---sg dny reHSDVIdeSQdeNSkq---VSpqh---EvfHS
Lagomorpha(+similar)      -xxSQxDDHSvmETqhSxEQasKqeYKrlKAxDx---SxEHSDSIDSQESSK---vsSqhl---EfsHS
Eutheria(various)         --TSQLDDHSVETHSHEQS---KLKAxDE---SNERSDVIDSQENSK---VSH---EFHS
Afroteria,Xenarthra       --TSQplDDHSVETHSQEQSKEYKLKANDve---SSEHSDxIDSQEndSK---VSQ---EFH-
Perissodactyla            --TSQLDDHSVETHSREHSKEYQLKANDE---TSEHSDVIDSxENSK---VSQ---EFPS
Chiroptera                --TSQLDDHStvETS hyShr ke lha asp keEYKLKAnmDae---SngeHsa dv vgIDSQENSkr---VSH---EFHS
Primata                   --tmSQLDDqhSAETHSHke qhSRLyhKRKASDde---SNEHSDVIDSQExSK---viSrh---EFHS
Reptilia(b)               -VHSASHDrktSLEnd nhSqpr eqKplESVEDsd(HDDAD)HDnhSKav ts viSLESTESrgtE---qlKK-------
Aves(1)                   ---LAQHQSVENDSRPrkFDSPEVDGGD------SKASAGV-----------------DS
Reptilia(a)               -VtnSRSRDdk(SMEND)SqhQqrLdeSVEVDrsDnd-----SknPDVtpEDSHMSNEStrEQqh qhTQ-----
Aves(3)                   -QDSdn pl hr hdRSLENdnSrw phKSDSHEAEGDS-----SKSGVRGDSlh spSVEst krERGDShl psSvaES
Aves(2)                   -VVRKLPDRSLENDSRQKFDSPExExxS-----SExGARGxxxxSRE-----------S
Aves(4)                   -LDSkn ql rhp dh rqwSmlwENDSrqQKflDShrEvaEgrDD(D)----SKas gsVRGDSHqhpSmvlE-----------S
                                       : *. *           . .
```

Fish	SEEATATPGAADSDS-------DESD$_{se}$SEx$_{st}$xSDSxDE$_{qae}$ATG$_{pa\ dt}$x---------------
Prototheria	QEKT---------------------------------AQDA-------------------
Marsupialia	xEVD-----xDSQEFH-----KQQVNKLSQEFxSQEVHLVN-------------------
Carnivora	-----------------------------Q$_{ti}$VQS$_{nsh}$EKE$_{lr}$V$_{lq}$-------------------
Artiodactyla(b)	---------------------------------SLEDKLDL-------------------
Rodentia	-----------------------------H$_{ek}$FHS$_{hq}$E$_{fd}$K$_{ls\ vh\ lep}$-------------------
Artiodactyla(a)	-----------------------------QE$_{lf\ ph}$S$_{hr}$EDKLV$_{lp}$-------------------
Lagomorpha(+similar)	-----------------------------$_{rq}$EFxSHEDKLxI-------------------
Eutheria(various)	-----------------------------QE$_{fs}$HSxEDKLVx-------------------
Afroteria,Xenarthra	---------------------------------SHEDKHxP-------------------
Perissodactyla	-----------------------------QEFHSSEGKLVR-------------------
Chiroptera	-----------------------------$_{rq}$E$_{lf\ hqd}$S$_{rh}$EDKL$_{av\ ls}$-------------------
Primata	-----------------------------HEFHS$_{hq}$EDMLVV-------------------
Reptilia(b)	---------D$_{rh}$SEELQQ$_{lv}$D$_{sh}$DHDVDDVSNQ$_{ts}$LES$_{ta}$ESHHSVEDHHSAEDHHSxEDHHSxE
Aves(1)	RESQGSV---------------PAVDT---------------------------------
Reptilia(a)	---------I--(EDLQQ)$_{vs\ da}$SVPE$_{tv\ nd}$AD---------------------------------
Aves(3)	$_{rw}$E$_{sr\ rg}$DSHPS$_{va}$ESRES$_{qr}$DRVSAELSDDI---------------------------------
Aves(2)	RESx----------AxVxx-xxxxxDN---------------------------------
Aves(4)	$_{rk}$E$_{sr}$Q---------(xx)V$_{hr\ vi}$SAEx$_{ps}$PDDN---------------------------------

Fish	---------------------TTDmaPVVITAK
Prototheria	DSDEFNRKHYLKSHTSHEFDSASSETH-----
Marsupialia	DPESVENxKxLKLHSxxExDSASxExH-----
Carnivora	dhSekSevEeqDKHLKFrh viSHELDSASSEVN-----
Artiodactyla(b)	DHKSE-EDKhrLKIRISHELDStaSSEVN-----
Rodentia	DPKSKEde dteXyhLKifmRivSHEli edSsaSSEvaN-----
Artiodactyla(a)	dn plsKSE(E)EDtkHLKflRVSHELDSASSEVN-----
Lagomorpha(+similar)	edPKSxEEde keHLKFRVSHELdeSatSSEaviN-----
Eutheria(various)	DPKSE-EDKHLKFRVSHELDSASSEVN-----
Afroteria,Xenarthra	DPKSEEEDKHLKFRtiSHELESxSxExN-----
Perissodactyla	DRKSEDEDKYLKFRTSHESESASSEVN-----
Chiroptera	DPqkSEEQDKHLKFRISHELESASSEVN-----
Primata	DPKSKEEDKHLKFRISHELDSASSEVN-----
Reptilia(b)	DHHSVExxSxEDHHSVEdhHdhSivEDNEVitL---
Aves(1)	-----SNQTLESAEDAEDRHSIENNEVTR---
Reptilia(a)	-----SNQTAEStavEDIQDHHSilEDNEVti il---
Aves(3)	-----SNQTLESAEDSQDRHSIESNEVTL---
Aves(2)	-----SNQTxESAEDxQDxxSIKxNEVxx---
Aves(4)	-----SNQTLESAEDas qrDhrHSIENNEVTL---

Table S2.1. Alignment of canonical sequences.

```
XM_015411327.1:Gecko japonicus(Reptilia,Squamata)             MKIAVLCLCLFT----IAFAIPIKKTKFHDG-AGSSEDNHDSRGHHVHKHRHHHHHSHSQSQEHSPEHVNSHES----QEH---------Q---LP-
NW_013658771.1:Thamnophis sirtalis (Reptilia,Squamata)        MKIAVLTLCLFT----VAFALPISQSRDHDN-SMSAESKHDSKENEAHAHHHH--HHHSHSH----------EHSQESPEH---------HSQESS-
XM_003205565.3:Meleagris gallopavo (Aves)                     MKLALLCLCFIS----IAAAWPVSKSKQHAI-SASSEEKYDPRSHHAHRYHQ--------------DHVDSQ--SQEHLQQT-------RNDLASL-
NM_001323234.1:Coturnix japonica (Aves)                       MKLTLLCLCFIS----ITAAWPVSKSKQHAI-SASSEEKYDPRSHHTHRYHQ--------------DHVDSQ--SQEHLQQT-------QNDLASL-
XM_010218705.1:Tinamus guttatus (Aves)                        MKVAVLCLCFIS----ITIAWPLSKSKQHAV-SASSEEKYDARGHQSHRHH----------------HVTSQ--SLENLLLP-------REDLALP-
XM_013190510.1:Anser cygnoides domesticus (Aves)              MKVTVLCLCLIS----VAAAWPI-KSKQHAI-SASSEEKYDPRGHHSHRQHH--------------DLVNSH--SQELLQHP-------QNDLASL-
NW_017728948.1:Crocodylus porosus (Reptilia,c+t)              MKIAVLCLCLIG----IAFALPVNKSKHQPD-SRSSEERDDSRSHH--RHHH---------------HVQIE--SRVSPPRA-------QEVHVLP-
XM_007066639.1:Chelonian_mydas (Reptilia,c+t)                 MKIAVLCLCLIS----IAFALPVSKSKHHAI-SESSEDNHDSRSHHSHRHHH--------------QQVHTQ--SRVRPAKT-------QELLVPP-
NM_001124267.1:Oncorhynchus mykiss (Fish)                     MKAAIVFVLLFA----TVLGRPAIRSPGNNP--ESLEELVKPAPVLGIAP------------------------------AELTEVAPVQNVDTAT-
NW_018128285.1:Monopterus_albus (Fish)                        MKVAIVTVLLFA----TVLCGPVRKASYRSS--ESSEEVVRRPAVPAIRKQA--------------------------AVVPQSRAAHVQNVVAAAA
XM_001370458.3:Monodelphis domestica (Marsupialia)             MRTAVICFCLLG----IVSALPVKH----QINSGSSEERRLYN-KHP------------------------------NLVATWLNADPSQKQTLLA-
XM_003772957.3:Sarcophilus harrisii (Marsupialia)             MMRTAVICFCLLG---IVSALPVKQ----QINSGSSEEKQLYS-KHP------------------------------NFVATWLNADPSQKQTLLA-
XM_004681418.2:Condylura cristata (Lagomorpha et al)           MRIAVICFCLLG----IVSALPVKQ-----GDSGSSEKEQ---------------------------------------------------------
XM_003985184.5:Felis catus (Carnivora)                        MRIAVICFCLLG----IAYAIPIKQ-----TDSGSSEEKQLYN-KYP------------------------------VAVATWPKPDPSQKQTFLA-
XM_021702336.1:Neomonachus schauinslandi (Carnivora)          MRLAVICFCLLG----IAYAIPIKQ-----ADSGSSEEK----------------------------------------------------------
XM_020876412.1:Odocoileus virginianus texanus (Artiodactyla,c+b) ----------------------------------------LNN-KYP------------------------------DAVATWLKPDPSQKQTFLT-
XM_005972457.1:Pantholops hodgsonii (Artiodactyla,c+b)        MRIAVICFCLLG----IASALPVKP-----TSSGSSEEKQLNN-KYP------------------------------DAVATWLKPDPSQKQTFLA-
XM_012523397.2:Dasypus novemcinctus(Cingulata)                YARPCTRNTLTLSSFFIFSVSKVKH----QADSGSSEEKQLYD-KYP------------------------------DAVAAWLKPDPSQKETLLA-
XM_007951712.1:Orycteropus afer afer(Tubulidentata)           MRIAVICFCLLG----VASTFPVKR----QADSGSSEEKQLYN-KYP------------------------------DAVATWLKPDPSHKQTLLA-
NW_001711695.1:Ornithorhynchus anatinus(Prototheria)          MRTAVICLCLIS----IACALPVKRS--GPEANSGSSEEKQLYN-KHP-----------------------------NQLSSWLNTDPSQKQALLA-
XM_003790097.3:Otolemur garnettii (Primates)                  MRLAVICFCLLG----IAYTLPVKH-----VDSGSSEEKQFYN-KYP------------------------------DAVATWLKPDPSQKQNLLA-
NW_016536240.1:Manis javanica (Afro+Xen)                      MRIAVICFSLLG----IACTLPVKQ-----ADSRSSEEKQLYQ-KYP------------------------------DAVASWLKSDPSHKQTLLA-
D11411.1:Oryctolagus cuniculus (Lagomorpha et al)             MRIAVICFCLLG----MAYALPVKH-----ADSGSSEEKQLYH-KHP------------------------------DALATWLNPDPSQKQNLLT-
XM_006198167.2:Vicugna pacos (Artiodactyla,ccs)               MKLAVICFCLLGI----ASALPVGQT-----NSGSSEEKQLNN-KYP------------------------------DAVATWLKPDPSQKETFLA-
XM_014787875.1:Ceratotherium simum simum (Perissodactyla)     MRIAVICLCLLGI----AYALPVQQ--A---NSGSSEEKLLYN-KSP------------------------------DAVGTWLKPDPSQKQTLLA-
XM_014842441.1:Equus asinus (Perissodactyla)                  MRIAVICLCLLGI----AYALPVNQ--A---DSGSSEEKQLYN-KHS------------------------------DAVSIWLKPDPSQKQNLLA-
XM_016213045.1:Miniopterus natalensis (Chiroptera)            MRAAVICFCLLGI----AYALPVK--RA---DSGSSEERQLY--KHP------------------------------DAVATWLKPDPAQKQNLLA-
KB104739.1:Myotis davidii (Chiroptera)                        MRIAVICFCLLGI----TSALP--VKHA---DSGSSEEKQLYN-KHP------------------------------DAVATWLKPDPSQKQSLLA-
XM_012559624.2:Trichechus manatus latirostris (Afro+Xen)      MRIAVIYFCLLGI----AYALPVKHQ----ADSGSSEEKQLYS-KYP------------------------------DAVDTWLKPDPSYKQILLA-
JH171653.1:Heterocephalus glaber (Rodentia)                   MRIAVICFCLIGI----TYSLPVN-Q----ADSGSSEEKPLYN-KLP------------------------------EAVSSWLKPDPSQKQNLLA-
XM_020154612.1:Castor canadensis (Rodentia)                   MRIAVIFFCLLGI----AYSLPVK-Q----PDSGSSEEKQLFNNKHA------------------------------DAVAIWLKPDPSQKQNLLA-
NM_001040058.1:Homo sapiens (Primates)                        MRIAVICFCLLGI----TCAIPVK-Q----ADSGSSEEKQLYN-KYP------------------------------DAVATWLNPDPSQKQNLLA-

Gecko japonicus                  SHQSSDSDES-EEITEQQTL---LVHPD------KSAEDDHSDVDDHDDVDDHDDVDD------------DSHGEADDSDESHESDDSHQ-VVTEVPTDRP-FT---PSAAPG---PF----
Thamnophis sirtalis              QLNSQSSEDR-DEITEQQTL---LASSS------KSHEDD----DDHSDVSKDD-------------------DHSDDSDDSSESDESHE-TVTDAPTEAP-YT--------E---SV----
Meleagris gallopavo              QQTHYSSEEN-ADVPEQPDF---PDIPS------KSQEAV----------DDND-----------------------DDNDSNDTDESDE-VVTDFPTEAP-VT------------PF----
Coturnix japonica                QQTHYSSEEN-ADVPEQPDF---PDVPS------KSQETV----------DDDD----------------------DDDNDSNDTDESDE-VFTDFPTEAP-VA------------PF----
Tinamus guttatus                 QKTLYSSEER-ADVLVPERF---PAVFS------KSHEDP----------DDD-----------------------VDDNDSNDADESDE-VVTVFPTDAP-VT---AAFPPY---PF----
Anser cygnoides domesticus       QQTLYSSEENAENVPVKPHF---PDVPS------KSHEAV----------DDDN--------------------DDDDDNDSNDTDESDE-VVTDFPTDIP-VT---APSPPF---PF----
Crocodylus porosus               QQTSFSSEES-VEVPKRQIF----QVAS------RSTESE-----------D------------------------DQDDDSNDTDESDEDAVTDLPTDFP-VT---ETFPPF---PP----
Chelonian_mydas                  QQSHFSSEES-VENTEQQSL---PKPAS------VSNEDD----------GD------------------------HDDNDSNDTDESDEDIVTYFPTEAP-VT---TPLTPA---VP----
Oncorhynchus mykiss              -TSDESSDK-DETEGADPTS----DPTSDSADSADS--------------------A-------------D-SADSADSDDTDESSESEEADTTAAPATAE-------PTVEPTMATTEAP-
Monopterus_albus                 TDSDESKGSSDEDESVVTT-DGTNTALASDTTSDKSKDSG---DDDDD--DNDDETK--------------------ETEEDESSSESGES-STLAPATDTPVIITDEPVAETTVEPIVPTI
Monodelphis domestica            TQNSVSSEES-TEDLQQ-TL---PSNSN------ESPDTTDDVDDDD----DDDGHKS---------------------TDSDDSDESDE-VVTDFP----TD----IPTTSS-FLPDGPT-
Sarcophilus harrisii             TQNSLSSEES-TEDLQE-TL---PRNSS------ESPDDIDDEDDDD----GDH-NKS---------------------IDSDDSDESDE-VVTDLP----TD----TPATPS-FLPDGPT-
Condylura cristata               ------------------SL---PSVSD------ESN----DVDDVD----DGDNTESRDSTDSD------DDDHSDDSDESHHSDESDE-MATDSP----TD----LPATVD-YTPVVFT-
Felis catus                      LQNAVLSEET-DDFKQK-TL---ASKSN------ESHDVD----DED----DEDDVDSQDSVDSHDTD-----------DDSNQSDESDE-LVTDFP----TD----VPATQF-FTPAVPT-
Neomonachus schauinslandi        -QNTVLSQEN-DNFQQK-TL---SSKSN------ESHEDV----DED----DGDDVDSQDSIDSNDVD-----------DDSNQSDESDE-LVTDFP----TD----IPATQF-FTPAVPT-
Odocoileus virginianus texanus   PQNSVSSEET-DDNKQN-TL---PSKSN------ESPEQIDDLDDDD----DENSQDVTS----NDSDDTDNTDDSDHSNESHHSDESDE---ADFP----TD----VPTTAV-STPPIPT-
Pantholops hodgsonii             PQNSVSSEET-DDNKQN-TL---PSKSN------ESPEQTDDLDDDD----DENSQEVNS----DDSDD-ANPDDSDHSNESHHSDESDE---ADFP----TD----IPTIAV-FTPPFPT-
Dasypus novemcinctus             PQNAVSSEET-DNFKQD-TL---PSKSN------ESHDHTDNADDED----DGDHVDSRDSDDSNE---SDHNDDPDHSDESHHSDESDE-VVTDFT----TD----APATP-----VVPT-
Orycteropus afer afer            SQNTLSSEET-DDLKQE-TL---PSKSN------ESLDQTDDVDDED----DGDHVDSQESDDSED--H-DHTDDPYHSDESHHSDESDE-LVTDFP----TE----VPATPV-FTPAVPT-
Ornithorhynchus anatinus         PQNLVSSEES-KENIQQQTL---PSISN------ESHDDVDDADDQDD---------------------------SDHKDESDDSDESDE-VVTDFP----TD----VPATAV-FTPAAPT-
Otolemur garnettii               PQ----------------TL---PSTSN------ESHDHMDDEDDDD-------HVD---SHESDDSDDTDNPDDSHHSDESHHSDESDE-MV--------TA----NFPTDV-PTPTVPT-
Manis javanica                   LQNTVSSEET-DNFKQE-TL---PSRSN------ESHDDVDDEED-------GDNRDSQDSADASKSDDV------DHTDDPDDSDESDE-LV--------TD----VPATPV-FTPADPT-
Oryctolagus cuniculus            PQNAMSSEEK-DDLKQE-TL---PSKSI------ESHDHMDDIDEDED--D--DHVDNR---DSNESDDADHPDDSHHSDESHQSDESDE-VTV-YP----TE----DAATTV-FTEVVPT-
Vicugna pacos                    PQSTASSEET-DNFKQE-TL---PSTSN------ESPDLTDDVDDD----DD-DTVDSQD---VDSNDSDDHTDDTDRSDESHHSDESDE-VVTDF----PTD----IPATAV-FTPAVPT-
Ceratotherium simum simum        PQ-TVSSEET-DDLKQE-TL---PSKSN------ESPDHTDDVDDD----DDGDHVDSQDSIDSDDSDETDPTDDPDQSEESHHSDESDE-LVTDF----PTD----LPATPV-FTPAVPT-
Equus asinus                     PQ-TVSSEET-DNLKQE-TL---PSQSN------ESHDHTDDVDDN----DDGDHEDDQDSIDSNDSDETDPTDDPDNSDESHHSDESDE-LVTDF----STD----VPATPV-FTPAVPT-
Miniopterus natalensis           PQNTVSSEET-DDFKQE-TL---PSTSN------ESHDLTDDVDDD----DDSNHVDSQDSTDSNDSDDADQTDDSDNSDESHHSDESDE-LVTDF----PTD----FPGTLI-VTPPVPT-
Myotis davidii                   PQNTVSSEET-DDFKQE-TL---PSQSN------ESQDHTDDVDDD----DDEDHVDSQDSIDSNDSDDDDHTDDPDNSDESHHSDESDE-LVTDFPTDFPTD----FPGTPF-ATPAVPT-
Trichechus manatus latirostris   PQNAVSSEET-DDLKQE-TL---PSKSS------ESQDQTDSVDDE----DDGEQVDSQDPIDSDDTHEDDHTDDSNHSNESNHSDESDE-LVTDF----PTD----DPATSV-FTPAVPT-
Heterocephalus glaber            PQNSVSSEET-DDLKQE-TL---PSNSN------ESHDHMDDVDDEDD--GDQD---------SVDVDDDDHPDDSHHSDESDHSDESDE-VVTDA----PTN----GPETPV-FTPIVPT-
Castor canadensis                PQNVVSSEET-DDIKQQ-TL---PSNSN------ESHDHMDDMDDE----DDGDHVDSQDSVDSDDSDDTDHPDDSHHSDESHHSDESDE-LFTSS----PTD----NPGTQV-FTPVVPT-
Homo sapiens                     PQNAVSSEET-NDFKQE-TL---PSKSN------ESHDHMDDMDDE----DDDDHVDSQDSIDSNDSDDVDDTDDSHQSDESHHSDESDE-LVTDF----PTD----LPATEV-FTPVVPT-
```

```
Gecko japonicus                   -----TGRGDI--PYGIQHGQ-------------------------------------------LKFLRKSKKSSEQFDSHDTTQE-DDSLPDVDSHET------HSSKAVSHESQDTSTES
Thamnophis sirtalis               -----TNRGDVP-SDEFRPNVDFM---------------------------------------SLGRS-------GKFDSQSVSDE-VDSTPDDESHED--------SKARSLESHE----S
Meleagris gallopavo               NRGDNAGRGDSV-AYGFRAKA------------------------------------------HMVKASKLRKTARKLIEDDATAEA------GGSQ--------------------LAGLW
Coturnix japonica                 NRGDNAGRGDSV-AYGFRAKA------------------------------------------HVVKASKIRKAARKLIEDDATTED------GDSQ--------------------PAGLW
Tinamus guttatus                  TRGDNAGRGDSV-AYRIKAEP------------------------------------------AVMKPSKLHKAARKLMVHDVTEE-DLSALAADSHQEGFSREAFG-AHRSAEKYADSLEW
Anser cygnoides domesticus        TRGDNAGRGDSV-AYRMKAKA------------------------------------------SMVKSSKLRKAARKLIADDATEE-DESALHGDSQQVGLWQKELA-AHRAPGKRAVSGEW
Crocodylus porosus                TRGDNSGRGDSV-GYRMRAKAKLV------------------------------------------KSSRHYQATGKFIVHDATEE-DDSTPDTESQQVDYSKPLPA-AHLVPGKYDISVEW
Chelonian_mydas                   TRGDNSGRGDSV-AYRMRAKAKLV-----------------------------------DIY-HKEKSSKLYKALGKFIVHDVTEE-DDSTPDAESQQVDSSKAPCA-ACHFPGKFDMSMEW
Oncorhynchus mykiss               --IYDDGRGDSM-GYPSDYKKSIIYVDTNNIEKGPSPYKSYGKIDEGMYAGKKVSVYNTGLGNKIEKTMTVFKAL---QVHDLMEE-DTSTPDVESQVLDASSGKAEESSMRKAHVDVAS--
Monopterus_albus                  VTDADTGHGDNMRGYPGDY-KSIVYVEDKSYHKIPVAYKSYEFVGTGK-----KTAYDMTEGNEVEKSLKVYKAI--QVHSDLVEE-DTSTPEVESQGLDASSVTTQD--HQEEGASP-S--
Monodelphis domestica             R-GDNSGRGDSV-AYGLR-SKV----------------------------------------------GAPDISS--AETHEVTEEDLTSQ--MESYESEKAH---K-AFPLSQNLPKVSAW
Sarcophilus harrisii              R-GDNGGRGDSV-AYGLR-SKL----------------------------------------------GAPYRSS--EQVHDVTEEDLTSQ--IESYESEKTH---K-AIPLSQTFPKVSSW
Condylura cristata                R-DTYDGRGDSV-AYGLK-SKS----------------------------------------------IKFYSAE--DQYPDATKEDLASQ--MESREMRNGP---K-ATLVALNLQSTSDV
Felis catus                       R-DSYDGRGDSV-AYGLR-SKS----------------------------------------------KKSHRYE--DQYPDSTEEDFTSL--VKSQSMEDDF---N-AVLLSHTVRRSPDR
Neomonachus schauinslandi         R-DSNDGRGDSV-AHGLR-SRS----------------------------------------------KKSHRYE--VQYPDSTEEDLTSL--VKSESMEDDF---N-AVLLSQTVRGTSDG
Odocoileus virginianus texanus    E-NTNDGRGDSV-AYGLK-SKS----------------------------------------------KKFRRSS--IQSPDATEEDFSSH--VESEEMHDAP---K-K-------------
Pantholops hodgsonii              E-STNDGRGDSV-AYGLK-SKS----------------------------------------------KKFRRSN--IESPDATEEDFTSH--IESEEMHDAP---K-K-------------
Dasypus novemcinctus              E-DAYNGRGDSV-AYGLE-SKS-----------------------------------------------KLYRSK--IQFPDATEEDLTSH--MEREGLDDAH---R-VIHVARRLNRASDW
Orycteropus afer afer             V-ETYDGRGDSV-ASRLK-SKS----------------------------------------------KKIYRSV--IQYPDDTE-ELTSH--MRSKELDDVP---K-AISVVHVLKRASDW
Ornithorhynchus anatinus          R-GDNGGRGDRV-YRGLK-TKP----------------------------------------------GVLYKAA--VQGHD-ASDDFTSR--LESLESDESP---E-AYPDAHKLQKSSEW
Otolemur garnettii                E-NIYDGRGDSI-PYGLR-SKS----------------------------------------------KKFRRLD--IQIPDETQEDFTSY--VESEELGHGP---K-VIAFNQGLKVPSDL
Manis javanica                    G-DTNDGRGDSV-AYGLR-SKS----------------------------------------------KKFYRSA--LQYPDATEEDLTSQ--MESKEIDKAR---K-ATLVARSLRVPSDW
Oryctolagus cuniculus             V-ETYDGRGDSV-AYRLKRSKS----------------------------------------------KMFHVSN--AQYPGASEEDLSSH--VDSEDLDDTP---R-AIPVAQHLNVPSDW
Vicugna pacos                     EDT-QDGRGDNI-GYALR-TKS----------------------------------------------KKFRRSE--VEHPEAM-----TH--VESEEADEAH---K-AIFDAQGLHTASDW
Ceratotherium simum simum         SDT-YDGRGDSV-GYGPR-SKS----------------------------------------------KKVRRSE--VQYPDATEEDLTSH--VKSKEVGGVH---K-AILVAQGLHVASDW
Equus asinus                      RDT-YDGRGDSL-SYGLK-SKS----------------------------------------------RKFRRSE--DQYPDATEEDLTSP--VESKDIDDVH---K-AVLVAQGLHVASDW
Miniopterus natalensis            RDT-YDGRGDSV-AYGLK-SKS----------------------------------------------KKFHRPA--AQYPDATEEDLTSH--VESNEADEAH---K-AVLVAQGLHVASDW
Myotis davidii                    IDT-NDGRGDSV-AHGLR-LKS----------------------------------------------KKLHRSV--VQYPDATEEDLTSH--MESDEMDDAH---R-AVLVAQGLPVASDW
Trichechus manatus latirostris    VDT-YDGRGDSV-VYRLR-SKS----------------------------------------------KKIPRSD--IQHPHATEEDSISQ--MESKMLDDAH---K-AIPVISSLKKPFDW
Heterocephalus glaber             VETYD-GRGDSL-AYGLR-SRS----------------------------------------------KKFHISD--PQYPAATDKDLTSN--VESQESSDAQ---K-AVLVAHRLNMPSDW
Castor canadensis                 VEV-YDGRGDNV-AYGLR-SRYL---------------------------------------------KKFDISD--AQFSDATEEDLTSH--IESEESDDAL---K-AIPVAQHLKVPSDW
Homo sapiens                      VDT-YDGRGDSV-VYGLR-SKS----------------------------------------------KKFRRPD--IQYPDATDEDITSH--MESEELNGAY---K-AIPVAQDLNAPSDW


Gecko japonicus                   EDKS-NLQDSNEVHS----------------ASHDKSVENDSRQKL---DSVEEDH------DNSKADISQESDGIDHITELLKAR---------------------------RGEPQ----
Thamnophis sirtalis               DDKS-DLTDSNEVHS----------------ASHDISLEDHSPEKP---ESVEDS-----S-DHSKASLSLESTE---SREQKKDH---------------------------SEEPQ----
Meleagris gallopavo               WPKESREQDSREL-------------------TQHQSVENDSRPRF---DSPEVDGG-----D-SKASAG-----------------------------------------VDSRESQ----
Coturnix japonica                 WPKESREQNSREL-------------------PQHQSVENDSRPKF---DSREVDGG-----D-SKASAG-----------------------------------------VDSRESQ----
Tinamus guttatus                  GDKS-HGHDSNEVGR----------------KLPERSLEKDSRQKF---DSPERD-------S-GEWGARGDSPPS-----------------------------------RESRESQA---
Anser cygnoides domesticus        LDKS-HERDSSELTH-------------------HQSMENDSRQKF---DSPEVEGG-----D-SQDGARGDGRHS-----------------------------------LESRERRV---
Crocodylus porosus                DDKS-HMKDSNEVTS----------------RSRDD-----SHQRL---ESIEVDRD-----D-SNPDVPEDSHMSNESREQHHTQ---------------------------I--------
Chelonian_mydas                   DDRS-NIQDISEVNR----------------RSHDKSMENGSQQQL---DSVEADSD-----N-SKPDVTEDSHMSNESTEQQQAQ---------------------------TEDLQ----
Oncorhynchus mykiss               -----QDRTPSESPE----------------SQKAKSLESDA-------TSELTSSASSDGTSESTSTSASNETDSSNSSEEATARPGAAD--------------------SDSAESNESHD
Monopterus_albus                  ---------------------------------DA------TTSTP---EEEEEE-------EESASTSQESEDKESQSSEEATVTPGAAD--------------------SDSDESDSDE-
Monodelphis domestica             G---SNSKESNEA--------------SHP-DEY--SVETYSHEQF---KSYQLEETNP---------------D---SQQQG-----------DSHDS---QENDKVSQEFR---------
Sarcophilus harrisii              E---SNGQESNEA--------------SQA-DEY--SVETHSHEQL---KSSQLERNIY---------------D---SQQQS-----------DSHGS---QENDKVSQEFHNREVDRD--
Condylura cristata                D---SQGEDSFEK------------------RDD--SLENNGFEHS---QEYKLKAND----------------E---SSQ----------------------KTPESSHLSH---------
Felis catus                       D---SHVKDSQET--------------SQV-DDH--SMETKSRKHS---KEYKLKASD----------------E---NNKHS-----------HEIGS---QESSDISSELV---------
Neomonachus schauinslandi         D---SHAKDSQET--------------SQP-DDH--SMETKSHKHA---REYKLRASD----------------E---SNKHS-----------HEIGS---QENSEVSSELV---------
Odocoileus virginianus texanus    ------------T--------------SQL-NDH--SKETNSSELS---KELKPKAKD----------------E---SNKHS-----------DVIES---QENSKVS-------------
Pantholops hodgsonii              ------------T--------------SQL-TDH--SEETNSNELS---KELMPKAKD----------------E----SKHS-----------NLIES---QENSKLS-------------
Dasypus novemcinctus              D---SHGKDSQET--------------RQL-DDH--SVETHSHEQS---KSRKA--SH----------------E---SNEHS-----------DVIDS---RENSKVSHEFN---------
Orycteropus afer afer             D---SHPQDSHEV--------------SQLEISH--SVETHSHEQS---KELEA--KD----------------E---SNERS-----------DMIDS---QEISKVSHEFH---------
Ornithorhynchus anatinus          H---SNEASHQDDRSMQKSSEWHSSEASHQ-DDR--SVETHSHEEA---KGYRLKQED----------------H---SSQQD-----------DLNDS---QESYKVSRENDSQEKTAQDA
Otolemur garnettii                D---SHETSHDL------------------------STETHSK------------------------------------------------EPSDMIVS---QEHSHVSLEV----------
Manis javanica                    D---SQ-----ET--------------SQL-DDQSQSAETNSQGYS---KEYTLKAKD----------------E---SNEH-----------ADVIGN---KEDSKVSQE-----------
Oryctolagus cuniculus             D---SQEKDSHDV--------------SQV-DDH--SVETQSHEQA---RQYKREAND----------------N---SVEH-----------SHSIDS---QESSKVSQES----------
Vicugna pacos                     N---SQWKDSQET--------------SQP-GDH--SVETHSQEHS---EEYKLKADD----------------E---GDRHS-----------DVVDS---QENSQVSPEIHSQELP----
Ceratotherium simum simum         D---SRGKDSLET--------------SQL-DDH--SVETHSLEHS---KEYKLKAND----------------E---TSEHS-----------DVIDS---HENSKFSHERHSQEFH----
Equus asinus                      D---SRGKDSQET--------------SQL-DDH--SVETHSREHS---KEYQLKAND----------------E---TSEHS-----------DVIDS---LENSKVSQEFPSQEFH----
Miniopterus natalensis            D---SHGRESQEI--------------SLL-DDH--SVETHSLKLP---KEYQLKAIG----------------Q---SNEHS-----------DGIDS---PENVKISHEFHSQELG----
Myotis davidii                    D---SRGKDSQET--------------SQL-DDH--SVETHSHELA---KGYKLKAMD----------------A---SNEHS-----------VGIDS---QENSRVSQEFHSRELQ----
Trichechus manatus latirostris    D---SYQQDSRET--------------SQQ-DDH--SVETHSQEQY---KVYKLKAND----------------V---SSEHS-----------DTIHS---QEDSRVSDELH---------
Heterocephalus glaber             D---SRGKDSHES--------------SQL-DDP--SVETHSQEQS---REHKRKARN----------------D---SSEHS-----------DVIDS---QEGARASQELHSQEFP----
Castor canadensis                 D---S-----HES--------------SQL-DDH--SVETHSREQS---REHKQKASH----------------E---SREHS-----------DVIDS---QESSKVSQEVHSHEST----
Homo sapiens                      D---SRGKDSYET--------------SQL-DDQ--SAETHSHKQS---RLYKRKAND----------------E---SNEHS-----------DVIDS---QELSKVSREFHSHEFH----
```

Gecko japonicus	-EADHDHDVDND----------HHSVEDNEIEY---
Thamnophis sirtalis	-QLDS--NPDDASNQTVESAESQHSS--------------EDHHSVEHHDSVEDNEV---------------------IL
Meleagris gallopavo	----GSVPAVDTSNQTLESA--EDAEDRHSIENNEVTR
Coturnix japonica	----GSVPAVDASNQTLESA--EDAEDRHSIENNEVTR
Tinamus guttatus	RVSAE-VPDGDNSNQTLESA--EDAQDHLSIKHNEVIF
Anser cygnoides domesticus	SAAASIPTIDDTSNQTLESA--ENAREHHSIENNEVTI
Crocodylus porosus	--SASLPETDADSNQTAESV--EDIQDHHSLEDNEVTI
Chelonian_mydas	-QVDVVPEVND-SNQTAEST--EDTQDHSSIEDNEVIL
Oncorhynchus mykiss	SDSDSAEEAATVATITTDAP-----------------------------------IVITAK---------------
Monopterus albus	--S-----EGTGPDATTDTP-----------------------------------LVITAK---------------
Monodelphis domestica	-------------TQ-----EVNKL--SQEFQSQESHPVN-------DPESVENTKYLKLHSFP--EADSASYEAH
Sarcophilus harrisii	--------SQEFHKQ-----QVGKL--SQEYHSQEVHLVS-------DPESVENIKPLKLHS-H--EVDSASFETH
Condylura cristata	---------------------------SQEFHNQEEKLDL-------DPKSVEEDTHLKFRVSH--ELDSTSSEVN
Felis catus	---------------------------GQTVQSNEKELVQ-------HPESEEQDKHLKFRVSH--ELDSASSEVN
Neomonachus schauinslandi	---------------------------SQIVQSHEKERVL-------DSKSVEEDKHLKFHISH--ELDSASSEVN
Odocoileus virginianus texanus	----------------------------QEFHSLEDKLDL-------DHRSEEEDKHLKIRISH--ELDSTSSEVN
Pantholops hodgsonii	----------------------------QEFHSLEDKLDL-------DHKSE-EDERLKIRISH--ELDSASSEVN
Dasypus novemcinctus	---------------------------SQESHSHEDKLVL-------GPKSKEEDKHLKFHTSH--ELESASSEIN
Orycteropus afer afer	---------------------------SQEFHSQEDKPHR-------DPKSVEEEQHLKFRVSH--ELDSASFETN
Ornithorhynchus anatinus	---DSDEFNRKHYLKSHTSH--EFDSASSETH
Otolemur garnettii	------------------------T--SH-----E------------DLESKEEDKLLKFRVSH--ELDSASSEVN
Manis javanica	-------------------------------FSHEDKLVF-------DPKSREED-KLKFHTSH--ELDSESSEAN
Oryctolagus cuniculus	------------------------Q--SREFRSHEDKLAI-------EPKSEEDEEHRQLRVSH--ELDSTSSEIN
Vicugna pacos	--------------------------------SHEDKLVL-------DPKSEEEDTHLKFRVSH--ELDSASSEVN
Ceratotherium simum simum	--------------------------------SREDKLVL-------DRKSEEEDKYLKFRVSH--ELDSASSEVN
Equus asinus	--------------------------------SSEGKLVR-------DRKSEDEDKYLKFRTFH--ESESASSEVN
Miniopterus natalensis	--------------------------------SYEDKPAL-------HPKSEEQDKHLKFRVSH--ELESASTEVN
Myotis davidii	--------------------------------SHEDKLAS-------DPQSEEQDKHLKFRISH--ELESASSEVN
Trichechus manatus latirostris	--------------------------------SREDKYYP-------DPKSEEEDKHLKFRISH--ELES------
Heterocephalus glaber	--------------------------------SH-----E-------DPKSKEEDEHLKIRVSH--ELDSVSSEAN
Castor canadensis	--------------------------------SLEDKPVL-------DPKSQKEDKHLKYRISH--ELDSASSEVN
Homo sapiens	--------------------------------SHEDMLVV-------DPKSKEEDKHLKFRISH--ELDSASSEVN

Table S2.2. Alignment of representative sequences.

Table S3: Predicted osteopontin sequences in birds. For most avian osteopontin sequences, a long (complete) and short form of the protein are predicted. The records that have NM_ or XM_ 6 or 12+ digit formats, are reference sequences (RefSeqs), which are curated from single or multiple sequence records that have been directly submitted to GenBank. KK and KL indicate NCBI CON division sequences. JJ are Genbank GSS sequences.

bird	complete	short
Acanthisitta chloris	XM_009073853.1	KK835285.1
Apaloderma vittatum	XM_009868641.1	KL382114.1
Aptenodytes forsteri	XM_009280227.1	KL226085.1
Balearica regulorum gibbericeps	XM_010311221.1	KL502968.1
Buceros rhinoceros silvestris	XM_010145609.1	KL509681.1
Calypte anna	XM_008500834.1	KL218525.1
Caprimulgus carolinensis	XM_010173470.1	KL358285.1
Cariama cristata	XM_009694723.1	KK508828.1
Chaetura pelagica	XM_009993754.1	KN125683.1
Charadrius vociferus	XM_009890748.1	KL873122.1
Chlamydotis macqueenii	XM_010119303.1	KK749833.1
Colius striatus	XM_010202192.1	KK544289.1
Corvus brachyrhynchos	NW_008240042.1	KK719673.1
Cuculus canorus	XM_009559526.1	KL447603.1
Egretta garzetta	XM_009639993.1	KK501660.1
Eurypyga helias	XM_010154680.1	KK572170.1
Fulmarus glacialis	XM_009584852.1	KK609601.1
Gavial stellata	XM_009813620.1	JJRM01070990.1
Haliaeetus albicilla	XM_009916485.1	KK651471.1
Leptosomus discolor	XM_009958978.1	KK689572.1
Manacus vitellinus	XM_008930247.2	KL672548.1
Merops nubicus	XM_008942236.1	KK707889.1
Mesitornis unicolor	XM_010181850.1	KK807537.1
Nestor notabilis	XM_010019624.1	KK945799.1
Nipponia nippon	XM_009467655.1	KL409714.1
Opisthocomus hoazin	XM_009932280.1	KK734416.1
Pelecanus crispus	XM_009490811.1	KK496753.1
Phaethon lepturus	XM_010287426.1	JJRF01066258.1
Phalacrocorax carbo	XM_009512482.1	KL444864.1
Picoides pubescens	XM_009904975.1	KL216617.1
Pterocles gutturalis	XM_010079868.1	KL241686.1
Pygoscelis adeliae	XM_009327481.1	KL225236.1
Struthio camelus australis	XM_009676586.1	KL206412.1
Tauraco erythrolophus	XM_009978083.1	KL455818.1
Tinamus guttatus	XM_010218705.1	KL892771.1
Tyto alba	XM_009970430.1	KK369619.1

Anas platyrhynchos	XM_005012779.3	
Anser cygnoides domesticus	XM_013190510.1	
Apteryx australis mantelli	XM_013957376.1	
Aquila chrysaetos canadensis	XM_011600806.1	
Calidris pugnax	XM_014961493.1	
Columba livia	XM_005498492.2	
Corvus cornix cornix	XM_010412044.2	
Coturnix coturnix japonica	AF239805.1	
Coturnix japonica	NM_001323234.1	
Falco cherrug	XM_005444503.1	
Falco peregrinus	XM_005233595.1	
Ficedula albicollis	XM_016297843.1	
Galeopterus variegatus	XM_008574253.1	
Gallus	M59182.1	
Geospiza fortis	XM_005418195.1	
Haliaeetus leucocephalus	XM_010569846.1	
Lepidothrix coronata	NW_016690259.1	
Limosa lapponica baueri	KZ506572.1	
Lonchura striata domestica	XM_021526515.1	
Meleagris gallopavo	XM_003205565.3	
Meleagris gallopavo	NC_015014.2	
Melopsittacus undulatus	XM_005146580.1	
Numida meleagris	XM_021395699.1	
Parus major	XM_015623543.2	
Patagioenas fasciata monilis	LSYS01006159.1	
Pseudopodoces umilis	XM_005525626.2	
Serinus canaria	NW_007931124.1	
Sturnus vulgaris	XM_014878941.1	
Taeniopygia guttata	XM_002190366.1	
Zonotrichia albicollis	XM_005485296.1	
Cathartes aura		KL320674.1
Phoenicopterus ruber ruber		KK415766.1
Podiceps cristatus		KL257771.1

Figure S1: The domain structure of osteopontin. A search of 198 osteopontin sequences was conducted in Meme Suite with the expectation of 13 motifs (to not exceed the capacity of the software, partial sequences and two bird sequences needed to be eliminated). **A)** List of the identified motifs with statistics. Motifs with a pale red background are very similar to earlier specified motifs. The colored squares in the right column match the color coding in part B) of this Figure. **B)** Top scoring sequences, each of which has an E-value less than 10. The motif matches shown have a position p-value less than 0.0001. The hit p-value is not adjusted for the length of the sequence. Note the similarity to the domain structure identified in Figure 1.

MOTIFS

	Logo	Name	Alt. Name	Width	1.	2.	3.	4.	5.	6.	7.	8.	9.	10.	11.	12.	13.	
1.	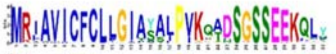	MRIAVICFCLLGIAYALPVKQADSGSSEEKQLY	MEME-1	33	--	0.45	0.17	0.19	0.22	0.14	0.13	0.19	0.12	0.1	0.17	0.95	0.11	
2.	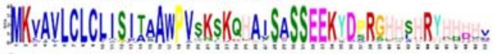	MKVAVLCLCLISITAAWPVSKSKQHAISASSEEKYDPRGHHSHRYHHHHV	MEME-2	50	0.45	--	0.17	0.16	0.21	0.24	0.14	0.13	0.1	0.16	0.16	0.83	0.12	
3.	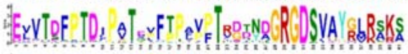	EVVTDFPTDIPATEVFTPAVPTRDTNDGRGDSVAYGLRSKS	MEME-3	41	0.17	0.17	--	0.17	0.91	0.18	0.16	0.15	0.09	0.17	0.86	0.21	0.14	
4.	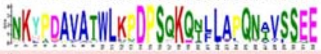	NKYPDAVATWLKPDPSQKQTLLAPQNAVSSEE	MEME-4	32	0.19	0.16	0.17	--	0.16	0.83	0.19	0.17	0.14	0.12	0.2	0.18	0.12	
5.	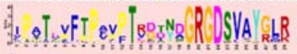	IPATEVFTPAVPTRDTNDGRGDSVAYGLR	MEME-5	29	0.22	0.21	0.91	0.16	--	0.16	0.13	0.16	0.07	0.13	0.72	0.2	0.12	
6.	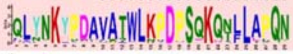	QLYNKYPDAVATWLKPDPSQKQNLLAPQN	MEME-6	29	0.14	0.24	0.18	0.83	0.16	--	0.15	0.13	0.13	0.12	0.17	0.21	0.11	
7.	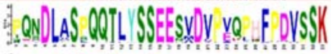	PQNDLASPQQTLYSSEESVDVPVQPHFPDVSSK	MEME-7	33	0.13	0.14	0.16	0.19	0.13	0.15	--	0.2	0.16	0.13	0.13	0.17	0.12	
8.	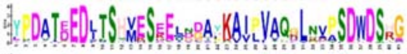	YPDATEEDJTSHVESEELBDAYKAIPVAQGLNVPSDWDSHG	MEME-8	41	0.19	0.13	0.15	0.17	0.16	0.13	0.2	--	0.17	0.17	0.16	0.26	0.13	
9.	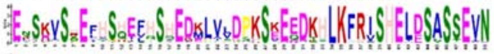	ENSKVSHEFHSHEFHSHEDKLVLDPKSKEEDKHLKFRISHELDSASSEVN	MEME-9	50	0.12	0.1	0.09	0.14	0.07	0.13	0.16	0.17	--	0.19	0.12	0.18	0.11	
10.	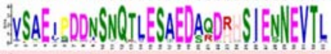	VSAEIPDDNSNQTLESAEDAQDRHSIENNEVTL	MEME-10	33	0.1	0.16	0.17	0.12	0.13	0.12	0.13	0.17	0.19	--	0.16	0.1	0.16	
11.	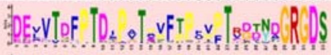	DEVVTDFPTDIPATPVFTPFVPTRDTNDGRGDS	MEME-11	33	0.17	0.16	0.86	0.2	0.72	0.17	0.13	0.16	0.12	0.16	--	0.19	0.1	
12.	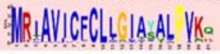	MRIAVICFCLLGIAYALPVKQ	MEME-12	21	0.95	0.83	0.21	0.18	0.2	0.21	0.17	0.26	0.18	0.1	0.19	--	0.13	
13.	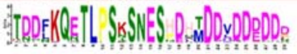	TDDFKQETLPSKSNESHDHTDDVDDDDDD	MEME-13	29	0.11	0.12	0.14	0.12	0.12	0.11	0.12	0.13	0.11	0.16	0.1	0.13	--	

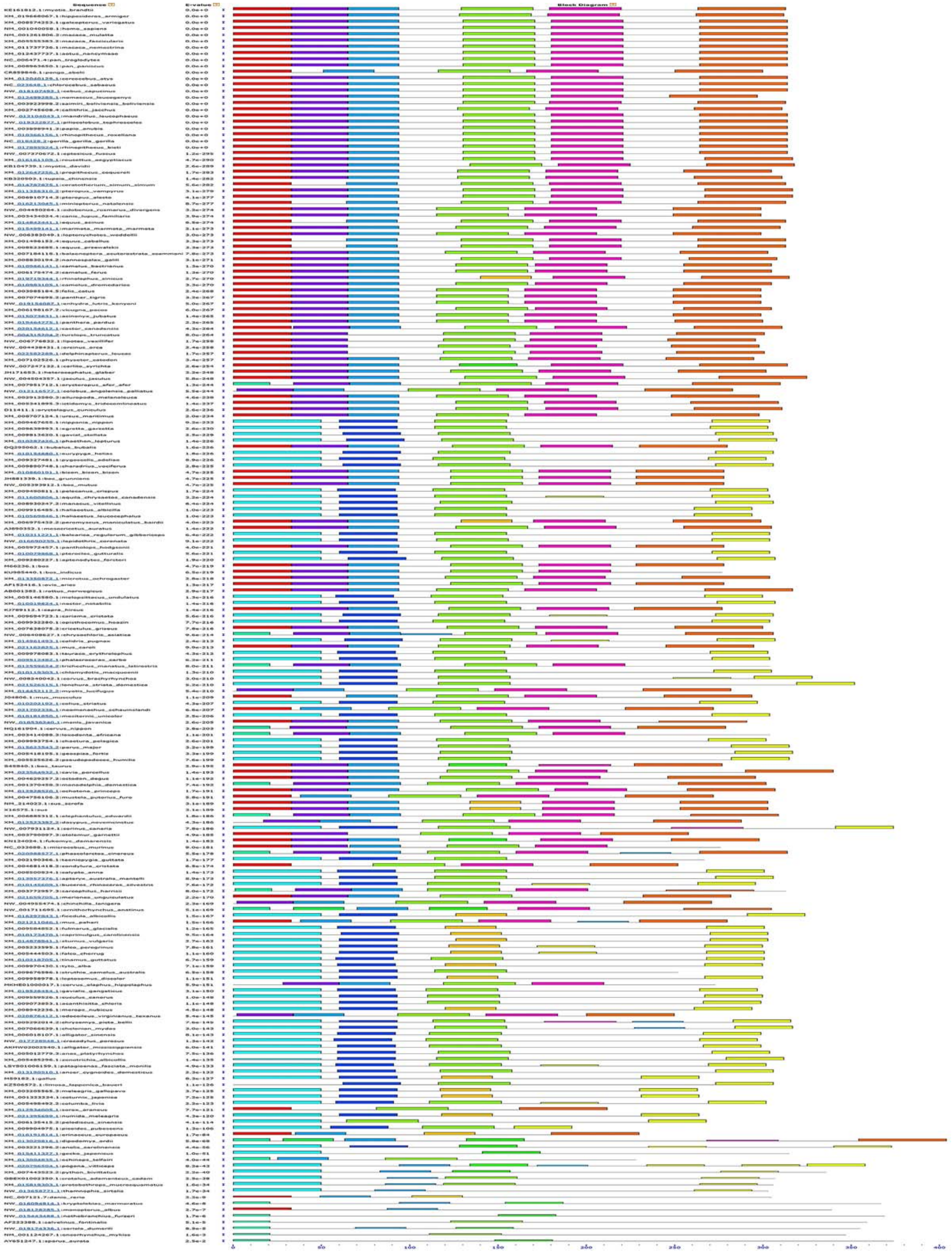

Sequence
E-value
Block Diagram
0
50
100
150
200
250
300
350
400

Figure S2: Phylogenetic tree of the canonical osteopontin sequences. The canonical sequences derived from Tables S1 and S2 were used for a taxonomic analysis. In positions with amino acid variation, either the most common allele or X served as placeholders. The tree was generated using the TreeTop option in GeneBee.

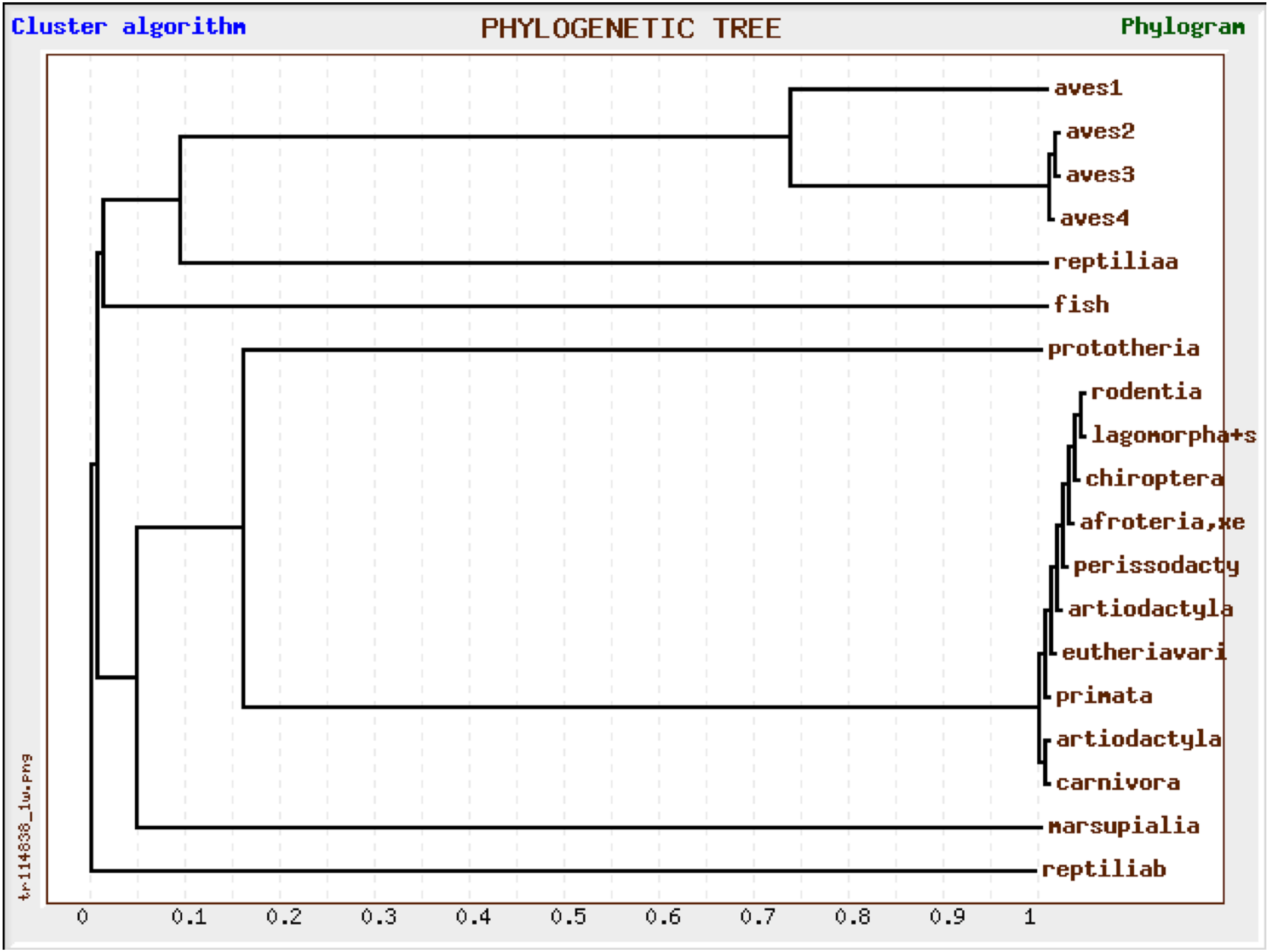

Cluster algorithm
PHYLOGENETIC TREE
Phylogram
aves1
aves2
aves3
aves4
reptiliaa
fish
prototheria
rodentia
lagomorpha+s
chiroptera
afroteria,xe
perissodacty
artiodactyla
eutheriavari
primata
artiodactyla
carnivora
marsupialia
reptiliab
0
0.1
0.2
0.3
0.4
0.5
0.6
0.7
0.8
0.9
1

Figure S3: The amino acid composition of osteopontin. Shown are the amino acid compositions (as percent of total) for nine orders of animals. Each bar represents one species, all species available in each order were analyzed. There is little variation within each order, but notable differences among orders of animals. All osteopontins are rich in serine and low in tyrosine. The asparagine content is high (lowest, by comparison, in Marsupials). Squamata have a high histidine content due to the long insert between the SSEE motifs.

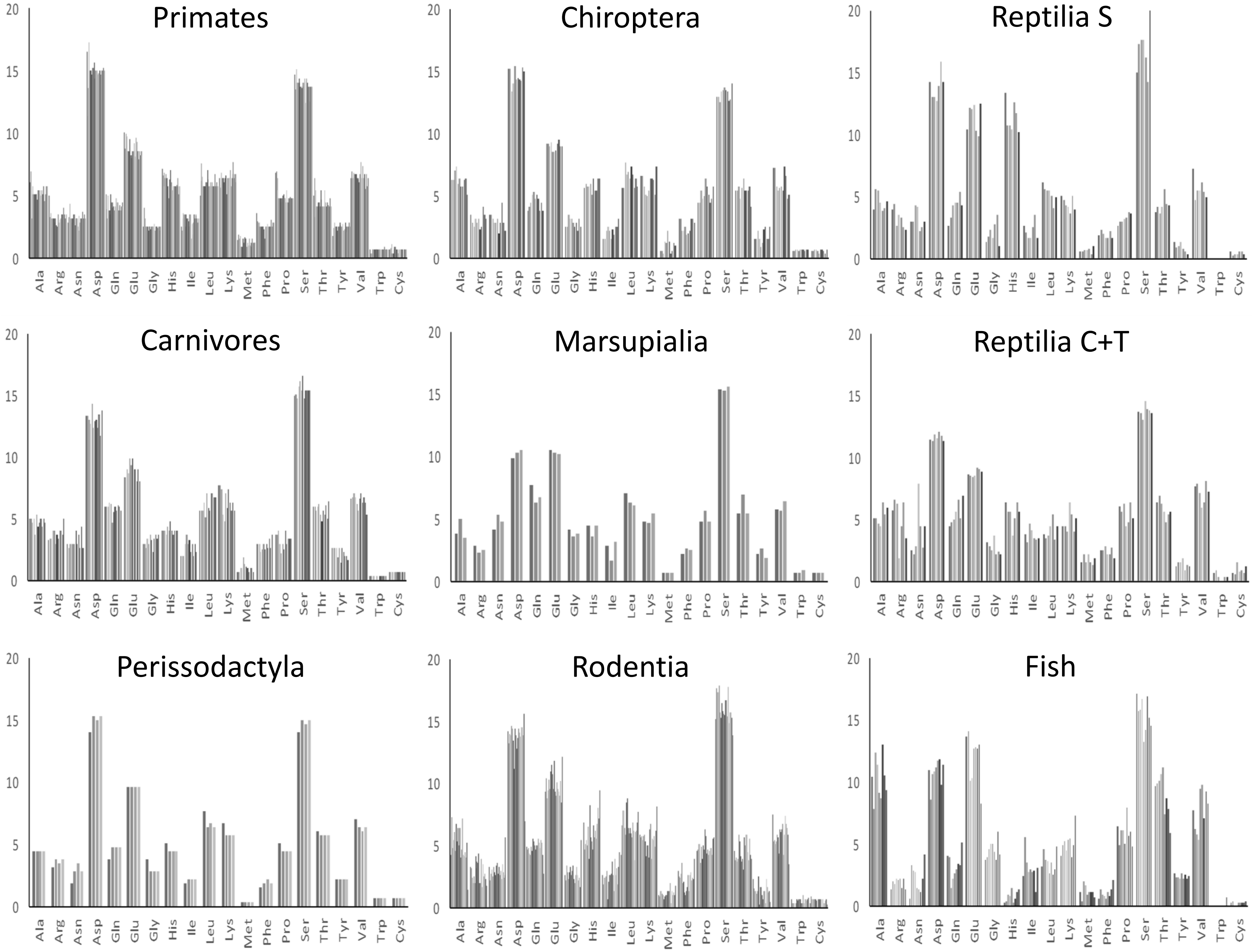

Primates
Chiroptera
Reptilia S
Carnivores
Marsupialia
Reptilia C+T
Perissodactyla
Rodentia
Fish
0
5
10
15
20
Ala
Arg
Asn
Asp
Gln
Glu
Gly
His
Ile
Leu
Lys
Met
Phe
Pro
Ser
Thr
Tyr
Val
Trp
Cys
